# New 5-Hydroxycoumarin-Based Tyrosyl-DNA Phosphodiesterase I Inhibitors Sensitize Tumor Cell Line to Topotecan

**DOI:** 10.3390/ijms24119155

**Published:** 2023-05-23

**Authors:** Tatyana M. Khomenko, Alexandra L. Zakharenko, Tatyana E. Kornienko, Arina A. Chepanova, Nadezhda S. Dyrkheeva, Anastasia O. Artemova, Dina V. Korchagina, Chigozie Achara, Anthony Curtis, Jóhannes Reynisson, Konstantin P. Volcho, Nariman F. Salakhutdinov, Olga I. Lavrik

**Affiliations:** 1 N. N. Vorozhtsov Novosibirsk Institute of Organic Chemistry, Siberian Branch of the Russian Academy of Sciences, 9, Akademika Lavrentieva Ave., 630090 Novosibirsk, Russia; chomenko@nioch.nsc.ru (T.M.K.);; 2 Institute of Chemical Biology and Fundamental Medicine, Siberian Branch of the Russian Academy of Sciences, 630090 Novosibirsk, Russiadyrkheeva.n.s@gmail.com (N.S.D.);; 3 School of Pharmacy and Bioengineering, Keele University, Hornbeam Building, Newcastle-under-Lyme, Staffordshire ST5 5BG, UKa.d.m.curtis@keele.ac.uk (A.C.); j.reynisson@keele.ac.uk (J.R.)

**Keywords:** coumarin, monoterpene, enzyme inhibition, DNA repair, synergy

## Abstract

Tyrosyl-DNA-phosphodiesterase 1 (TDP1) is an important enzyme in the DNA repair system. The ability of the enzyme to repair DNA damage induced by a topoisomerase 1 poison such as the anticancer drug topotecan makes TDP1 a promising target for complex antitumor therapy. In this work, a set of new 5-hydroxycoumarin derivatives containing monoterpene moieties was synthesized. It was shown that most of the conjugates synthesized demonstrated high inhibitory properties against TDP1 with an IC_50_ in low micromolar or nanomolar ranges. Geraniol derivative **33a** was the most potent inhibitor with IC_50_ 130 nM. Docking the ligands to TDP1 predicted a good fit with the catalytic pocket blocking access to it. The conjugates used in non-toxic concentration increased cytotoxicity of topotecan against HeLa cancer cell line but not against conditionally normal HEK 293A cells. Thus, a new structural series of TDP1 inhibitors, which are able to sensitize cancer cells to the topotecan cytotoxic effect has been discovered.

## 1. Introduction

Tyrosyl-DNA phosphodiesterase 1 (TDP1) is an important target in cancer therapy because it repairs damaged 3′ ends of DNA caused by various drugs used commonly in cancer chemotherapy [1]. These damages include the covalent adduct of topoisomerase 1 (Top1) and DNA, so-called Top1cc (Top1/DNA cleavage complex), which is formed during the normal catalytic action of Top1 and is stabilized by the antitumor drugs topotecan and irinotecan [2]. Such a “stuck” complex leads to the formation of double-strand breaks and cell death. TDP1 eliminates Top1cc, thereby interfering with the action of clinically used drugs and being one of the causes of resistance to them [3,4].

**Figure 1:**
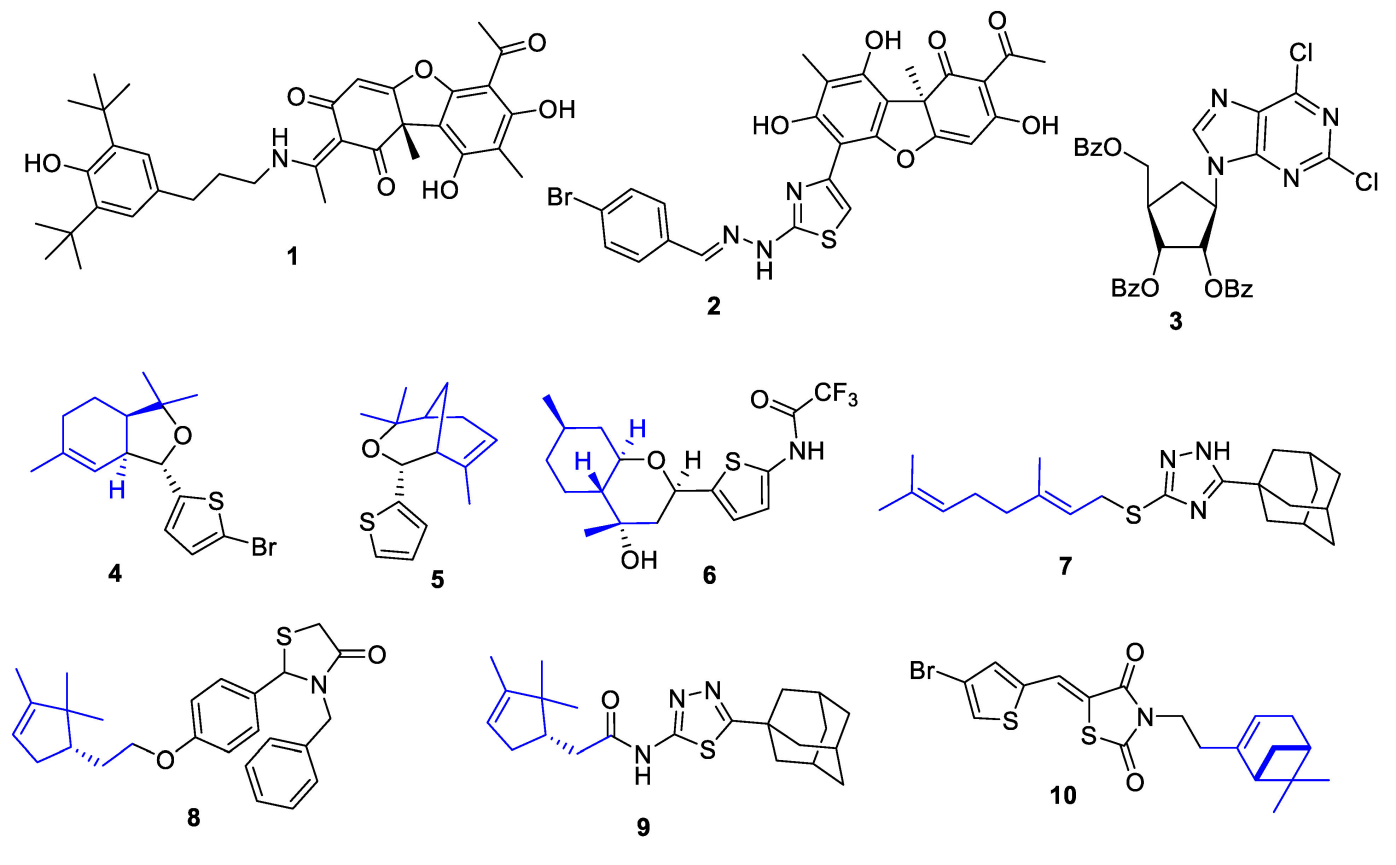
Some known TDP1 inhibitors (**1**–**10**). Monoterpene fragments are highlighted in blue.

**Figure 2:**
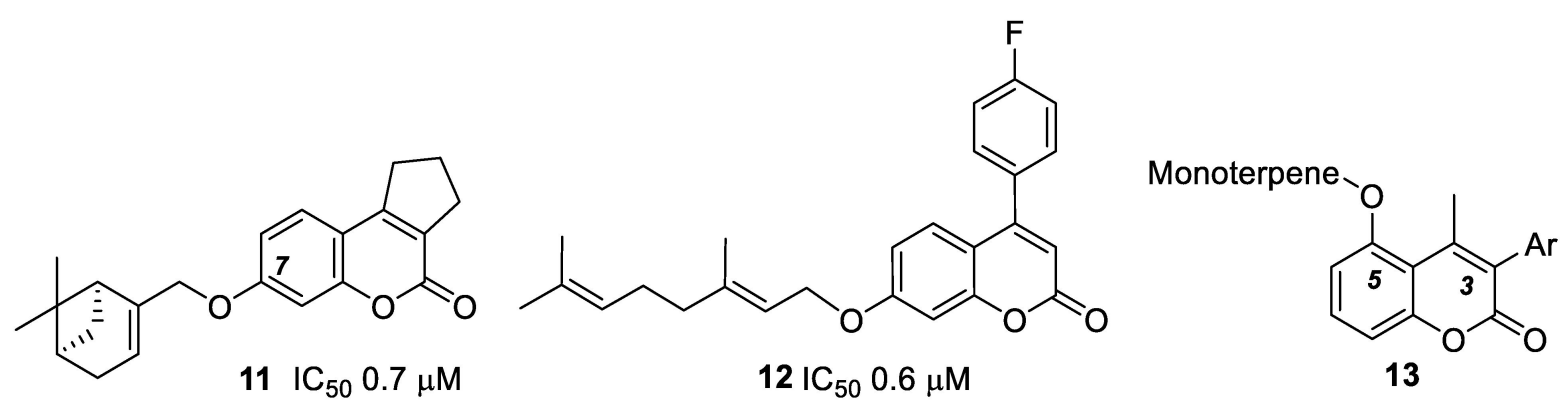
Previously found coumarin-based TDP1 inhibitors and general formula of target compound.

**Figure 3:**
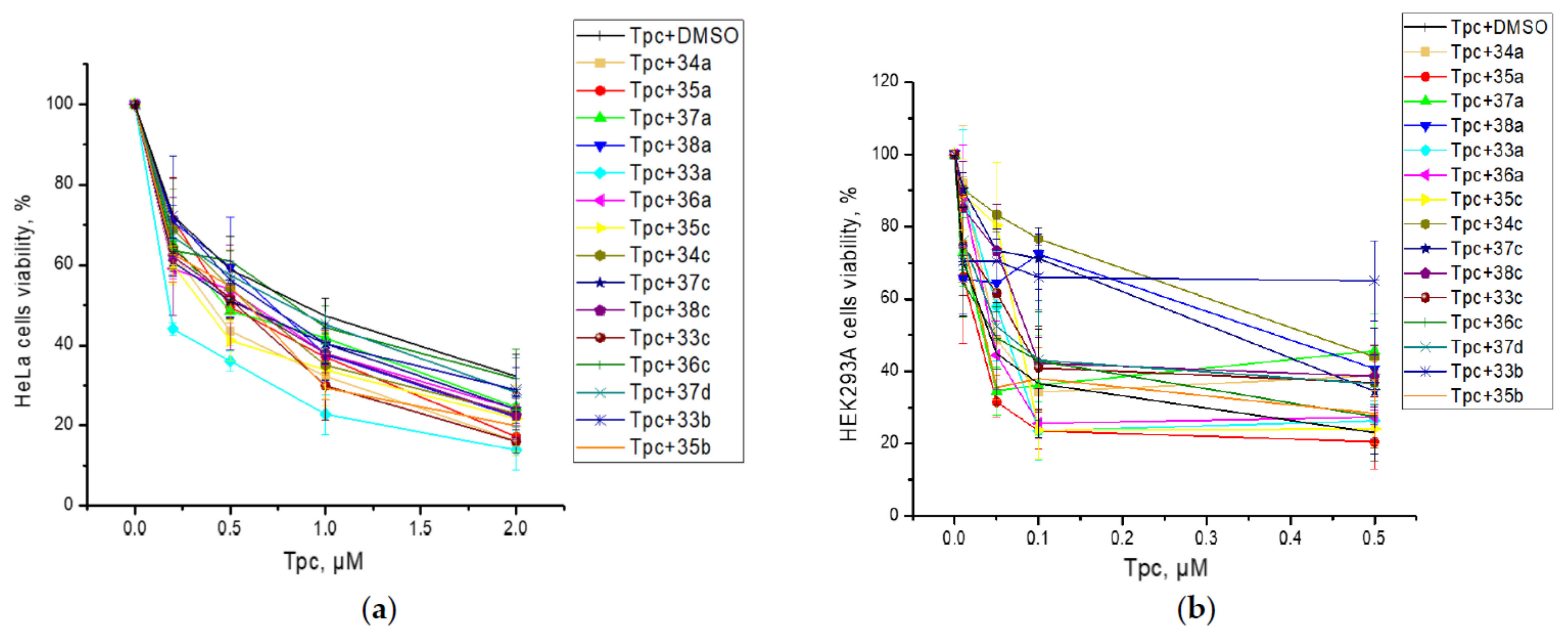
Effect of 5-hydroxycoumarin derivatives (10 µM) on topotecan cytotoxicity against HeLa (**a**) and HEK293A (**b**) cells. The cytotoxicity of the compounds was studied by the metabolic activity of cells using the MTT test.

Thus, TDP1 inhibitors could increase the efficacy of therapy with Top1 inhibitors such as topotecan and irinotecan and/or reduce the dose, and hence toxicity, of the latter. Indeed, there is a large amount of experimental evidence for this: cells or animals deficient in TDP1 are more sensitive to Top1 inhibitors [5,6,7,8,9]. Conversely, increased expression of TDP1 protects tumor cells from these drugs [10,11,12,13,14]. In addition, our team demonstrated the effectiveness of TDP1 inhibitors as sensitizers of the action of topotecan against a number of mouse tumors in vivo. These compounds belong to usnic acid derivatives (Figure 1, **1** and **2**) [15,16,17] and nucleosides (Figure 1, **3**) [18]. Nevertheless, no TDP1 inhibitors are in clinical trials now, making the search for new inhibitors of this enzyme important. Thus, several new structural types of TDP1 inhibitors were developed recently [19,20,21].

A number of good TDP1 inhibitors were developed using natural compounds [22] including monoterpenes. For example, compounds **4**–**6** (Figure 1) were synthesized by reactions of monoterpenes with aldehydes [23,24]. In contrast, inhibitors **7**–**10** (Figure 1) contain monoterpene fragments without significant modification of their core [25,26,27,28].

Previously, we have shown that 7-hydroxycoumarin derivatives with monoterpene moieties **11** and **12** (Figure 2) inhibit TDP1 in the submicromolar concentration range and sensitize the action of camptothecin (a Top1 inhibitor of natural origin) [29] and topotecan on the Krebs-2 ascites in vivo tumor model [30]. Of note here is that many coumarins demonstrated diverse biological activity [31,32,33]. Although it is known that some coumarins are toxic and can induce hepatotoxicity, for example [34], usually they are safe enough for pharmacological application [35]. Based on molecular modeling, it was suggested that analogues of these compounds containing an oxyterpene residue at position 5 of coumarin and having an aryl fragment at position 3 of the model structure **13** (Figure 2) could have high inhibitory activity.

In order to verify this assumption experimentally, novel monoterpene esters of 5-hydroxycoumarins containing aromatic substituents in the 3-position of the pyran ring were synthesized. Substituted 3-arylylcoumarins has various pharmacological applications and could be considered as a privileged scaffold in medicinal chemistry [36]. It has been shown that these compounds are indeed capable of effectively inhibiting TDP1, as well as sensitizing tumor cell lines to the action of topotecan.

## 2. Results and Discussion

### 2.1. Chemistry

Target compounds were synthesized by condensation of substituted phenylacetic acids with 2,6-dihydroxyacetophenone, followed by removal of the acetyl group and subsequent reaction with monoterpene bromides.

**Scheme 1:**
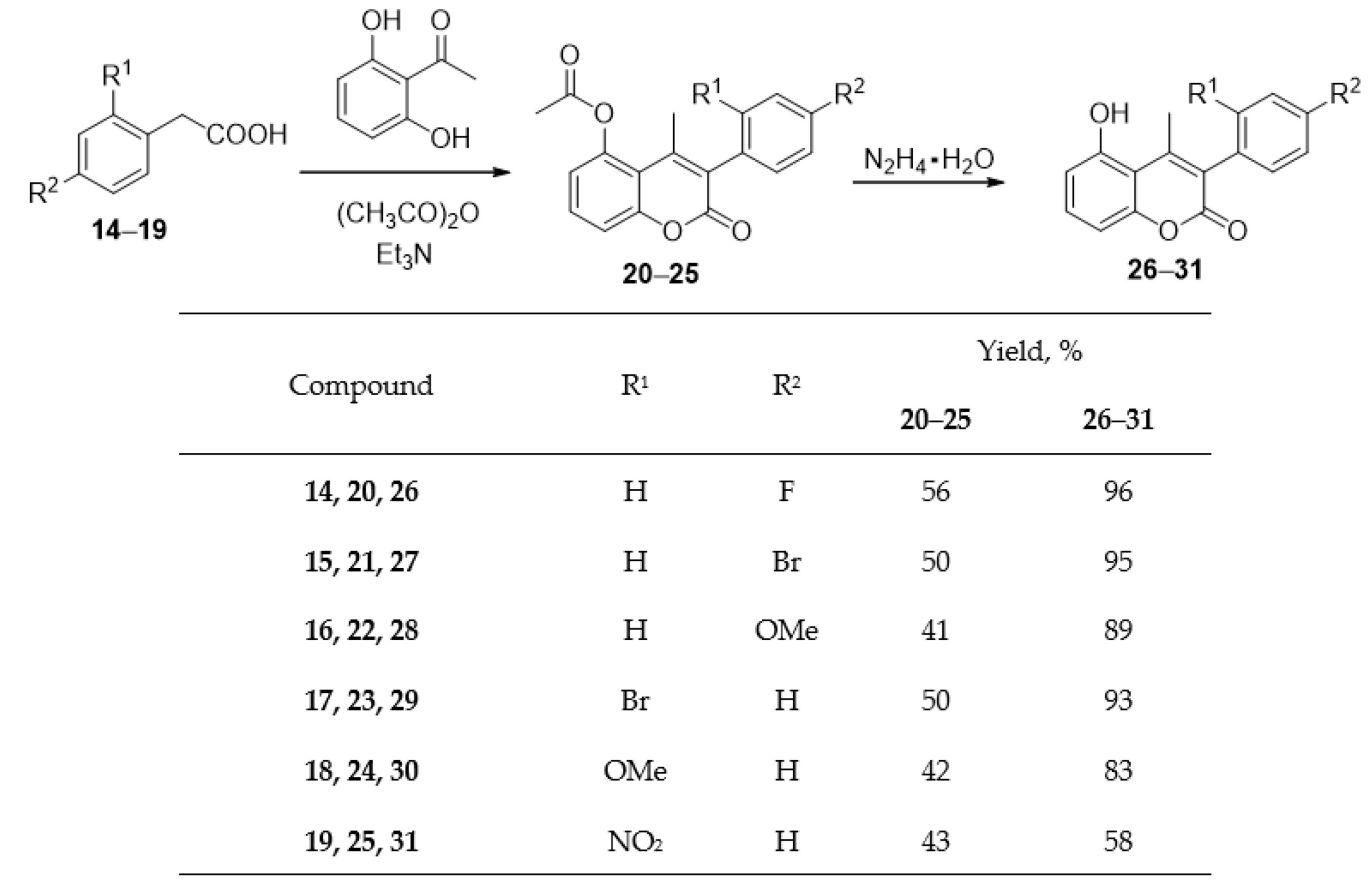
Synthesis of 5-hydroxy-3-arylcoumarins **26**–**31**.

The acetyloxycoumarins **20**–**25** were obtained via a modified Perkin-Oglialoro condensation reaction of corresponding phenylacetic acid **14**–**19** with 2,6-dihydroxyacetophenone in acetic anhydride in the presence of triethylamine in accordance with methods [37,38]. Compounds **20**–**25** were treated with hydrazine monohydrate to give 5-hydroxy-3-arylcoumarins **26**–**31** (Scheme 1).

**Scheme 2:**
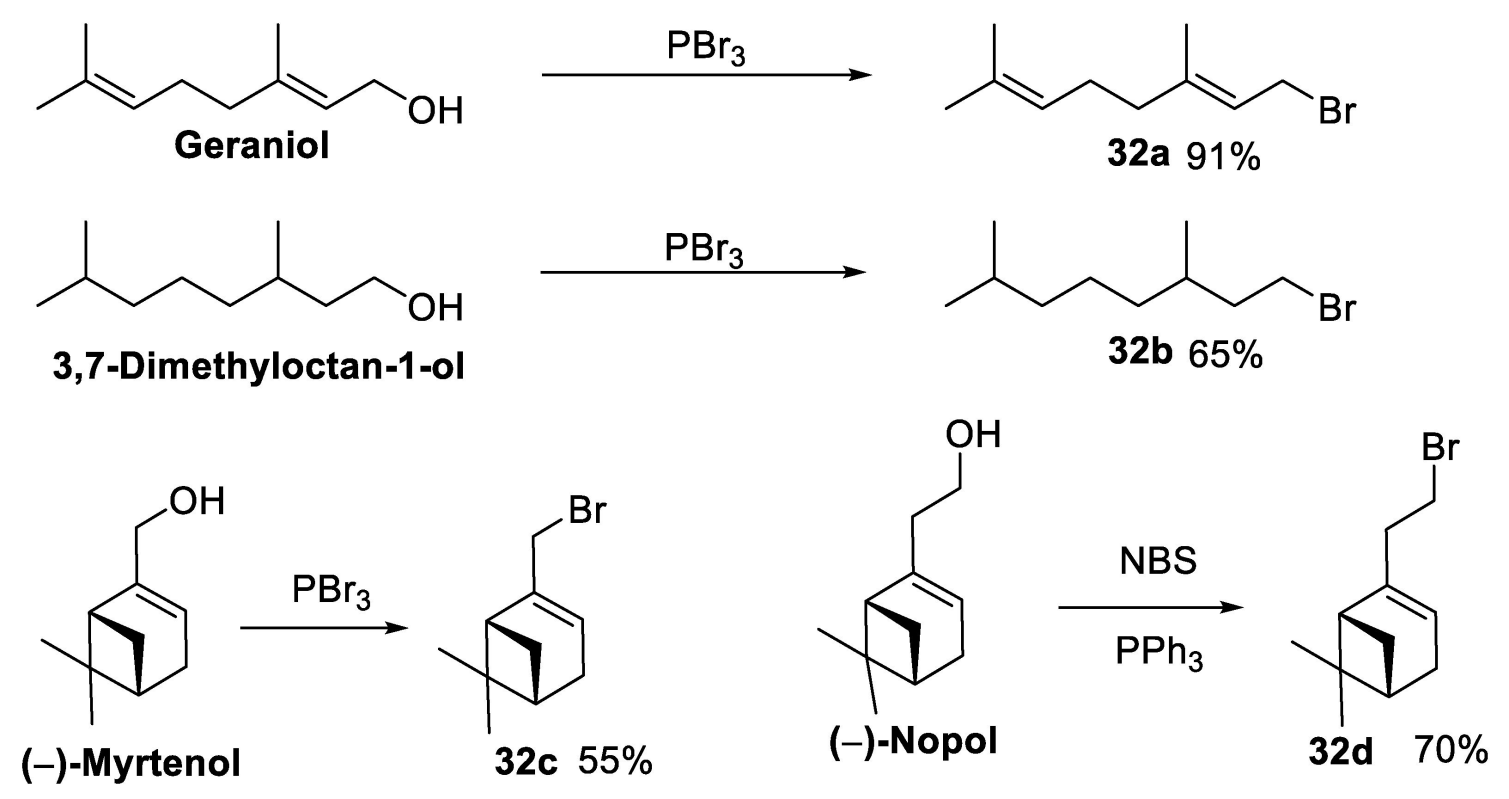
Synthesis of monoterpenoid bromides **32a**–**d**.

Bromides **32a**–**d** were obtained by the reaction of monoterpene alcohols (-)-myrtenol and its homologue (-)-nopol, geraniol and 3,7-dimethyloctan-1-ol with PBr_3_ or NBS/PPh_3_ (Scheme 2).

**Scheme 3:**
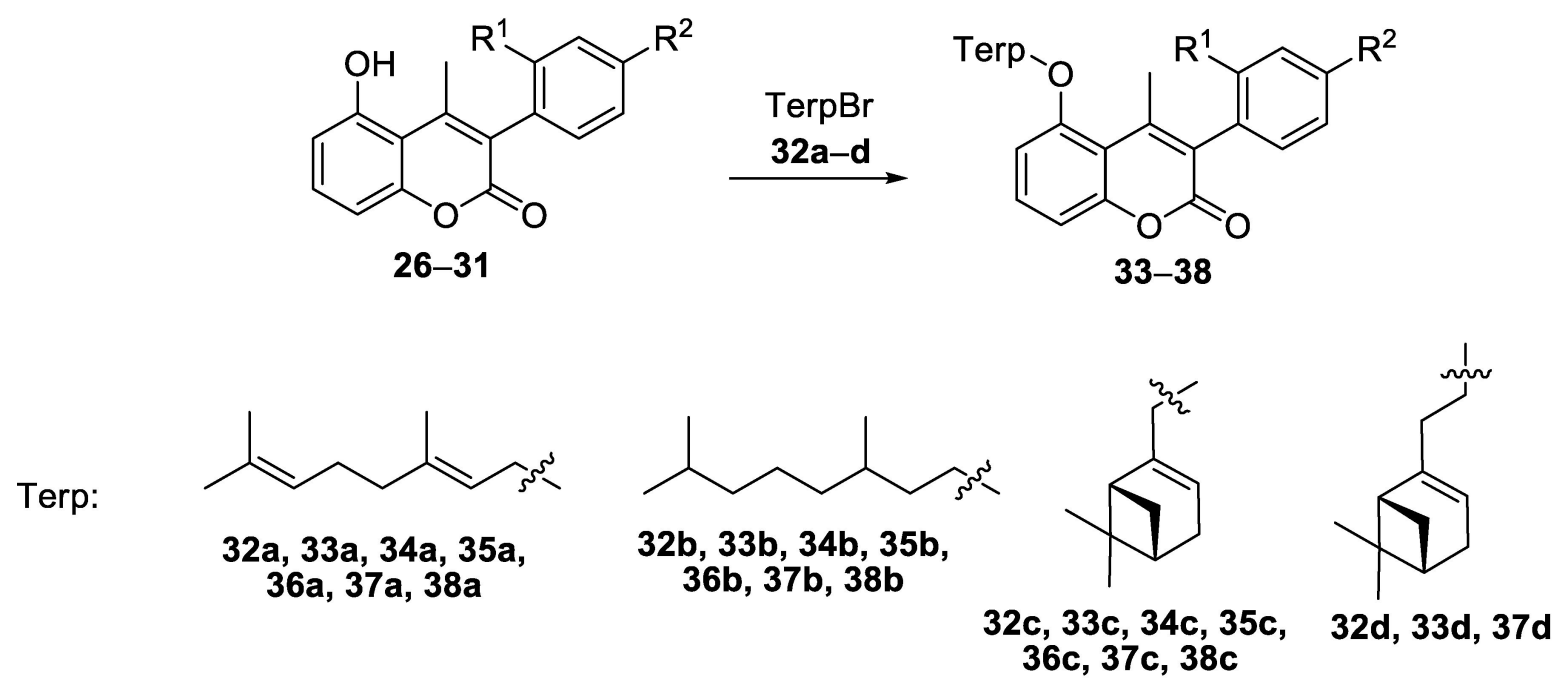
Synthesis of monoterpenoid substituted 5-hydroxy-3-arylcoumarins **33**–**38**.

Coumarin-monoterpene conjugates **33**–**38** were obtained by reaction of 5-hydroxycoumarins **26**–**31** with corresponding monoterpenoid bromides **32a**–**d** in the presence of DBU and DMF, as described previously [39,40] (Scheme 3). The products were obtained in yields 44–89% following purification by either recrystallization or column chromatography.

### 2.2. Biology

To determine the inhibitory properties of the new compounds against TDP1 we used a previously designed [41] real-time oligonucleotide biosensor with 5(6)-carboxyfluorescein (FAM) at the 5′ end and fluorophore quencher BHQ1 (Black Hole Quencher-1) at the 3′-end.

As can be seen from Table 1, all geraniol derivatives **33a**–**38a** demonstrated good inhibitory activity against TDP1 with IC_50_ values in low micromolar or submicromolar ranges. The most active compound with IC_50_ 130 nM contains a fluorine substituent in the 4-position of the pendant aromatic ring. Methoxy substituted derivatives were slightly less active than their halogen containing counterparts. The introduction of a nitro group did not lead to significant changes in inhibitory activity.

Compound **33b** containing a fully saturated monoterpenoid fragment was twofold less active than its analogue **33a**. It is interesting that in this case the 4-methoxy substituted compound also demonstrated high inhibitory activity.

Among the myrtenol-coumarin conjugates, the most active were with the derivative bearing 4-bromo (**34c**) and 2-methoxy (**37c**) substituents on the pendant aryl ring. Elongation of the linker when passing from conjugate **37c** to conjugate **37d** led to a decrease in inhibitory activity.

Thus, monoterpene esters of 5-hydroxycoumarins containing aromatic substituents in the 3-position of the pyran ring indeed demonstrated good inhibitory activity against TDP1 as we proposed at the start of our work, with compound **33a** being the most active.

It is interesting that 5-hydroxycoumarin derivatives bearing an acyclic monoterpene fragment were generally more active than their counterparts containing a bicyclic monoterpene substituent, whilst earlier studies [30] demonstrated that 7-hydroxycoumarin conjugates with both types of monoterpene residue had comparable inhibitory activity. Taking into account that most active 7-hydroxycoumarin derivatives have IC_50_ not less than 0.4 μM [29,30], the new coumarin-monoterpene conjugates reported here are the most potent TDP1 coumarin-based inhibitors found so far.

The cytotoxicity of the compounds was studied against the cell lines HeLa (cervical carcinoma) and HEK293A (human embryonic kidney). Since TDP1 inhibitors are planned to be used in combination with other chemotherapy drugs that have significant side effects, the toxicity of these TDP1 inhibitors should be minimal. Overall, the compounds exhibited moderate to mild toxicity (see Table 2 and Figure S1 in Supplementary), the semi-toxic concentration CC_50_ was 60 µM or more for both tested cell lines. Compounds **34a** and **37a** turned out to be the most toxic for HeLa cells (CC_50_ values 32 and 18 µM, respectively). For cells of non-cancerous origin, HEK293A, compound **37a** was also the most toxic (16 µM), while **33b** and **38c** were somewhat more toxic than the rest of the compounds (40 and 32 µM, respectively). The most effective inhibitor of TDP1 **33a** turned out to be one of the least toxic compounds (turquoise plot in Figure S1 in the Supplementary Information).

To study the effect of our compounds on the cytotoxic effect of topotecan, we chose a low-toxic concentration of TDP1 inhibitor of 10 µM. With respect to HeLa cervical cancer cells, many compounds increased the cytotoxicity of topotecan (Figure 3a), with **33a** being the most effective sensitizer (Table 2).

With respect to cells of non-cancerous origin HEK293A, no pronounced sensitizing effect of 5-hydroxycoumarins was found (Table 2, Figure 3b). Moreover, some compounds (e.g., **38a**, **34c**, **37c**) protect cells from the cytotoxic effects of topotecan. Previously, we also observed the absence of sensitization of the cytotoxic effect of topotecan by TDP1 inhibitors of a different structure on HEK293A cells [26].

Thus, the most active TDP1 inhibitor demonstrating the promising sensitization effect, compound **33a,** significantly increased cytotoxicity of topotecan against HeLa cancer cell line but not against conditionally normal HEK293A cells.

### 2.3. Molecular Modelling

All the ligands were modelled against TDP1′s binding pocket (PDB ID: 6W7K, resolution 1.70 Å) [42], which reliability was previously established [43]. The four scoring functions incorporated in the GOLD (v2020.2.0) software were used: GoldScore(GS) [44], ChemScore(CS) [45,46], ChemPLP(Piecewise Linear Potential) [47] and ASP(Astex Statistical Potential) [48]. Furthermore, the GOLD package is thoroughly benchmarked for its reliability [49,50]. In Table S1 all the scores for the ligands are given and they have reasonable values. The scores were correlated against their measured IC_50_ values and only weak trends were observed (ASP (R^2^–0.039), ChemPLP (R^2^–0.147), GS (R^2^–0.007) and CS (R^2^–0.094)).

**Figure 4:**
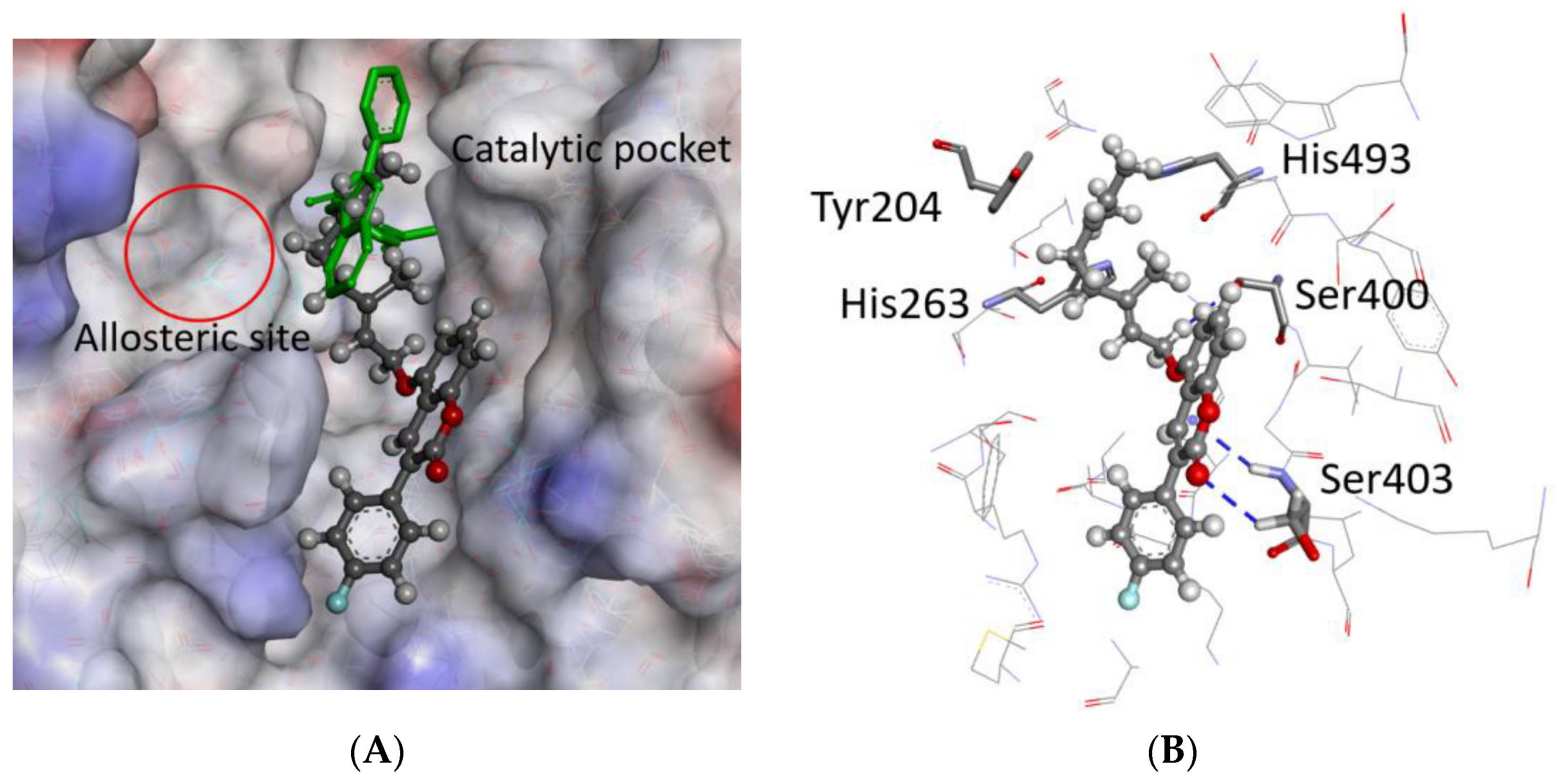
The predicted binding (ChemPLP) of **33a** in the catalytic pocket of TDP1 (**A**) The ligand is in the ball-and-stick format and the co-crystallized ligand is shown as green sticks. The potential allosteric pocket is shown in the circled area. The protein surface is rendered; blue depicts regions with a partial positive charge on the surface; red depicts regions with a partial negative charge and grey shows neutral areas. (**B**) Weak H-boding was predicted between the amino acids Ser400 and Ser403 (stick format) shown as blue dashed lines. The catalytic His263 and His493 amino acid residues are also depicted as sticks as well as Tyr204 making up the allosteric binding site. The amino acids within 5 Å of the ligand are shown as lines. The hydrogens of the amino acids are not shown for clarity.

When the predicted binding poses of the compounds were analyzed no dominant configurations were seen. However, the ligands overlapped with the co-crystallized ligand and were predicted to sit in the catalytic pocket with the catalytic His263 and His493 amino acid residue except for **35c**. The configuration predicted by ChemPLP, of **33a** is shown in Figure 4. The aliphatic chain moiety overlaps with the co-crystallized ligand whereas the coumarin and the para fluorophenyl rings are sitting in a groove. The methylene terpene carbon from the oxygen coming off the 5′ position of the coumarin forms a weak H-bond with the hydroxyl side chain of the Ser400 amino acid residue. In addition, Ser403 forms one weak hydrogen bond from its side chain methylene with the carbonyl group in the coumarin ring system as well as hydrogen–π ring system interaction between the backbone’s amino group and the ester containing ring (see Figure 4B).

Structural activity relations and modelling studies of usnic acid derivatives suggest an allosteric binding site close to the catalytic pocket as shown in Figure 4A [43]. This potential allosteric site was also suggested by molecular dynamics simulation work [51]. In this study, none of the ligands were predicted to reach this potential pharmacophore, which may explain their relatively modest efficacy.

### 2.4. Chemical Space

The mainstream molecular descriptors water-octanol partition coefficient (log *P*), molecular weight (MW, g mol^−1^), hydrogen bond donors (HD), hydrogen bond acceptors (HA), rotatable bonds (RB) and polar surface area (PSA, Å^2^) are given in Table S2. The values of the descriptors span different definitions of chemical space: HD are in lead-like chemical space; PSA is both in lead- and drug-like space; and in drug-like space only are RB, MW and HA. Log *P* is in Known Drug Space (KDS) and beyond (see definitions for regions in chemical space see ref. [52] and in Table S3).

To find the balance of the physicochemical properties reflected in the molecular descriptors described above the Known Drug Indexes (KDIs) for the ligands were derived. The KDIs are derived from drugs in clinical use, i.e., a weighted index from a normalized (to a maximum of 1) statistical distribution was fitted to a Gaussian function for each descriptor. Both the summation of the indexes (KDI_2a_–see Equation (1)) and multiplication (KDI_2b_–see Equation (2)) methods were used [40]. All the numerical values are given in Table S2.

KDI_2a_ = I_MW_ + I_log P_ + I_HD_+ I_HA_ + I_RB_ + I_PSA_ (1)

KDI_2b_ = I_MW_ × I_log P_ × I_HD_ × I_HA_ × I_RB_ × I_PSA_ (2)

The theoretical maximum of KDI_2a_ is 6 and the ligands range from 3.41 to 4.81; the average of 4.08 (±1.27) for clinically used drugs. The theoretical maximum of 1 can be reached for KDI_2b_ and the ligands have values from 0.02 to 0.22, with an average of 0.18 (±0.20) for small molecule pharmaceuticals. The most active compound **33a** has good KDI_a/b_ values of 4.48 and 0.12, respectively, indicating good biocompatibility.

## 3. Materials and Methods

### 3.1. Chemistry Section

General Information. Reagents and solvents were purchased from commercial suppliers (Acros (Waltham, MA, USA) and Sigma-Aldrich (St. Louis, MO, USA)). GC-MS: Agilent 7890A gas chromatograph with quartz column HP-5MS of length 30 m, internal diameter 0.25 mm and stationary phase film thickness 0.25 µm; a quadrupole mass spectrometer Agilent 5975C was used as a detector. ^1^H and ^13^C NMR: Bruker DRX-500 apparatus at 500.13 MHz (^1^H) and 125.76 MHz (^13^C) and Bruker Avance–III 600 apparatus at 600.30 MHz (^1^H) and 150.95 MHz (^13^C), *J* in Hz. HR-MS: DFS Thermo Scientific (Waltham, MA, USA) spectrometer in a full scan mode (15–500 *m*/*z*, 70 eV electron impact ionization, direct sample administration). Optical rotation was measured with polAAr 3005 spectrometer.

All product yields are given for pure compounds purified by recrystallization or isolated by column chromatography (SiO_2_; 60–200 μ; Macherey-Nagel, Düren, Germany). All of the target compounds have a purity of no less than 95% (GC-MS data).

*Synthesis of coumarins* **26**–**31**.

Syntheses were carried out from 2,6-dihydroxyacetophenone and appropriate phenylacetic acids (**14**–**19**) in accordance with article [37]. A mixture of the appropriate phenylacetic acid (18.6 mmol) and 2,6-dihydroxyacetophenone (19.5 mmol) in the presence of triethylamine (56.0 mmol) in acetic anhydride (15 mL) was refluxed for 3 h. After the completion of the reaction, water was added and the mixture was extracted with dichloromethane. The organic phase was separated, washed with saturated sodium bicarbonate, brine and water, dried over anhydrous Na_2_SO_4_, filtered, and evaporated to give the crude product. The products were purified by recrystallization from methanol and dichloromethane.

3-(4-Fluorophenyl)-4-methyl-2-oxo-2*H*-chromen-5-yl acetate **20**. The yield–56%.

3-(4-Bromophenyl)-4-methyl-2-oxo-2*H*-chromen-5-yl acetate **21**. The yield–50%.

3-(4-Methoxyphenyl)-4-methyl-2-oxo-2*H*-chromen-5-yl acetate **22**. The yield–41%.

3-(2-Bromophenyl)-4-methyl-2-oxo-2*H*-chromen-5-yl acetate **23**. The yield–50%.

3-(2-Methoxyphenyl)-4-methyl-2-oxo-2*H*-chromen-5-yl acetate **24**. The yield–42%.

3-(2-Nitrophenyl)-4-methyl-2-oxo-2*H*-chromen-5-yl acetate **25**. The yield–43%. HRMS: 339.0737 [M]^+^; calcd. 339.0735 (C_18_H_13_O_6_N)^+^. ^1^H-NMR (CDCl_3_, *δ* ppm, *J*, Hz): 2.29 (s, 3H, CH_3_CO-18), 2.33 (s, 3H, CH_3_-16), 6.99 (dd, 1H, *J*_7,8_ = 8.4, *J*_7,9_ = 1.0, H-7), 7.31 (m, 2H, H-9, H-15), 7.52 (t, 1H, *J*_8,7_ = *J*_8,9_ = 8.0, H-8), 7.59 (t, 1H, *J*_13,14_ = *J*_13,12_ = 8.3, H-13), 7.70 (t, 1H, *J*_14,15_ = *J*_14,13_ = *J* = 7.5, H-14), 8.22 (d, 1H, *J*_12,13_ = 8.2, H-12). ^13^C NMR (CDCl_3_, *δ* ppm): 148.33 (s, C-1), 158.81 (s, C-2), 126.49 (s, C-3), 147.75 (s, C-4), 113.91 (s, C-5), 153.53 (s, C-6), 115.25 (d, C-7), 131.05 (d, C-8), 119.87 (d, C-9), 129.91 (s, C-10), 145.44 (s, C-11), 125.10 (d, C-12), 129.69 (d, C-13), 133.72 (d, C-14), 132.34 (d. C-15), 21.29 (q, C-16), 168.96 (s, C-17), 20.14 (q, C-18).

A mixture of the appropriate 5-acetyloxy-coumarin **20**–**25** (4.5 mmol) and hydrazine monohydrate (22.5 mmol) in methanol (40 mL) was stirred at 40 °C for 2 h. After the completion of the reaction, water was added and the mixture was then extracted with ethyl acetate. The organic phase was separated, washed with brine and water, dried over anhydrous Na_2_SO_4_, filtered, and evaporated to give the crude product. The products were triturated with methanol. 

3-(4-Fluorophenyl)-5-hydroxy-4-methyl-2*H*-chromen-2-one **26**. The yield–96%.

3-(4-Bromophenyl)-5-hydroxy-4-methyl-2*H*-chromen-2-one **27.** The yield–95%.

5-Hydroxy-3-(4-methoxyphenyl)-4-methyl-2*H*-chromen-2-one **28**. The yield–89%.

3-(2-Bromophenyl)-5-hydroxy-4-methyl-2*H*-chromen-2-one **29**. The yield–93%.

5-Hydroxy-3-(2-methoxyphenyl)-4-methyl-2*H*-chromen-2-one **30**. The yield–83%.

5-Hydroxy-4-methyl-3-(2-nitrophenyl)-2*H*-chromen-2-one **31**. The yield–58%. M.p. 270 °C. HRMS: 296.0559 [M-H]^+^; calcd. 296.0564 (C_16_H_10_O_5_N)^+ 1^H-NMR (DMSO-d6, *δ* ppm, *J*, Hz): 2.41 (s, 3H, CH_3_-16), 6.86 (m, 2H, H-9, H-15), 7.43 (t, 1H, *J*_8,7_ = *J*_8,9_ = 8.0, H-8), 7.56 (d, 1H, *J*_7,8_ = 8.4, *J*_7,9_ = 1.0, H-7), 7.71 (t, 1H, *J*_13,14_ = *J*_13,12_ = 8.3, H-13), 7.85 (t, 1H, *J*_14,15_ = *J*_14,13_ = *J* = 7.5, H-14), 8.19 (d, 1H, *J*_12,13_ = 8.2, H-12), 10.84 (br. s, 1H, OH). ^13^C NMR (DMSO-d6, *δ* ppm): 153.64 (s, C-1), 158.86 (s, C-2), 122.18 (s, C-3), 149.42 (s, C-4), 108.89 (s, C-5), 157.16 (s, C-6), 107.22 (d, C-7), 132.21 (d, C-8), 111.86 (d, C-9), 130.01 (s, C-10), 148.64 (s, C-11), 124.67 (d, C-12), 129.87 (d, C-13), 134.12 (d, C-14), 133.16 (d. C-15), 21.37 (q, C-16).

The ^1^H NMR spectra of compounds **20**–**24** and **26**–**30** synthesized according to [37,38] correspond to those published earlier.

*Synthesis of bromides* **32a**–**d**.

Bromide **32a** was synthesized from geraniol via the reaction with PBr_3_ [30].

PBr_3_ (8.9 mmol) was added to cooled (0–5 °C) solution of geraniol (26.7 mmol) in dry ether (30 mL) and the reaction mixture was stirred for 2 h at r.t. Saturated aqueous NaHCO_3_ was added and the product was extracted with ether. The extracts were washed with brine, dried with Na_2_SO_4_ and evaporated.

Other used bromides **32b**,**c** were synthesized as described above. Compounds **32a**–**c** (the yields 91%, 65%, and 55%, respectively) were sufficiently pure and used for the next step without purification.

Bromide **32d** was synthesized from (-)-nopol via reaction with NBS–PPh_3_ as described in [39].

Triphenylphosphine (2.0 equiv, 6.1 g, 23 mmol) was dissolved in dry DCM (23 mL) under argon. To this solution cooled by ice-water bath, *N*-bromosuccinimide (NBS) (2.0 equiv, 4.2 g, 23 mmol) was added in small portions over 5 min. Later the resulting mixture was stirred at r.t. for 30 min. Pyridine (1 mL) was added and then (-)-nopol (1.0 equiv, 2.0 mL, 12 mmol) was added dropwise over 10 min. The reaction mixture was stirred overnight at r.t. The mixture was diluted with hexane (40 mL) and filtered through a silica gel plug, diluted with EtOAc-hexane (1:1, 40 mL) and filtered through the silica gel plug. The residue was concentrated in vacuo and purified by chromatography on SiO_2_ (hexane) to obtain bromide **32d** (2.3 g, 70% yield).

*Synthesis of compounds* **33**–**38**.

General procedure. DBU (1.0 mmol) and corresponding bromides **32a**–**d** (0.75 mmol) were added to compound **26**–**31** (0.5 mmol) in dry DMF (5 mL) at r.t. under stirring. The reaction mixture was stirred at r.t. for 15 min, and then heated at 60 °C for 5 h. H_2_O (15 mL) was added and the product was extracted with ethyl acetate. The combined extracts were washed with brine, dried with Na_2_SO_4_ and evaporated. The products **33**–**38** were isolated in the individual form (**a**) by recrystallization from ethanol; or (**b**) by column chromatography on silica gel, eluent-hexane, solution containing from 25 to 100% ethyl acetate in hexane.

*(E)-5-(3,7-Dimethylocta-2,6-dienyloxy)-3-(4-fluorophenyl)-4-methyl-2H-chromen-2-one* **33a** Yield 71%, method **b**. M.p. 83 °C. HRMS: 406.1938 [M]^+^; calcd. 406.1939 (C_26_H_27_O_3_F)^+^∙^1^H NMR (CDCl_3_, *δ* ppm, *J*, Hz): 1.57 (br. s, 3H, CH_3_-25), 1.63 (s, 3H, CH_3_-24), 1.72 (br. s, 3H, CH_3_-26), 2.02–2.14 (m, 4H, 2H-20, 2H-21), 2.43 (s, 3H, CH_3_-16), 4.61 (d, 2H, *J*_17,18_ = 6.5, 2H-17), 5.06 (tm, 1H, *J*_22,21_ = 6.5, other *J* < 2, H-22), 5.47 (tm, 1H, *J*_18,17_ = 6.5, other *J* < 2, H-18), 6.75 (dd, 1H, *J*_7,8_ = 8.9, *J*_7,9_ = 2.5, H-7), 6.95 (d, 1H, *J*_9,8_ = 8.3, H-9), 7.11 (t, 2H, *J*_12,11_ = *J*_14,15_ = 8.7, H-12, H-14), 7.23 (dd, 2H, *J*_11,12_ = *J*_15,14_ = 8.7, *J*_11(15),F_ = 5.4, H-11, H-15), 7.39 (t, 1H, *J*_8,7_ = *J*_8,9_ = 8.3, H-8). ^13^C NMR (*δ* ppm, CDCl_3_): 154.11 (s, C-1), 160.68 (s, C-2), 125.27 (s, C-3), 150.21 (s, C-4), 111.15 (s, C-5), 157.61 (s, C-6), 107.32 (d, C-7), 131.27 (d, C-8), 109.47 (d, C-9), 130.90 (s, ^4^*J* = 3.5, C-10), 130.89 (d, ^3^*J* = 8.2, C-11, C-15), 115.35 (d, ^2^*J* = 21.4, C-12, C-14), 162.26 (s, ^1^*J* = 247.1, C-13), 21.92 (q, C-16), 65.83 (t, C-17), 118.52 (d, C-18), 141.85 (s, C-19), 39.28 (t, C-20), 26.06 (t, C-21), 123.40 (d, C-22), 131.80 (s, C-23), 25.53 (q, C-24), 17.57 (q, C-25), 16.51 (q, C-26).

*5-(3,7-Dimethyloctyloxy)-3-(4-fluorophenyl)-4-methyl-2H-chromen-2-one* **33b** Yield 57%, method **b**. M.p. 114 °C. HRMS: 410.2254 [M]^+^; calcd. 410.2252 (C_26_H_31_O_4_F)^+^∙^1^H-NMR (CDCl_3_, *δ* ppm, *J*, Hz): 0.84 (d, 6H, *J*_25,23_ = *J*_24,23_ = 6.6, CH_3_-25, CH_3_-24), 0.93 (d, 3H, *J*_26,19_ = 6.5, CH_3_-26), 1.07–1.35 (m, 6H, 2H-20, 2H-21, 2H-22), 1.44–1.55 (m, 1H, H-23), 1.56–1.71 (m, 2H, H-18, H-19), 1.84–1.91 (m, 1H, H-18′), 2.45 (s, 3H, CH_3_-16), 4.04–4.11 (m, 2H, 2H-17), 6.75 (d, 1H, *J*_7,8_ = 8.3, H-7), 6.95 (dd, 1H, *J*_9,8_ = 8.3, *J*_9,7_ = 0.8, H-9), 7.09–7.14 (br.d, 2H, *J*_12,11_ = *J*_14,15_ = 8.7, H-12, H-14), 7.21–7.26 (m, 2H, H-11, H-15). 7.34 (t, *J*_8,7_ = *J*_8,9_ = 8.3, H-8). ^13^C NMR (*δ* ppm, CDCl_3_): 154.14 (s, C-1), 160.65 (s, C-2), 125.29 (s, C-3), 150.05 (s, C-4), 110.92 (s, C-5), 157.83 (s, C-6), 106.86 and 109.47 (d, C-7, C-9), 131.35 (d, C-8), 130.88 (s, ^4^*J* = 3.47, C-10), 131.89 (d, ^3^*J* = 8.2, C-11, C-15), 115.38 (d, ^2^*J* = 21.6, C-12, C-14), 162.27 (d, ^1^*J* = 247.1, C-13), 22.03 (q, C-16), 67.46 (t, C-17), 36.03 (t, C-18), 29.80 (d, C-19), 37.11 (t, C-20), 24.51 (t, C-21), 39.05 (t, C-22), 27.80 (d, C-23), 22.53 and 22.43 (q, C-24, C-25), 19.44 (q, C-26).

*5-(((1R,5S)-6,6-Dimethylbicyclo[3.1.1]hept-2-en-2-yl)methoxy)-3-(4-fluorophenyl)-4-methyl-2H-chromen-2-one* **33c** Yield 74%, method **b**. M.p. 152 °C. ${\left[\alpha \right]}_{589}^{26.5}$ = −8.30 (*c* = 0.53, CHCl_3_). HRMS: 404.1786 [M]^+^; calcd. 404.1782 (C_26_H_25_O_3_F)^+^∙^1^H NMR (CDCl_3_, *δ* ppm, *J*, Hz): 0.82 (s, 3H, CH_3_-25), 1.16 (d, 1H, *J*_24a,24s_ = 8.7, H-24a), 1.27 (s, 3H, CH_3_-25), 2.09–2.14 (m, 1H, H-21), 2.23–2.27 (m, 1H, H-23), 2.30 (dm, 1H, *J*_20,20_^,^ = 18.0, other *J* < 3.5, H-20), 2.33 (dm, 1H, *J*_20′,20_ = 18.0, other *J* < 3.5, H-20′), 2.38–2.43 (m, 1H, H-24s), 2.41 (s, 3H, CH_3_-16), 4.41–4.48 (m, 2H, 2H-17), 5.63–5.67 (m, 1H, H-19), 6.76 (dd, 1H, *J*_7,8_ = 8.3, *J*_7,9_ = 2.5, H-7), 6.95 (d, 1H, *J*_9,8_ = 8.3, H-9), 7.09–7.15 (m, 2H, *J*_12,11_ = *J*_14,15_ = 8.7, *J*_12(14),F_ = 8.7, H-12, H-14), 7.20–7.25 (m, 2H, *J*_11,12_ = *J*_15,14_ = 8.7, *J*_11(15),F_ = 5.6, H-11, H-15), 7.38 (t, 1H, *J*_8,7_ = *J*_8,9_ = 8.3, H-8). ^13^C NMR (*δ* ppm, CDCl_3_): 154.13 (s, C-1), 160.65 (s, C-2), 125.24 (s, C-3), 150.10 (s, C-4), 111.00 (s, C-5), 157.68 (s, C-6), 107.19 (d, C-7), 131.26 (d, C-8), 109.56 (d, C-9), 130.89 (s, ^4^*J* = 3.5, C-10), 131.87 (d, ^3^*J* = 8.2, C-11, C-15), 115.41 (d, ^2^*J* = 21.6, C-12, C-14), 162.27 (s, 1*J* = 247.1, C-13), 22.04 (q, C-16), 72.08 (t, C-17), 143.03 (s, C-18), 122.25 (d, C-19), 31.17 (t, C-20), 40.54 (d, C-21), 37.91 (s, C-22), 43.53 (d, C-23), 31.44 (t, C-24), 26.04 (k, C-25), 20.96 (k, C-26).

*5-(2-((1R,5S)-6,6-Dimethylbicyclo[3.1.1]hept-2-en-2-yl)ethoxy)-3-(4-fluorophenyl)-4-methyl-2H-chromen-2-one* **33d** Yield 46%, method **b**. M.p. 114 °C. ${\left[\alpha \right]}_{589}^{24.9}$ = −11.10 (*c* = 0.36, CHCl_3_). HRMS: 417.1859 [M-H]^+^; calcd. 417.1861 (C_27_H_26_O_3_F)^+^∙^1^H NMR (CDCl_3_, *δ* ppm, *J*, Hz): 0.80 (c, 3H, CH_3_-27), 1.13 (d, 1H, *J*_25a,25s_ = 8.6, H-25a), 1.26 (s, 3H, CH_3_-26), 2.03–2.11 (m, 2H, C-24, C-22), 2.18–2.25 (m, 2H, H-21), 2.36 (ddd, 1H, *J*_25s,25a_ = 8.6, *J*_25s,22_ = *J*_25s,24_ = 5.6, H-25s), 2.45 (s, 3H, CH_3_-16), 2.46–2.51 (m, 2H, H-18), 4.06 (t, *J*_17,18_ = 6.6, 2H, 2H-17), 5.30–5.34 (m, 1H, H-20), 6.73 (dd, 1H, *J*_7,8_ = 8.3, *J*_7,9_ = 2.5, H-7), 6.95 (d, 1H, *J*_9,8_ = 8.3, H-9), 7.09–7.15 (m, 2H, *J*_12,11_ = *J*_14,15_ = 8.7, *J*_12(14),F_ = 8.7, H-12, H-14), 7.20–7.26 (m, 2H, *J*_11,12_ = *J*_15,14_ = 8.7, *J*_11(15),F_ = 5.6, H-11, H-15), 7.40 (t, 1H, *J*_8,7_ = *J*_8,9_ = 8.3, H-8).^13^C NMR (*δ* ppm, CDCl_3_): 154.09 (s, C-1), 160.70 (s, C-2), 125.23 (s, C-3), 150.20 (s, C-4), 110.91 (s, C-5), 157.68 (s, C-6), 106.79 (d, C-7), 131.34 (d, C-8), 109.53 (d, C-9), 130.86 (s, ^4^*J* = 3.5, C-10), 131.88 (d, ^3^*J* = 8.2, C-11, C-15), 115.41 (d, ^2^*J* = 21.7, C-12, C-14), 162.25 (s, ^1^*J* = 247.1, C-13), 22.04 (q, C-16), 67.10 (t, C-17), 36.34 (t, C-18), 143.78 (s, C-19), 118.81(d, C-20), 31.23 (t, C-21), 40.56 (d, C-22), 37.95 (s, C-23), 45.52 (d, C-24), 31.49 (t, C-25), 26.13 (q, C-26), 20.98 (q, C-27).

*(E)-3-(4-Bromophenyl)-5-(3,7-dimethylocta-2,6-dienyloxy)-4-methyl-2H-chromen-2-one* **34a** Yield 89%, method **b**. M.p. 93 °C. HRMS: 466.1140 [M]^+^; calcd. 466.1138 (C_26_H_27_O_3_Br)^+^∙ ^1^H-NMR (CDCl_3_, *δ* ppm, *J*, Hz): 1.57 (br.s, 3H, CH_3_-25), 1.63 (s, 3H, CH_3_-24), 1.72 (br.s, 3H, CH_3_-26), 2.01–2.16 (m, 4H, 2H-20, 2H-21), 2.44 (s, 3H, CH_3_-16), 4.61 (d, 2H, *J*_17,18_ = 6.6, 2H-17), 5.04 (tm, 1H, *J*_22,21_6.7, other *J* ≤ 1.5, H-22), 5.44–5.49 (m, 1H, H-18), 6.75 (dd, 1H, *J*_7,8_ = 8.3, *J*_7,9_ = 2.5, H-7), 6.95 (d, 1H, *J*_9,8_ = 8.3, H-9), 7.14 (br.d, 2H, *J*_11,12_ =*J*_15,14_ = 8.4, H-11, H-15). 7.40 (t, *J*_8,7_ = *J*_8,9_ = 8.3, H-8), 7.55 (br.d, 2H, *J*_12,11_ = *J*_14,15_ = 8.4, H-12, H-14). ^13^C NMR (*δ* ppm, CDCl_3_): 154.09 (s, C-1), 160.46 (s, C-2), 125.07 (s, C-3), 150.28 (s, C-4), 111.03 (s, C-5), 157.61 (c, C-6), 107.31 and 109.45 (d, C-7, C-9), 131.40 (d, C-8), 133.95 (s, C-10), 131.85 (d, C-11, C-15), 131.53 (d, C-12, C-14), 122.03 (s, C-13), 21.97 (q, C-16), 65.81 (t, C-17), 118.46 (d, C-18), 141.86 (c, C-19), 39.28 (t, C-20), 26.04 (t, C-21), 123.37 (d, C-22), 131.04 (c, C-23), 25.56 (q, C-24), 17.59 (q, C-25), 16.53(q, C-26).

*3-(4-Bromophenyl)-5-(3,7-dimethyloctyloxy)-4-methyl-2H-chromen-2-one* **34b** Yield 50%, method **a**. M.p. 137 °C. HRMS: 470.1457 [M]^+^; calcd. 470.1451 (C_26_H_31_O_3_Br)^+ 1^H-NMR (CDCl_3_, *δ* ppm, *J*, Hz): 0.84 (d, 6H, *J*_25,23_ = *J*_24,23_ = 6.6, CH_3_-25, CH_3_-24), 0.93 (d, 3H, *J*_26,19_ = 6.5, CH_3_-26), 1.07–1.35 (m, 6H, 2H-20, 2H-21, 2H-22), 1.44–1.55 (m, 1H, H-23), 1.55–1.70 (m, 2H, H-18, H-19), 1.83–1.92 (m, 1H, H-18′), 2.45 (s, 3H, CH_3_-16), 4.03–4.11 (m, 2H, 2H-17), 6.75 (d, 1H, *J*_7,8_ = 8.3, H-7), 6.95 (dd, 1H, *J*_9,8_ = 8.3, *J*_9,7_ = 0.8, H-9), 7.14 (br.d, 2H, *J*_11,12_ =*J*_15,14_ = 8.3, H-11, H-15). 7.40 (t, *J*_8,7_ = *J*_8,9_ = 8.3, H-8), 7.55 (br.d, 2H, *J*_12,11_ = *J*_14,15_ = 8.3, H-12, H-14). ^13^C NMR (*δ* ppm, CDCl_3_): 154.16 (s, C-1), 160.39 (s, C-2), 125.15 (s, C-3), 150.06 (s, C-4), 110.86 (s, C-5), 157.87 (s, C-6), 106.90 and 109.49 (d, C-7, C-9), 131.47 (d, C-8), 133.97 (s, C-10), 131.85 (d, C-11, C-15), 131.57 (d, C-12, C-14), 122.07 (s, C-13), 22.05 (q, C-16), 67.49 (t, C-17), 36.03 (t, C-18), 29.83 (d, C-19), 37.11 (t, C-20), 24.52 (t, C-21), 39.06 (t, C-22), 27.81 (d, C-23), 22.55 and 22.44 (q, C-24, C-25), 19.45 (q, C-26).

*3-(4-Bromophenyl)-5-(((1R,5S)-6,6-dimethylbicyclo[3.1.1]hept-2-en-2-yl)methoxy)-4-methyl-2H-chromen-2-one* **34c** Yield 80%, method **b**. M.p. 148 °C. ${\left[\alpha \right]}_{589}^{26.5}$ = −6.30 (*c* = 0.54, CHCl_3_). HRMS: 464.0978 [M-H]^+^; calcd. 464.0982 (C_26_H_25_O_3_Br)^+^∙^1^H NMR (CDCl_3_, *δ* ppm, *J*, Hz): 0.82 (s, 3H, CH_3_-26), 1.16 (d, 1H, *J*_24a,24s_ = 8.7, H-24a), 1.27 (s, 3H, CH_3_-25), 2.09–2.14 (m, 1H, H-21), 2.25 (ddd, 1H, *J*_23,21_ = *J*_23,24s_ = 5.6, *J*_23,19_ = 1.4, H-23), 2.27–2.29 (m, 1H, H-20), 2.29–2.32 (m, 1H, H-20′), 2.40 (ddd, 1H, *J*_24s,24a_ = 8.7, *J*_24s,21_= *J*_24s,23_ = 5.6, H-24s), 2.41 (s, 1H, CH_3_-16), 4.42–4.45 (m, 2H, 2H-17), 5.63–5.66 (m, 1H, H-19), 6.75 (dd, 1H, *J*_7,8_ = 8.9, *J*_7,9_ = 2.5, H-7), 6.95 (dd, 1H, *J*_9,8_ =8.3, *J*_9,7_ = 1.0, H-9), 7.13 (br.d, 2H, *J*_11,12_ = *J*_15,14_ = 8.4, H-11, H-15), 7.39 (t, 1H, *J*_8,7_= *J*_8,9_ = 8.3, H-8), 7.56 (br.d, 2H, *J*_12,11_ = *J*_14,15_ = 8.4, H-12, H-14). ^13^C NMR (*δ* ppm, CDCl_3_): 154.13 (s, C-1), 160.39 (s, C-2), 125.06 (s, C-3), 150.14 (s, C-4), 110.90 (s, C-5), 157.71 (s, C-6), 107.20 (d, C-7), 131.39 (d, C-8), 109.56 (d, C-9), 133.97 (s, C-10), 131.84 (d, C-11, C-15), 131.59 (d, C-12, C-14), 122.05 (s, C-13), 22.07 (q, C-16), 72.09 (t, C-17), 142.91 (s, C-18), 122.91 (d, C-19), 31.16 (t, C-20), 40.51 (d, C-21), 37.90 (s, C-22), 43.50 (d, C-23), 31.43 (t, C-24), 26.04 (q, C-25), 20.96 (q, C-26).

*(E)-5-(3,7-Dimethylocta-2,6-dienyloxy)-3-(4-methoxyphenyl)-4-methyl-2H-chromen-2-one* **35a** Yield 63%, method **b**. M.p. 85 °C. HRMS: 418.2143 [M]^+^; calcd. 418.2139 (C_27_H_30_O_4_)^+ 1^H-NMR (CDCl_3_, *δ* ppm, *J*, Hz): 1.57 (br.s, 3H, CH_3_-25), 1.63 (s, 3H, CH_3_-24), 1.72 (br.s, 3H, CH_3_-26), 2.02–2.14 (m, 4H, 2H-20, 2H-21), 2.46 (s, 3H, CH_3_-27), 3.82 (s, 3H, CH_3_-16), 4.61 (d, 2H, *J*_17,18_ = 6.5, 2H-17), 5.04 (tm, 1H, *J*_22,21_ = 6.7, other *J* < 2.0, H-22), 5.48 (tm, 1H, *J*_18,17_ = 6.6, other *J* < 2.0, H-18), 6.74 (dd, 1H, *J*_7,8_ = 8.3, *J*_7,9_ = 1.0, H-7), 6.93–6.97 (m, 3H, H-9, H-12, H-14), 7.19 (br.d, 2H, *J*_11,12_ =*J*_15,14_ = 8.7, H-11, H-15), 7.37 (t, *J*_8,7_ = *J*_8,9_ = 8.3, H-8). ^13^C NMR (*δ* ppm, CDCl_3_): 154.03 (s, C-1), 160.98 (s, C-2), 125.95 (s, C-3), 149.63 (s, C-4), 111.39 (s, C-5), 157.51 (s, C-6), 109.44 and 107.21 (d, C-7, C-9), 130.92 (s, C-8), 127.17 (s, C-10), 131.29 (d, C-11, C-15), 113.77 (d, C-12, C-14), 159.05 (s, C-13), 55.15 (q, C-16), 65.79 (t, C-17), 118.62 (d, C-18), 141.69 (s, C-19), 39.28 (t, C-20), 26.07 (t, C-21), 123.42 (d, C-22), 131.79 (s, C-23), 25.54 (q, C-24), 17.58 (q, C-25), 16.51 (q, C-26), 21.96 (q, C-27).

*5-(3,7-Dimethyloctyloxy)-3-(4-methoxyphenyl)-4-methyl-2H-chromen-2-one***35b** Yield 88%, method **b**. M.p. 97 °C. HRMS: 422.2456 [M]^+^; calcd. 422.2452 (C_27_H_34_O_4_)^+^∙^1^H-NMR (CDCl_3_, *δ* ppm, *J*, Hz): 0.84 (d, 6H, *J*_26,24_ = *J*_26,24_ = 6.6, CH_3_-26, CH_3_-25), 0.93 (d, 3H, *J*_27,20_ = 6.5, CH_3_-27), 1.07–1.19 (m, 3H, 2H-23, H-21), 1.19–1.35 (m, 3H, H-21′, 2H-22), 1.44–1.55 (m, 1H, H-24), 1.55–1.72 (m, 2H, H-19, H-20), 1.84–1.91 (m, 1H, H-19′), 2.47 (s, 3H, CH_3_-17), 3.83 (s, 3H, OCH_3_-16), 4.03–4.11 (m, 2H, 2H-18), 6.74 (d, 1H, *J*_7,8_ = 8.3, H-7), 6.94 (d, 1H, *J*_9,8_ = 8.3, H-9), 6.96 (br.d, 2H, *J*_12,11_ = *J*_14,15_ = 8.4, H-12, H-14) 7.19 (br.d, 2H, *J*_11,12_ =*J*_15,14_ = 8.3, H-11, H-15). 7.37 (t, *J*_8,7_ = *J*_8,9_ = 8.3, H-8). ^13^C NMR (*δ* ppm, CDCl_3_): 154.05 (s, C-1), 160.95 (s, C-2), 125.96 (s, C-3), 149.48 (s, C-4), 111.15 (s, C-5), 157.73 (s, C-6), 106.75 and 109.43 (d, C-7, C-9), 131.00 (d, C-8), 127.15 (s, C-10), 131.29 (d, C-11, C-15), 113.80 (d, C-12, C-14), 159.08 (s, C-13), 55.15 (q, C-16), 22.07 (q, C-17), 67.39 (t, C-18), 36.04 (t, C-19), 29.79 (d, C-20), 37.11 (t, C-21), 24.51 (t, C-22), 39.05 (t, C-23), 27.80 (d, C-24), 22.54 and 22.43 (q, C-25, C-26), 19.44 (q, C-27).

*5-(((1R,5S)-6,6-Dimethylbicyclo[3.1.1]hept-2-en-2-yl)methoxy)-3-(4-methoxyphenyl)-4-methyl-2H-chromen-2-one* **35c** Yield 87%, method **b**. ${\left[\alpha \right]}_{589}^{26.5}$ = −7.76 (*c* = 0.49, CHCl_3_) HRMS: 416.1979 [M]^+^; calcd. 416.1982 (C_27_H_28_O_4_)^+^∙^1^H NMR (CDCl_3_, *δ* ppm, *J*, Hz): 0.82 (s, 3H, CH_3_-27), 1.16 (d, 1H, *J*_25a,25s_ = 8.7, H-25a), 1.27 (s, 3H, CH_3_-26), 2.08–2.13 (m, 1H, H-22), 2.26 (ddd, 1H, *J*_24,22_ = *J*_24,25s_ = 5.6, *J*_24,20_ = 1.4, H-24), 2.27–2.29 (m, 1H, H-21), 2.29–2.32 (m, 1H, H-21′), 2.40 (ddd, 1H, *J*_25s,25a_ = 8.7, *J*_25s,22_= *J*_25s,24_ = 5.6, H-25s), 2.43 (s, 1H, CH_3_-17), 3.83 (s, 3H, CH_3_-16), 4.42–4.45 (m, 2H, 2H-18), 5.63–5.66 (m, 1H, H-20), 6.74 (dd, 1H, *J*_7,8_ = 8.3, *J*_7,9_ = 2.5, H-7), 6.94 (dd, 1H, *J*_9,8_ =8.3, *J*_9,7_ = 1.0, H-9), 6.96 (d, 2H, *J*_12,11_ = *J*_14,15_ = 8.7, H-12, H-14), 7.16 (br.d, 2H, *J*_11,12_ = *J*_15,14_ = 8.7, H-11, H-15), 7.36 (t, 1H, *J*_8,7_ = *J*_8,9_ = 8.3, H-8), ^13^C NMR (*δ* ppm, CDCl_3_): 154.06 (s, C-1), 160.96 (s, C-2), 125.91 (s, C-3), 149.56 (s, C-4), 111.23 (s, C-5), 157.59 (s, C-6), 107.06 (d, C-7), 130.93 (d, C-8), 109.54 (d, C-9), 127.17 (s, C-10), 131.27 (d, C-11, C-15), 113.83 (d, C-12, C-14), 159.07 (s, C-13), 55.15 (q, C-16), 22.09 (q, C-17), 72.03 (t, C-18), 143.10 (s, C-19), 122.14 (d, C-20), 31.16 (t, C-21), 40.53 (d, C-22), 37.90 (s, C-23), 43.51 (d, C-24), 31.44 (t, C-25), 26.04 (q, C-26), 20.95 (q, C-27).

*(E)-3-(2-Bromophenyl)-5-(3,7-dimethylocta-2,6-dienyloxy)-4-methyl-2H-chromen-2-one* **36a** Yield 55%, method **b**. HRMS: 466.1140 [M]^+^; calcd. 466.1138 (C_26_H_27_O_3_Br)^+^∙^1^H-NMR (CDCl_3_, *δ* ppm, *J*, Hz): 1.56 (br.s, 3H, CH_3_-25), 1.63 (s, 3H, CH_3_-24), 1.72 (br.s, 3H, CH_3_-26), 2.00–2.14 (m, 4H, 2H-20, 2H-21), 2.36 (s, 3H, CH_3_-16), 4.61 (d, 2H, *J*_17,18_ = 6.5, 2H-17), 5.04 (tm, 1H, *J*_22,21_ = 6.7, other *J* ≤ 1.5, H-22), 5.45–5.49 (tm, 1H, *J*_18,17_ = 6.5, other *J* ≤ 1.5, H-18), 6.76 (dd, 1H, *J*_7,8_ = 8.3, *J*_7,9_ = 0.9, H-7), 6.97 (dd, 1H, *J*_9,8_ = 8.3, *J*_9,7_ = 0.9, H-9), 7.20–7.27 (m, 2H, H-13, H-15). 7.37 (td, 1H, *J*_14,15_ = *J*_14,13_ = 7.5, *J*_14,12_ = 1.2, H-14), 7.41 (t, *J*_8,7_ = *J*_8,9_ = 8.3, H-8), 7.66 (dd, 2H, *J*_12,13_ = 8.0, *J*_12,14_ = 1.2, H-12). ^13^C NMR (*δ* ppm, CDCl_3_): 154.45 (s, C-1), 159.58 (s, C-2), 125.70 (s, C-3), 151.34 (s, C-4), 110.76 (s, C-5), 157.78 (c, C-6), 107.24 and 109.60 (d, C-7, C-9), 131.42 (d, C-8), 124.52 (s, C-10), 136.24 (s, C-11), 132.74 (d, C-12), 129.51 (d, C-13), 127.54 (d, C-14), 131,44 (d, C-15), 21.31 (q, C-16), 65.83 (t, C-17), 118.50 (d, C-18), 141.84 (c, C-19), 39.28 (t, C-20), 26.07 (t, C-21), 123.40 (d, C-22), 131.79 (c, C-23), 25.52 (q, C-24), 17.57 (q, C-25), 16.53 (q, C-26).

*3-(2-Bromophenyl)-5-(3,7-dimethyloctyloxy)-4-methyl-2H-chromen-2-one* **36b** Yield 84%, method **a**. M.p. 73 °C. HRMS: 470.1446 [M]^+^; calcd. 470.1451 (C_26_H_31_O_3_Br)^+^∙^1^H-NMR (CDCl_3_, *δ* ppm, *J*, Hz): 0.83 (d, 6H, *J*_25,23_ = *J*_24,23_ = 6.6, CH_3_-25, CH_3_-24), 0.92 (d, 3H, *J*_26,19_ = 6.5, CH_3_-26), 1.07–1.34 (m, 6H, 2H-20, 2H-21, 2H-22), 1.43–1.54 (m, 1H, H-23), 1.55–1.70 (m, 2H, H-18, H-19), 1.82–1.93 (m, 1H, H-18′), 2.38 (s, 3H, CH_3_-16), 4.04–4.11 (m, 2H, 2H-17), 6.76 (d, 1H, *J*_7,8_ = 8.3, H-7), 6.98 (dd, 1H, *J*_9,8_ = 8.3, *J*_9,7_ = 0.8, H-9), 7.20–7.27 (m, 2H, H-13, H-15). 7.38 (td, 1H, *J*_14,15_ = *J*_14,13_ = 7.5, *J*_14,12_ = 1.2, H-14), 7.42 (t, *J*_8,7_ = *J*_8,9_ = 8.3, H-8), 7.67 (dd, 2H, *J*_12,13_ = 8.0, *J*_12,14_ = 1.2, H-12). ^13^C NMR (*δ* ppm, CDCl_3_): 154.43 (s, C-1), 159.61 (s, C-2), 125.65 (s, C-3), 151.03 (s, C-4), 110.49 (s, C-5), 157.97 (c, C-6), 106.79 and 109.55 (d, C-7, C-9), 131.41 (d, C-8), 124.50 (s, C-10), 136.18 (s, C-11), 132.75 (d, C-12), 129.56 (d, C-13), 127.58 (d, C-14), 131.54 (d, C-15), 21.48 (q, C-16), 67.40 (t, C-17), 36.00 (t, C-18), 29.76 (d, C-19), 37.08 (t, C-20), 24.50 (t, C-21), 39.02 (t, C-22), 27.79 (d, C-23), 22.55 and 22.43 (q, C-24, C-25), 19.43 (q, C-26).

*3-(2-Bromophenyl)-5-(((1R,5S)-6,6-dimethylbicyclo[3.1.1]hept-2-en-2-yl)methoxy)-4-methyl-2H-chromen-2-one* **36c** Yield 57%, method **b**. M.p. 157 °C. ${\left[\alpha \right]}_{589}^{26.5}$ = −0.66 (*c* = 0.91, CHCl_3_). HRMS: 464.0976 [M-H]^+^; calcd. 464.0982 (C_27_H_27_O_3_)^+^∙^1^H NMR (CDCl_3_, *δ* ppm, *J*, Hz): 0.81(0.82) (c, 3H, CH_3_-26), 1.16(1.15) (d, 1H, *J*_24a,24s_ = 8.6, H-24a), 1.26 (s, 3H, CH_3_-25), 2.08–2.13 (m, 1H, H-21), 2.23–2.29 (m, 2H, H-20, H-23), 2.29–2.32 (m, 1H, H-20′), 2.34 (s, 1H, CH_3_-16), 2.37–2.42 (ddd, 1H, *J*_24s,24a_ = 8.6, *J*_24s,21_= *J*_24s,23_ = 5.6, H-24s), 4.40–4.48 (m, 2H, 2H-17), 5.63–5.67 (m, 1H, H-19), 6.77 (d, 1H, *J*_7,8_ = 8.3, H-7), 6.97 (d, 1H, *J*_9,8_ =8.3, H-9), 7.20–7.26 (m, 2H, H-13, H-15), 7.38–7.40 (m, 1H, *J*_14,13_ = *J*_14,15_ = 8.1, *J*_14,12_= 7.5, H-14), 7.40 (t, 1H, *J*_8,7_ = *J*_8,9_ = 8.3, H-8), 7.67 (d, 1H, *J*_12,13_ = 8.2, H-12). ^13^C NMR (*δ* ppm, CDCl_3_): 154.46 (s, C-1), 159.57 (s, C-2), 125.68 (s, C-3), 151.01 (s, C-4), 110.65 (s, C-5), 157.85(157.82) (s, C-6), 107.20(107.18) (d, C-7), 131.44 (d, C-8), 109.67 (d, C-9), 124.54 (s, C-10), 136.23 (s, C-11), 132.78 (d, C-12), 129.54 (d, C-13), 127.59 (d, C-14), 131.43 (d, C-15), 21.47 (q, C-16), 72.11(72.07) (t, C-17), 143.02(143.00) (s, C-18), 122.32(122.28) (d, C-19), 31.17(31.16) (t, C-20), 40.53 (d, C-21), 37.90(37.89) (s, C-22), 43.52 (d, C-23), 31.44(31.43) (t, C-24), 26.03 (q, C-25), 20.96(20.95) (q, C-26).

Signals of two conformers were observed in the ^1^H and ^13^C NMR spectra of compounds **36c**, **37c**, **37d** and **38c**.

*(E)-5-(3,7-Dimethylocta-2,6-dienyloxy)-3-(2-methoxyphenyl)-4-methyl-2H-chromen-2-one* **37a** Yield 60%, method **b**. M.p. 99 °C. HRMS: 418.2127 [M]^+^; calcd. 418.2139 (C_27_H_30_O_4_)^+^∙^1^H-NMR (CDCl_3_, *δ* ppm, *J*, Hz): 1.57 (br. s, 3H, CH_3_-25), 1.63 (s, 3H, CH_3_-24), 1.72 (br. s, 3H, CH_3_-26), 2.00–2.13 (m, 4H, 2H-20, 2H-21), 2.38 (s, 3H, CH_3_-27), 3.76 (s, 3H, CH_3_-16), 4.60 (d, 2H, *J*_17,18_ = 6.5, 2H-17), 5.04 (tm, 1H, *J*_22,21_ = 6.7, other *J* < 2.0, H-22), 5.47 (tm, 1H, *J*_18,17_ = 6.5, other *J* < 2.0, H-18), 6.74 (dd, 1H, *J*_7,8_ = 8.3, *J*_7,9_ = 0.8, H-7), 6.95 (dd, 1H, *J*_9,8_ = 8.3, *J*_9,7_ = 1.3, H-9), 6.96 (d, 1H, *J*_12,13_ = 6.4, H-12), 7.01 (td, *J*_14,15_ = *J*_14,13_ = 7,4, *J*_14,12_ =1.0, H-14), 7.13 (dd, 1H, *J*_15,14_ = 7.4, *J*_15,13_ = 1.2, H-15), 7.33–7.37 (m, 1H, H-13), 7.36 (t, *J*_8,7_ = *J*_8,9_ = 8.3, H-8). ^13^C NMR (*δ* ppm, CDCl_3_): 154.33 (s, C-1), 160.27 (s, C-2), 123.12 (s, C-3), 150.49 (s, C-4), 111.28 (s, C-5), 157.53 (s, C-6), 109.50 (d, C-7), 130.83 (d, C-8), 107.11 (d, C-9), 124.04 (s, C-10), 157.13 (s, C-11), 111.05 (d, C-12), 129.47 (d, C-13), 120.55 (d, C-14), 131.22 (d, C-15), 55.48 (q, C-16), 65.77 (t, C-17), 118.71 (d, C-18), 141.57 (s, C-19), 39.28 (t, C-20), 26.08 (t, C-21), 123.43 (d, C-22), 131.77 (s, C-23), 25.52 (q, C-24), 17.57 (q, C-25), 16.51 (q, C-26), 21.44 (q, C-27).

*5-(3,7-Dimethyloctyloxy)-3-(2-methoxyphenyl)-4-methyl-2H-chromen-2-one* **37b** Yield 73%, method **b**. M.p. 114 °C. HRMS: 422.2448 [M]^+^; calcd. 422.2452 (C_27_H_34_O_4_)^+^∙^1^H-NMR (CDCl_3_, *δ* ppm, *J*, Hz): 0.83 (d, 6H, *J*_25,23_ = *J*_24,23_ = 6.6, CH_3_-25, CH_3_-24), 0.92 (d, 3H, *J*_26,19_ = 6.5, CH_3_-26), 1.08–1.35 (m, 6H, 2H-20, 2H-21, 2H-22), 1.43–1.55 (m, 1H, H-23), 1.55–1.72 (m, 2H, H-18, H-19), 1.81–1.93 (m, 1H, H-18′), 2.40 (s, 3H, CH_3_-27), 3.76 (s, 3H, CH_3_-16), 4.03–4.10 (m, 2H, 2H-17), 6.74 (d, 1H, *J*_7,8_ = 8.3, H-7), 6.96 (dd, 1H, *J*_9,8_ = 8.3, *J*_9,7_ = 0.8, H-9), 6.98 (d, 1H, *J*_12,13_ = 6.4, H-12), 7.01 (td, *J*_14,15_ = *J*_14,13_ = 7.4, *J*_14,12_ =1.0, H-14), 7.14 (dd, 1H, *J*_15,14_ = 7.4, *J*_15,13_ = 1.2, H-15), 7.33–7.38 (m, 1H, H-13), 7.37 (t, *J*_8,7_ = *J*_8,9_ = 8.3, H-8). ^13^C NMR (*δ* ppm, CDCl_3_): 154.30 (s, C-1), 160.26 (s, C-2), 123.09 (s, C-3), 150.37 (s, C-4), 111.04 (s, C-5), 157.74 (s, C-6), 109.47 (d, C-7), 130.93 (d, C-8), 106.60 (d, C-9), 123.98 (s, C-10), 157.11 (s, C-11), 111.04 (d, C-12), 129.50 (d, C-13), 120.56 (d, C-14), 131.21 (d, C-15), 55.48 (q, C-16), 67.30 (t, C-17), 36.04 (t, C-18), 29.75 (d, C-19), 37.09 (t, C-20), 24.51 (t, C-21), 39.03 (t, C-22), 27.79 (d, C-23), 22.54 and 22.43 (q, C-24, C-25), 19.42 (q, C-26), 21.60 (q, C-27).

*5-(((1R,5S)-6,6-Dimethylbicyclo[3.1.1]hept-2-en-2-yl)methoxy)-3-(2-methoxyphenyl)-4-methyl-2H-chromen-2-one* **37c** Yield 56%, method **b**. M.p. 164 °C. ${\left[\alpha \right]}_{589}^{26.5}$ = −2.92 (*c* = 0.48, CHCl_3_). HRMS: 416.1986 [M]^+^; calcd. 416.1982 (C_27_H_28_O_4_)^+^∙^1^H NMR (CDCl_3_, *δ* ppm, *J*, Hz): 0.81 (s, 3H, CH_3_-27), 1.16 (d, 1H, *J*_25a,25s_ = 8.7, H-25a), 1.26 (s, 3H, CH_3_-26), 2.07–2.12 (m, 1H, H-22), 2.25 (ddd, 1H, *J*_24,22_ = *J*_24,25s_ = 5.6, *J*_24,20_ = 1.4, H-24), 2.26–2.28 (m, 1H, H-21), 2.29–2.32 (m, 1H, H-21′), 2.35 (s, 3H, CH_3_-17), 2.36–2.41 (m, 1H, H-25s), 3.76 (s, 3H, CH_3_-16), 4.39–4.46 (m, 2H, 2H-18), 5.62–5.65 (m, 1H, H-20), 6.73 (dd, 1H, *J*_7,6_ = 8.4, *J*_7,9_ = 1.3, H-7), 6.94 (dd, 1H, *J*_9,8_ =8.3, *J*_9,7_ = 1.0, H-9), 6.97 (d, 1H, *J*_12,11_ = 8.3, H-12), 6.99–7.03 (m, 1H, H-14), 7.11–7.14 (m, 1H, H-15), 7.33–7.37 (m, 2H, H-8, H-13). ^13^C NMR (*δ* ppm, CDCl_3_): 154.30 (s, C-1), 160.24 (s, C-2), 123.06(123.03) (s, C-3), 150.40(150.38) (s, C-4), 11.14(111.12) (s, C-5), 157.60(157.58) (s, C-6), 106.96(106.94) (d, C-7), 130.84(130.83) (d, C-8), 109.55 (d, C-9), 124.03(124.02) (s, C-10), 157.12 (s, C-11), 111.08(111.06) (d, C-12), 129.48 (d, C-13), 120.57(120.56) (d, C-14), 131.21 (d, C-15), 55.48 (q, C-16), 21.61 (q, C-17), 72.00 (t, C-18), 143.15(143.14) (s, C-19), 122.04(122.02) (d, C-20), 31.14 (t, C-21), 40.51 (d, C-22), 37.87 (s, C-23), 43.49(43.48) (d, C-24), 31.42 (t, C-25), 26.01 (q, C-26), 20.93(20.91) (q, C-27).

5*-(2-((1R,5S)-6,6-Dimethylbicyclo[3.1.1]hept-2-en-2-yl)ethoxy)-3-(2-methoxyphenyl)-4-methyl-2H-chromen-2-one* **37d** Yield 44%, method **b**. M.p. 135 °C. ${\left[\alpha \right]}_{589}^{26.5}$ = −21.20 (*c* = 0.50, CHCl_3_). HRMS: 429.2062 [M-H]^+^; calcd. 429.2060 (C_29_H_29_O_5_)^+^∙^1^H NMR (CDCl_3_, *δ* ppm, *J*, Hz): 0.80 (c, 3H, CH_3_-28), 1.14 (d, 1H, *J*_26a,26s_ = 8.6, H-26a), 1.26 (s, 3H, CH_3_-27), 2.03–2.11 (m, 2H, C-25, C-23), 2.18–2.24 (m, 2H, H-22), 2.36 (ddd, 1H, *J*_26s,26a_ = 8.6, *J*_26s,23_ = *J*_26s,25_ = 5.6, H-26s), 2.39 (s, 3H, CH_3_-17), 2.46–2.51 (m, 2H, H-19), 3.76 (s, 3H, CH_3_-17), 4.02–4.09 (m, 2H, 2H-18), 5.30–5.34 (m, 1H, H-21), 6.72 (dd, 1H, *J*_7,8_ = 8.4, *J*_7,9_ = 1.0, H-7), 6.95 (dd, 1H, *J*_9,8_ = 8.3, *J*_9,7_ = 1.0, H-9), 6.97 (d, 1H, *J*_12,13_ = 8.4, H-12), 7.02 (td, 1H, *J*_14,15_ = *J*_14.13_ = 7.5, H-14), 7.14 (dd, 1H, *J*_15,14_ = 7.4, *J*_15,13_ = 1.7, H-15), 7.34–7.39 (m, 2H, H-8, H-13). ^13^C NMR (*δ* ppm, CDCl_3_): 154.35 (s, C-1), 160.25 (s, C-2), 123.09 (s, C-3), 150.44 (s, C-4), 110.12 (s, C-5), 157.64 (s, C-6), 106.61 (d, C-7), 130.88 (d, C-8), 109.55 (d, C-9), 124.07 (s, C-10), 157.16 (s, C-11), 111.10 (d, C-12), 129.49 (d, C-13), 120.58 (d, C-14), 131.27 (d, C-15), 55.50 (q, C-16), 21.56(21.55) (q, C-17), 67.10(67.08) (t, C-18), 36.38 (t, C-19), 143.88 (s, C-20), 118.75(118.74) (d, C-21), 31.24 (t, C-22), 40.62 (d, C-23), 37.96 (s, C-24), 45.62(45.59) (d, C-25), 31.50(31.49) (t, C-26), 26.15 (q, C-27), 21.00(20.98) (q, C-28).

*(E)-5-(3,7-Dimethylocta-2,6-dienyloxy)-4-methyl-3-(2-nitrophenyl)-2H-chromen-2-one* **38a** Yield 61%, method **b**. M.p. 73 °C. HRMS: 433.1886 [M]^+^; calcd. 433.1884 (C_26_H_27_O_5_N)^+^∙^1^H-NMR (CDCl_3_, *δ* ppm, *J*, Hz): 1.56 (br. s, 3H, CH_3_-25), 1.62 (s, 3H, CH_3_-24), 1.72 (br. s, 3H, CH_3_-26), 2.01–2.14 (m, 4H, 2H-20, 2H-21), 2.39 (s, 3H, CH_3_-27), 4.61 (d, 2H, *J*_17,18_ = 6.5, 2H-17), 5.03 (tm, 1H, *J*_22,21_ = 6.7, other *J* < 2.0, H-22), 5.46 (tm, 1H, *J*_18,17_ = 6.5, other *J* < 2.0, H-18), 6.77 (dd, 1H, *J*_7,8_ = 8.2, *J*_7,9_ = 0.8, H-7), 6.97 (d, 1H, *J*_9,8_ = 8.2, H-9), 7.32 (dd, 1H, *J*_15,14_ = 7.3, *J*_15,13_ = 1.2, H-15), 7.41 (t, *J*_8,7_ = *J*_8,9_ = 8.2, H-8), 7.56 (t, 1H, *J*_13,14_ = *J*_13,12_ = 8.2, H-13), 7.68 (t, 1H, *J*_14,15_ = *J*_14,13_= *J* = 7.3, H-14), 8.17 (d, 1H, *J*_12,13_ = 8.2, H-12). ^13^C NMR (*δ* ppm, CDCl_3_): 154.19 (s, C-1), 159.53 (s, C-2), 123.63 (s, C-3), 149.25 (s, C-4), 110.78 (s, C-5), 157.66 (s, C-6), 109.59 (d, C-7), 131.54 (d, C-8), 107.41 (d, C-9), 130.77 (s, C-10), 148.74 (s, C-11), 124.88 (d, C-12), 129.19 (d, C-13), 133.44 (d, C-14), 132.64 (d, C-15), 21.73 (q, C-16), 65.84 (t, C-17), 118.44 (d, C-18), 141.91 (s, C-19), 39.27 (t, C-20), 26.06 (t, C-21), 123.40 (d, C-22), 131.78 (s, C-23), 25.51 (q, C-24), 17.56 (q, C-25), 16.52 (q, C-26).

*5-(3,7-Dimethyloctyloxy)-4-methyl-3-(2-nitrophenyl)-2H-chromen-2-one* **38b** Yield 75%, method **b**. M.p. 61 °C. HRMS: 437.2191 [M]^+^; calcd. 437.2197 (C_26_H_31_O_5_N)^+^∙^1^H-NMR (CDCl_3_, *δ* ppm, *J*, Hz): 0.82 (d, 6H, *J*_25,23_ = *J*_24,23_ = 6.6, CH_3_-25, CH_3_-24), 0.92 (d, 3H, *J*_26,19_ = 6.5, CH_3_-26), 1.06–1.33 (m, 6H, 2H-20, 2H-21, 2H-22), 1.42–1.54 (m, 1H, H-23), 1.54–1.69 (m, 2H, H-18, H-19), 1.81–1.92 (m, 1H, H-18′), 2.41 (s, 3H, CH_3_-16), 4.02–4.10 (m, 2H, 2H-17), 6.76 (dd, 1H, *J*_7,8_ = 8.2, *J*_7,9_ = 0.8, H-7), 6.96 (d, 1H, *J*_9,8_ = 8.2, H-9), 7.33 (dd, 1H, *J*_15,14_ = 7.3, *J*_15,13_ = 1.2, H-15), 7.41 (t, *J*_8,7_ = *J*_8,9_ = 8.2, H-8), 7.56 (t, 1H, *J*_13,14_ = *J*_13,12_ = 8.2, H-13), 7.68 (t, 1H, *J*_14,15_ = *J*_14,13_= *J* = 7.3, H-14), 8.18 (d, 1H, *J*_12,13_ = 8.2, H-12). ^13^C NMR (*δ* ppm, CDCl_3_): 154.18 (s, C-1), 159.56 (s, C-2), 124.93 (s, C-3), 149.17 (s, C-4), 110.54 (s, C-5), 157.88 (s, C-6), 106.99 and 109.57 (d, C-7, C-9), 130.74 (d, C-8), 123.61 (s, C-10), 148.70 (s, C-11), 124.93 (d, C-12), 129.26 (d, C-13), 133.53 (d, C-14), 132.65 (d, C-15), 21.96 (q, C-16), 67.46 (t, C-17), 36.00 (t, C-18), 29.77 (d, C-19), 37.11 (t, C-20), 24.52 (t, C-21), 39.05 (t, C-22), 27.81 (d, C-23), 22.57 and 22.46 (q, C-24, C-25), 19.46 (q, C-26).

*5-(((1R,5S)-6,6-Dimethylbicyclo[3.1.1]hept-2-en-2-yl)methoxy)-4-methyl-3-(2-nitrophenyl)-2H-chromen-2-one* **38c.** Yield 65%, method **b**. ${\left[\alpha \right]}_{589}^{26.5}$ = −3.64 (*c* = 0.33, CHCl_3_) HRMS: 431.1723 [M]^+^; calcd. 431.1727 (C_26_H_25_O_5_N)^+^∙^1^H NMR (CDCl_3_, *δ* ppm, *J*, Hz): 0.81(0.82) (s, 3H, CH_3_-26), 1.15 (d, 1H, *J*_24a,24s_ = 8.6, H-24a), 1.26 (s, 3H, CH_3_-25), 2.08–2.12 (m, 1H, H-21), 2.23–2.29 (m, 2H, H-20, H-23), 2.29–2.32 (m, 1H, H-20′), 2.37(2.36) (s, 3H, CH_3_-16), 2.35–2.42 (m, 1H, H-24s), 4.40–4.47 (m, 2H, 2H-17), 5.63–5.66 (m, 1H, H-19), 6.77 (d, 1H, *J*_7,8_ = 8.4, H-7), 6.97 (d, 1H, *J*_9,8_ =8.4, H-9), 7.31–7.35 (tm, 1H, *J*_15,14_ = 7.6, *J*_15,13_ = 1.4, H-15), 7.41 (t, 1H, *J*_8,7_ = *J*_8,9_ = 8.4, H-8), 7.54–7.59 (t, 1H, *J*_13,14_ = 7.9, H-13), 7.67–7.71 (tm, *J*_14,15_ = *J*_14,13_ = 7.5, *J*_14,12_ = 1.3, 1H, H-14), 8.19 (dm, 1H, *J*_12,13_ = 8.3, H-12). ^13^C NMR (*δ* ppm, CDCl_3_): 154.21 (s, C-1), 159.53 (s, C-2), 123.62(123.59) (s, C-3), 149.14 (s, C-4), 110.68 (s, C-5), 157.75(157.73) (s, C-6), 107.31(107.29) (d, C-7), 131.55 (d, C-8), 109.71 (d, C-9), 130.79(130.77) (s, C-10), 148.73(148.72) (s, C-11), 124.95 (d, C-12), 129.22 (d, C-13), 133.51 (d, C-14), 132.63 (d, C-15), 21.91 (q, C-16), 72.12 (t, C-17), 143.01(142.94) (s, C-18), 122.36(122.33) (d, C-19), 31.18(31.16) (t, C-20), 40.52 (d, C-21), 37.92(37.90) (s, C-22), 43.53(43.50) (d, C-23), 31.43 (t, C-24), 26.04(26.02) (q, C-25), 20.97(20.95) (q, C-26).

### 3.2. Biology Section

#### 3.2.1. Real-Time Detection of TDP1 Activity

The biosensor, 16-mer DNA oligonucleotide (5′-[FAM] AAC GTC AGGGTC TTC C [BHQ]-3′) was synthesized in the Laboratory of Nucleic Acid Chemistry at the Institute of Chemical Biology and Fundamental Medicine (Novosibirsk, Russia). Real-time fluorescence detection of TDP1 enzyme activity was carried out as described [53]. The recombinant TDP1 was purified as described [54], using plasmid pET 16B-TDP1, kindly provided by Dr. K.W. Caldecott (University of Sussex, Brighton, UK). To the reaction mixture (200 μL) containing 50 nM oligonucleotide in buffer (50 mM Tris-HCl, pH 8.0, 50 mM NaCl, and 7 mM β-mercaptoethanol), varied concentrations of the tested compounds and purified TDP1 in a final concentration of 1.5 nM were added.

Fluorescence intensity was measured (Ex485/Em520 nm) using POLARstar OPTIMA fluorimeter (BMG LABTECH, GmbH, Ortenberg, Germany) every 1 min for 7 min. The half maximal inhibitory concentrations (IC_50_) were determined using a six-point concentration response curve and calculated using MARS Data Analysis 2.0 (BMG LABTECH). At least three independent experiments were carried out to obtain the IC_50_ values.

#### 3.2.2. Cytotoxicity Assays

The cytotoxicity of the compounds to HeLa (human cervical cancer) and HEK293A (human embryonic kidney) cell lines was determined using standard colorimetric MTT-test [55]. Cells were seeded in 96-well plates (5000 cells per well) and cultured in DMEM medium (Invitrogen, Carlsbad, CA, USA) supplemented with 10% fetal bovine serum (FBS) (Invitrogen), penicillin (100 units/mL), and streptomycin (100 μg/mL) at 37 °C and 5% CO_2_ in a humid atmosphere. Control wells contained 1% DMSO. At 30–50% confluence, the tested compounds were added to the medium. To determine the cytotoxicity of Tdp1 inhibitors, the cells were allowed to attach for 24 h and were treated with compounds with concentrations ranging from 1 to 100 μM for 72 h at 37 °C. All measurements were repeated three times.

To study the effect of the compounds on the cytotoxicity of topotecan, an aqueous solution of topotecan was used at concentrations from 0.2 to 2 μM for HeLa cells and from 0.05 to 0.5 μM for HEK293A cells against the background of 10 μM of the compounds. Cells treated with compounds alone without topotecan were used as controls.

### 3.3. Computer Aided Drug Design

The compounds were modelled using the TDP1 crystal structure (PDB ID: 6W7K, resolution 1.70 Å) [42], obtained from the Protein Data Bank (PDB) [56,57]. The GOLD (v2020.2.0) software was used to prepare the crystal structures for docking, i.e., the hydrogen atoms were added, water molecules deleted and the co-crystallized ligand identified: 4-[(2-phenylimidazo[1,2-*a*]pyridin-3-yl)amino]benzene-1,2-dicarboxylic acid (TG7). The Scigress version FQ 3.4.4 program [58] was used to build the compounds and the MM3 [59,60,61] force field was applied to identify the global minimum using the CONFLEX method [62] followed by energy minimization. The docking center for the TDP1 catalytic pocket was defined as the position of the co-crystallized ligand TG7 with 10 Å radius. Fifty docking runs were allowed for each ligand with default search efficiency (100%). The basic amino acids lysine and arginine were defined as protonated. Furthermore, aspartic and glutamic acids were assumed to be deprotonated. The GoldScore(GS) [44] and ChemScore(CS) [45,46] ChemPLP(Piecewise Linear Potential) [47] and ASP(Astex Statistical Potential) [48] scoring functions were implemented to predict the binding modes and relative binding energies of the ligands using the GOLD v2020.2.0 software suite.

The QikProp 6.2 [63] software package was used to calculate the molecular descriptors for all the ligands. QikProp was benchmarked for the calculated descriptors [64]. The KD were derived from the descriptors as described by Eurtivong and Reynisson [52]. For application in Excel, columns for each property were created and the following equations used do derive the KDI numbers for each descriptor: KDI MW: = EXP(-((*MW*-371.76)^2)/(2*(112.76^2))), KDI Log P: =EXP(-((*LogP*-2.82)^2)/(2*(2.21^2))), KDI HD: =EXP(-((*HD-*1.88)^2)/(2*(1.7^2))), KDI HA: =EXP(-((*HA*-5.72)^2)/(2*(2.86^2))), KDI RB = EXP(-((*RB*-4.44)^2)/(2*(3.55^2))), and KDI PSA: =EXP(-((*PSA*-79.4)^2)/(2*(54.16^2))). These equations can simply be copied into Excel and the descriptor name (e.g., *MW*) substituted with the value in the relevant column. To derive KDI_2A_, this equation was used: =(KDI MW + KDI LogP + KDI HD + KDI HA + KDI RB + KDI PSA) and for KDI_2B_: =(KDI MW * KDI LogP * KDI HD * KDI HA * KDI RB * KDI PSA).

## 4. Conclusions

Compounds combining the 5-hydroxycoumarin and monoterpenoid moieties were synthesized for the first time by the condensation of the coumarin derivatives containing an aryl fragment at position 3 with acyclic and bicyclic monoterpenoid bromides. A study of TDP1 inhibitory activities of the compounds showed that the most potent inhibitors are 5-hydroxycoumarin derivatives bearing an acyclic monoterpene fragment, with IC_50_ up to 130 nM. Such activity makes these compounds the most potent coumarin-based TDP1 inhibitors found so far. These inhibitors generally demonstrated low or no cytotoxicity against cancer HeLa and conditionally normal HEK 293A cell lines. Importantly, the most active TDP1 inhibitor demonstrated a significant synergistic effect with anticancer drug topotecan against the HeLa cancer cell line but not against HEK293A cells. The ability of new inhibitors to occupy the catalytic pocket of TDP1 was confirmed by molecular modeling studies. Based on chemical space analysis, it is assumed that the inhibitors could have good biocompatibility. Thus, we developed a new structural type of potent TDP1 inhibitors which are promising for further pharmacological studies as adjuvant therapy against cancer in combination with Top 1 poisons, such as topotecan.

## Figures and Tables

**Table 1:** TDP1 inhibitory activities of compounds **33**–**38** (IC_50_, μM) ^1^.

Compounds		33	34	35	36	37	38
Terp		R^1^/R^2^ H/F	R^1^/R^2^ H/Br	R^1^/R^2^ H/OMe	R^1^/R^2^ Br/H	R^1^/R^2^ OMe/H	R^1^/R^2^ NO_2_/H
**a**	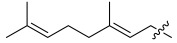	0.13 ± 0.03	1.06 ± 0.06	6.63 ± 1.12	0.85 ± 0.15	1.22 ± 0.04	1.03 ± 0.08
**b**	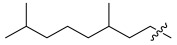	0.29 ± 0.07	- (n/s) ^2^	0.37 ± 0.16	≥50	2.60 ± 0.35	2.18 ± 0.13
**c**	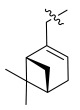	5.25 ± 0.04	0.90 ± 0.07	1.14 ± 0.15	4.36 ± 1.14	0.79 ± 0.06	3.83 ± 0.29
**d**	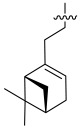	2.51 ± 0.14	-	-	-	3.93 ± 0.77	-

^1^ Furamidine 1.2 ± 0.3 μM. ^2^ Not solved.

**Table 2:** Cytotoxicity of compounds and their influence on the cytotoxic effect of topotecan.

Compound	IC_50_, µM	CC_50_, µM HEK 293A	Tpc CC_50_, µM HEK 293A	CC_50_, µM HeLa	Tpc CC_50_, µM HeLa
**Tpc**			0.04 ± 0.012		0.88 ± 0.24
**33a**	0.13 ± 0.03	>100	0.06 ± 0.02	>100	0.170 ± 0.005
**34a**	1.06 ± 0.06	53 ± 1.2	0.05 ± 0.02	32 ± 3	0.40 ± 0.05
**35a**	7 ± 1	>100	0.02 ± 0.01	>100	0.5 ± 0.09
**36a**	0.9 ± 0.2	59 ± 6	0.044 ± 0.008	49 ± 2	0.61 ± 0.01
**37a**	1.22 ± 0.04	16.0 ± 0.7	0.03 ± 0.02	18 ± 10	0.47 ± 0.05
**38a**	1.03 ± 0.08	>100	0.38 ± 0.008	>100	0.71 ± 0.18
**33b**	0.29 ± 0.07	40 ± 10	-	>100	0.7 ± 0.2
**35b**	0.4 ± 0.2	>100	0.035 ± 0.009	90.7 ± 0.5	0.6 ± 0.2
**33c**	5.25 ± 0.04	>100	0.08 ± 0.06	89 ± 7	0.53 ± 0.01
**34c**	0.9 ± 0.07	>100	0.42 ± 0.03	>100	0.6 ± 0.1
**35c**	1.1 ± 0.2	>100	0.076 ± 0.01	73 ± 6	0.35 ± 0.08
**36c**	4.36 ± 1.14	>100	0.047 ± 0.02	73.5 ± 0.6	0.83 ± 0.2
**37c**	0.79 ± 0.06	52.3 ± 0.9	0.3 ± 0.1	72 ± 2	0.5 ± 0.2
**38c**	3.8 ± 0.3	32 ± 13	0.080 ± 0.008	83 ± 3	0.6 ± 0.3
**37d**	3.9 ± 0.8	87 ± 7	0.06 ± 0.01	48 ± 4	0.8 ± 0.2

## Data Availability

The data presented in this study are available upon request from the corresponding author.

## Supplementary Materials

The following supporting information can be downloaded at: https://www.mdpi.com/article/10.3390/ijms24119155/s1, Cytotoxicity of compounds; NMR ^1^H and ^13^C spectra of compounds **25, 30** and **33**–**38**; additional data for the computer aided drug design.
