# Supplementary material for: New 5-Hydroxycoumarin-Based Tyrosyl-DNA Phosphodiesterase I Inhibitors Sensitize Tumor Cell Line to Topotecan
Source: Int J Mol Sci. 2023 May 23;24(11):9155. doi: 10.3390/ijms24119155 (PMC10253161; doi:10.3390/ijms24119155)
Supplement: Supplementary file 1 [file ijms-24-09155-s001.zip › ijms-2343031-supplementary.pdf]

## SUPPLEMENTARY INFORMATION

Figure S1. Cytotoxicity of compounds NMR $^{1}$H and $^{13}$C spectra of compounds **25**, **30** and **33–38.**

Tables S1 to S3 for the computer aided drug design

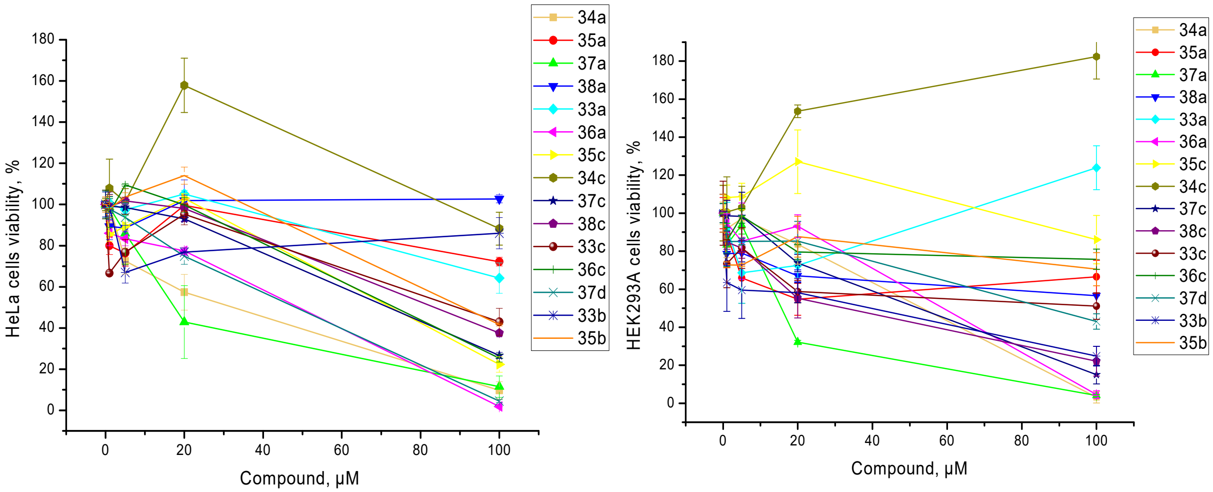


Figure S1. The effect of compounds on the survival of HeLa (**left**) and HEK293A (**right**) cells.

Compound **25**

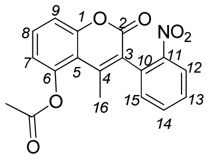

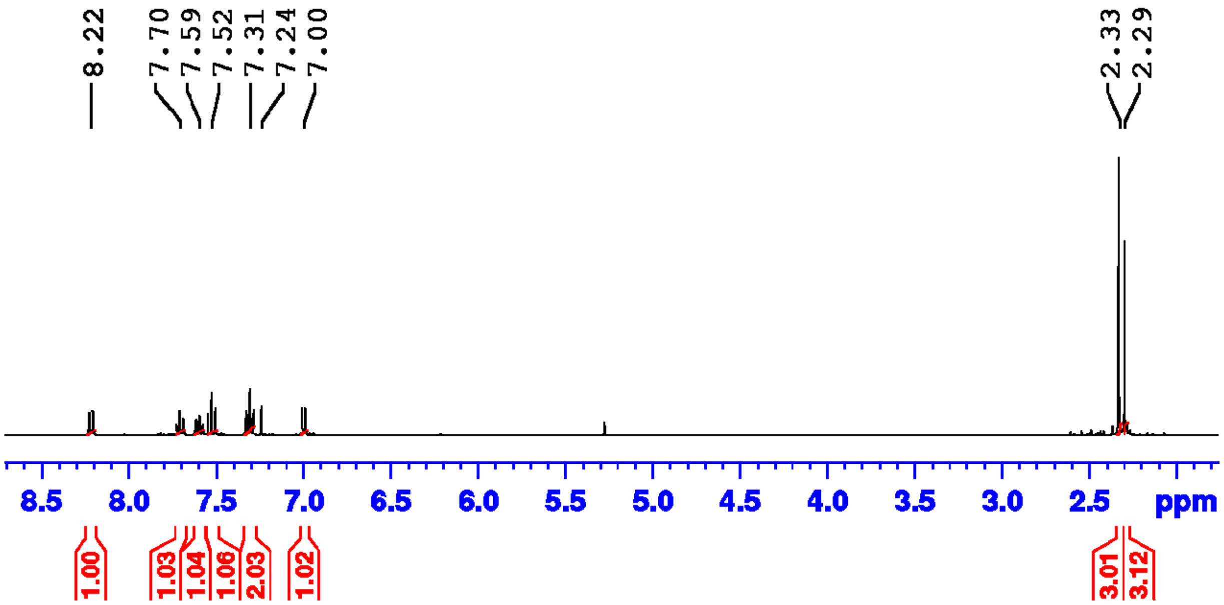
8.22
7.70
7.59
7.52
7.31
7.24
7.00
2.33
2.29
8.5
8.0
7.5
7.0
6.5
6.0
5.5
5.0
4.5
4.0
3.5
3.0
2.5
ppm
1.00
1.03
1.04
1.06
2.03
1.02
3.01
3.12

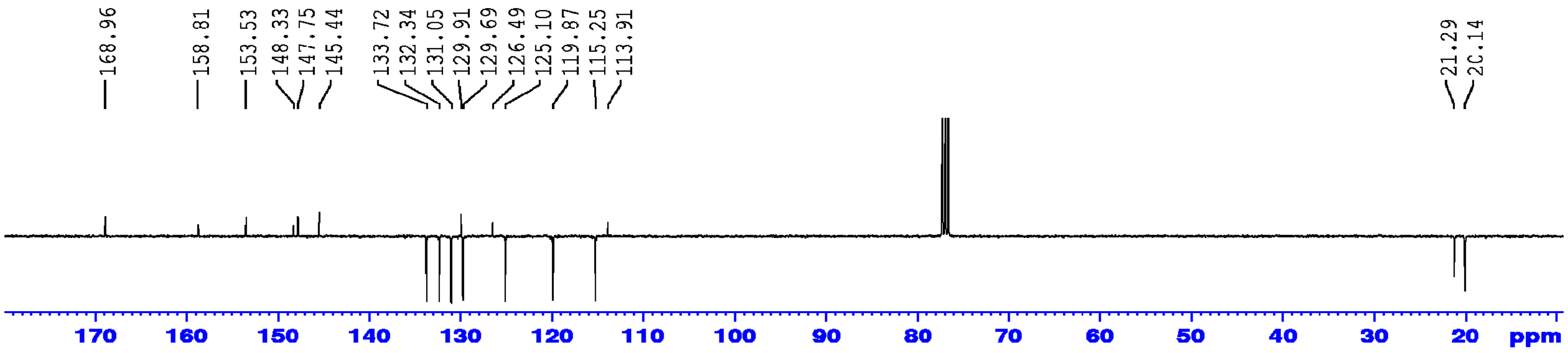
168.96
158.81
153.53
148.33
147.75
145.44
133.72
132.34
131.05
129.91
129.69
126.49
125.10
119.87
115.25
113.91
21.29
20.14
170
160
150
140
130
120
110
100
90
80
70
60
50
40
30
20
ppm

Compound **30**

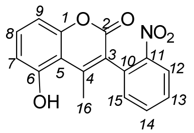
NO2
OH
1 2 3 4 5 6 7 8 9 10 11 12 13 14 15 16

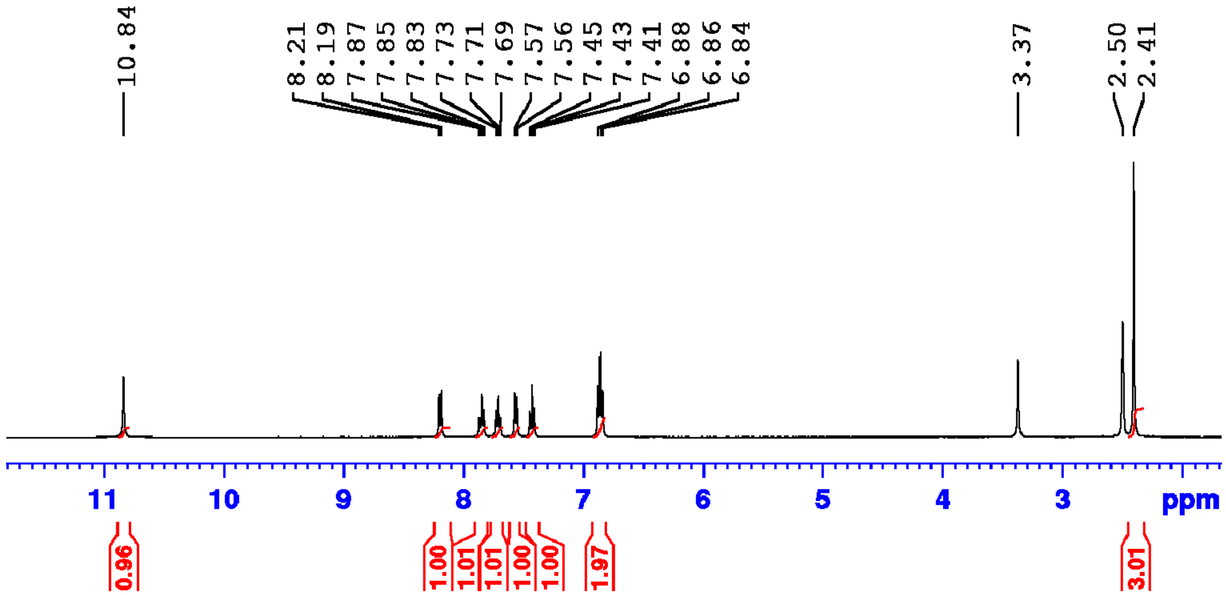
10.84
8.21 8.19 7.87 7.85 7.83 7.73 7.71 7.69 7.57 7.56 7.45 7.43 7.41 6.88 6.86 6.84
3.37
2.50 2.41
11 10 9 8 7 6 5 4 3 ppm
0.96 1.00 1.01 1.01 1.00 1.00 1.97 3.01

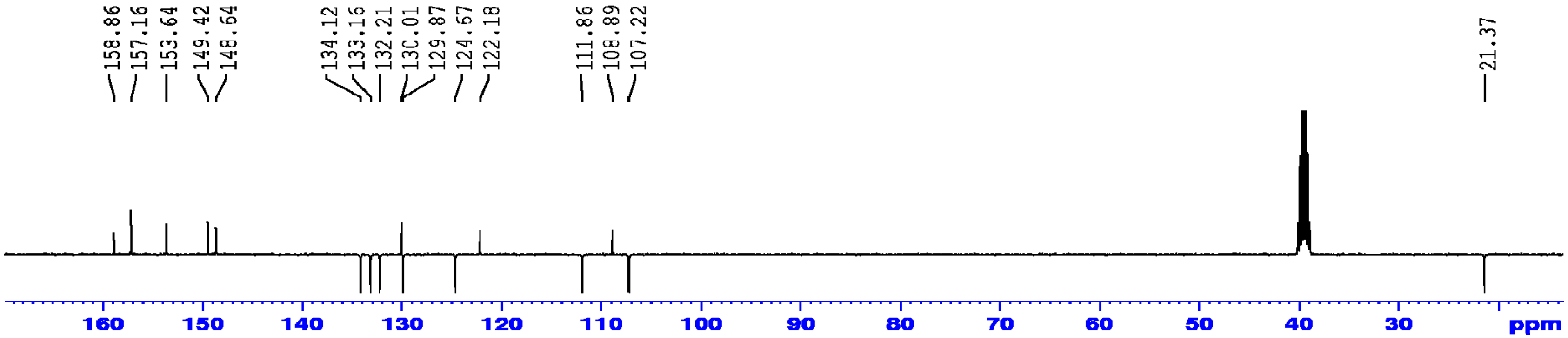
158.86 157.16 153.64 149.42 148.54
134.12 133.16 132.21 130.01 129.87 124.57 122.18
111.86 108.89 107.22
21.37
160 150 140 130 120 110 100 90 80 70 60 50 40 30 ppm

Compound **33a**

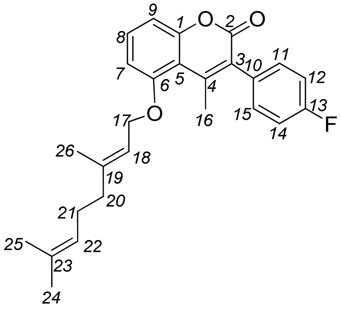


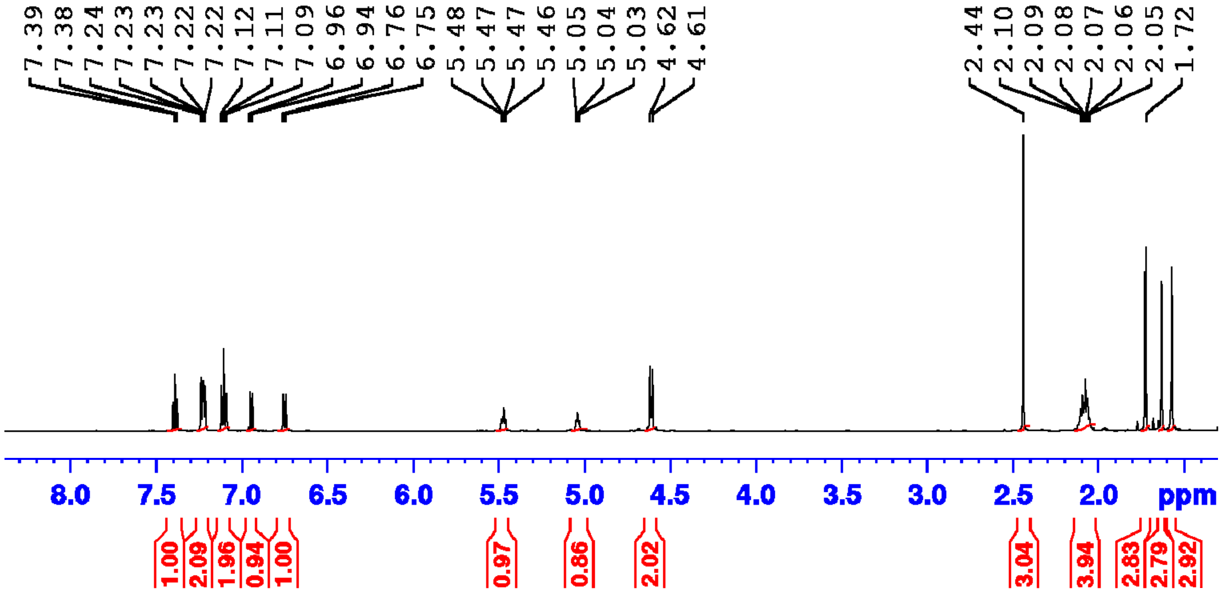


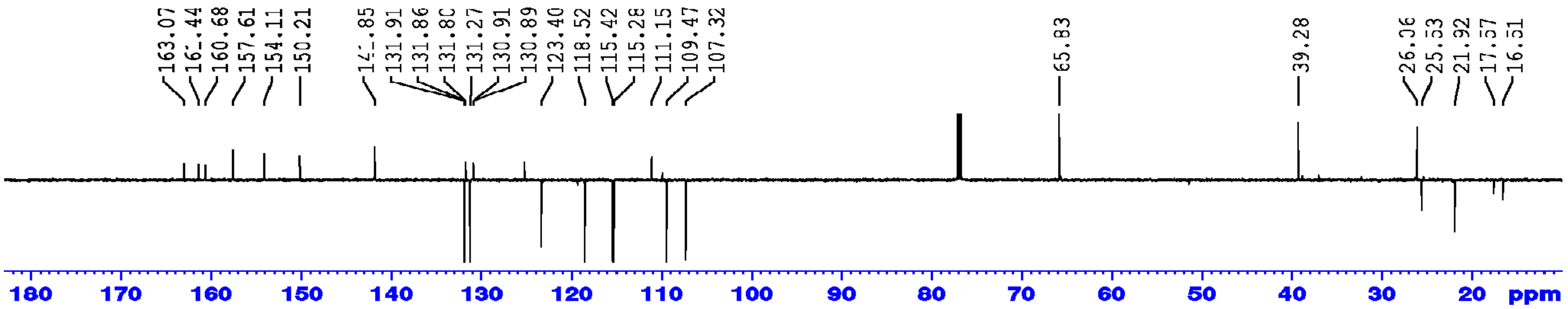

Compound **33b**

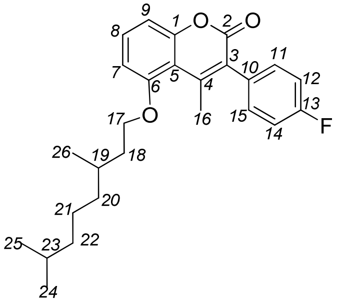


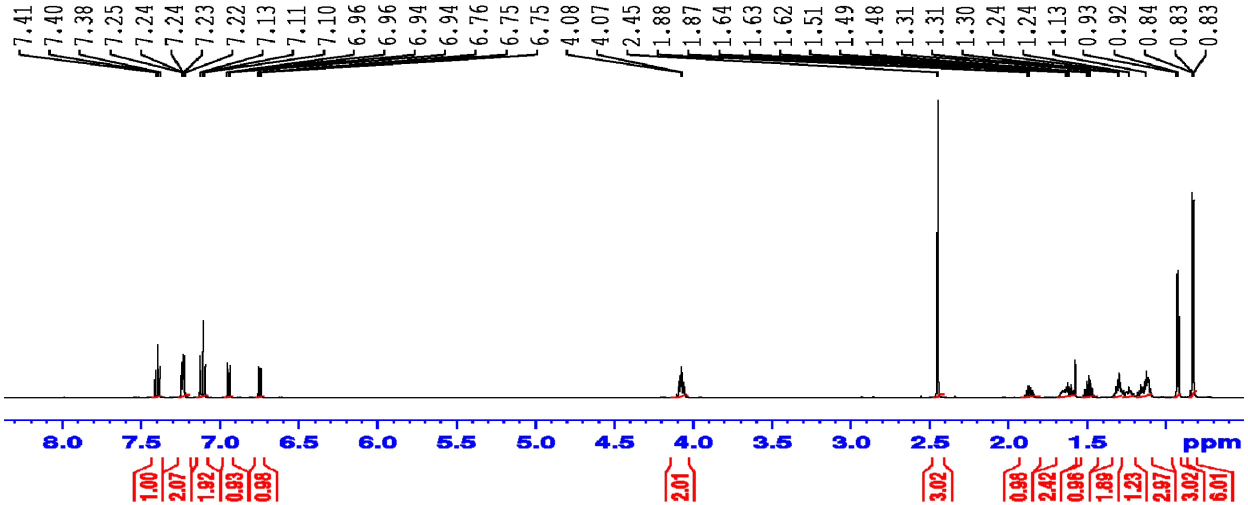


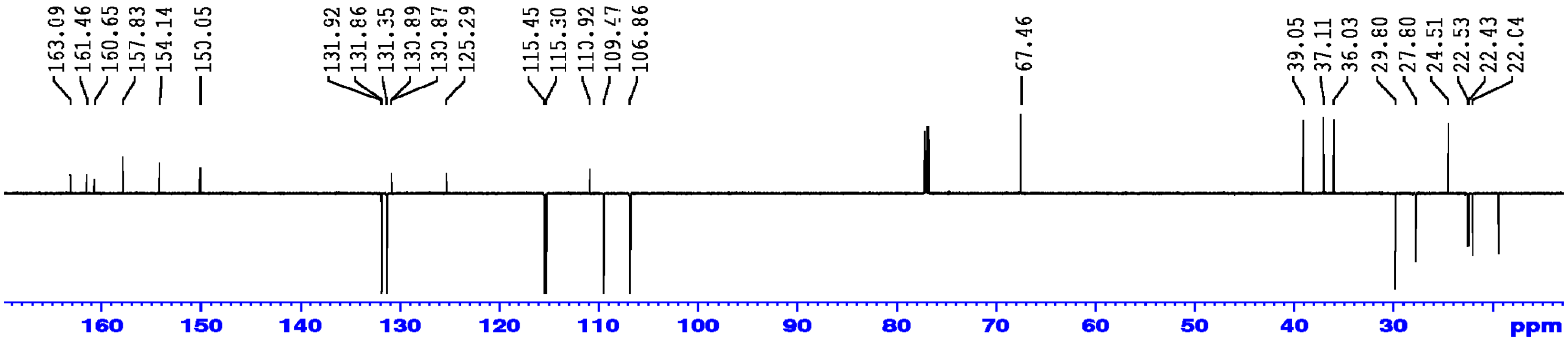

Compound **33c**

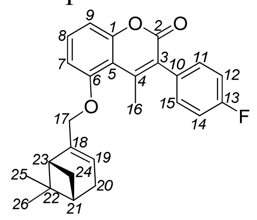


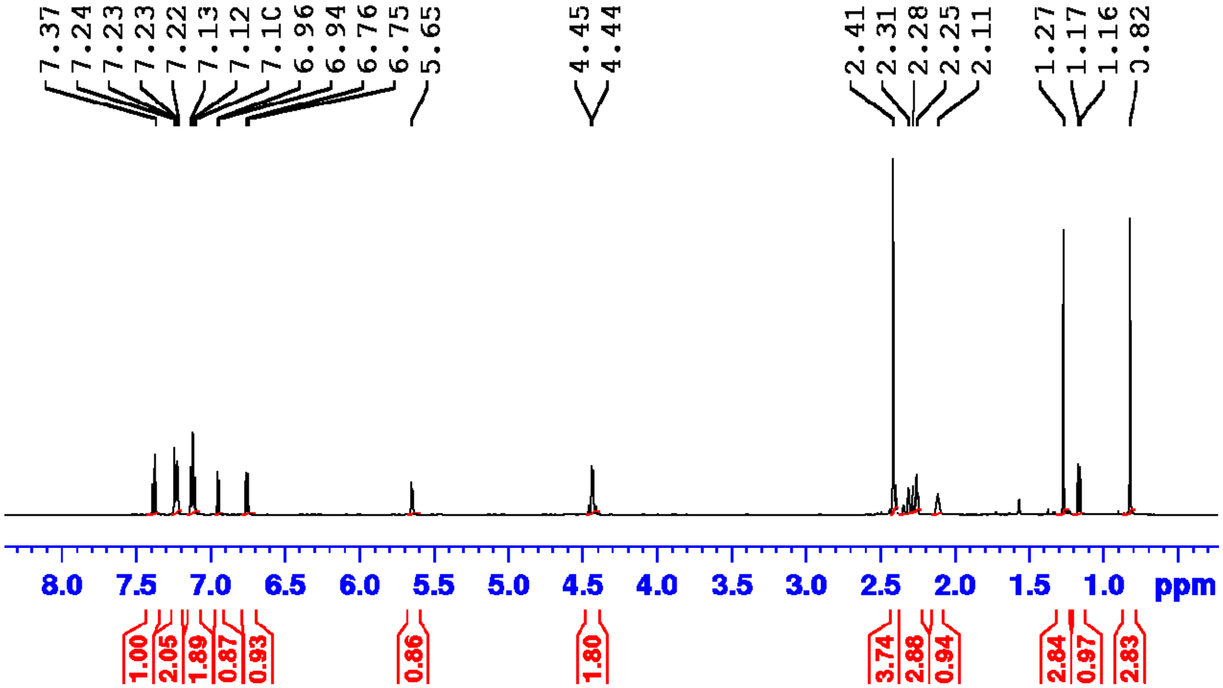


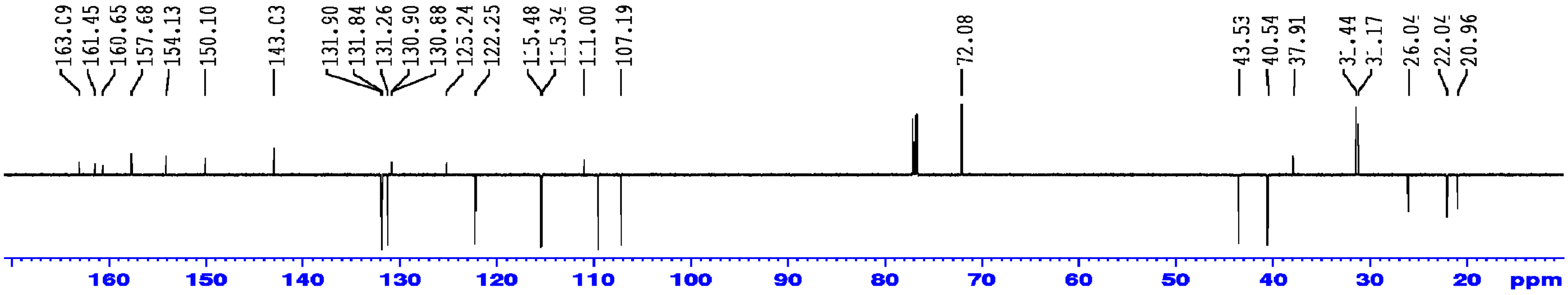

Compound **33d**

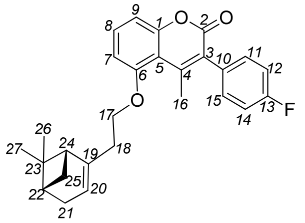

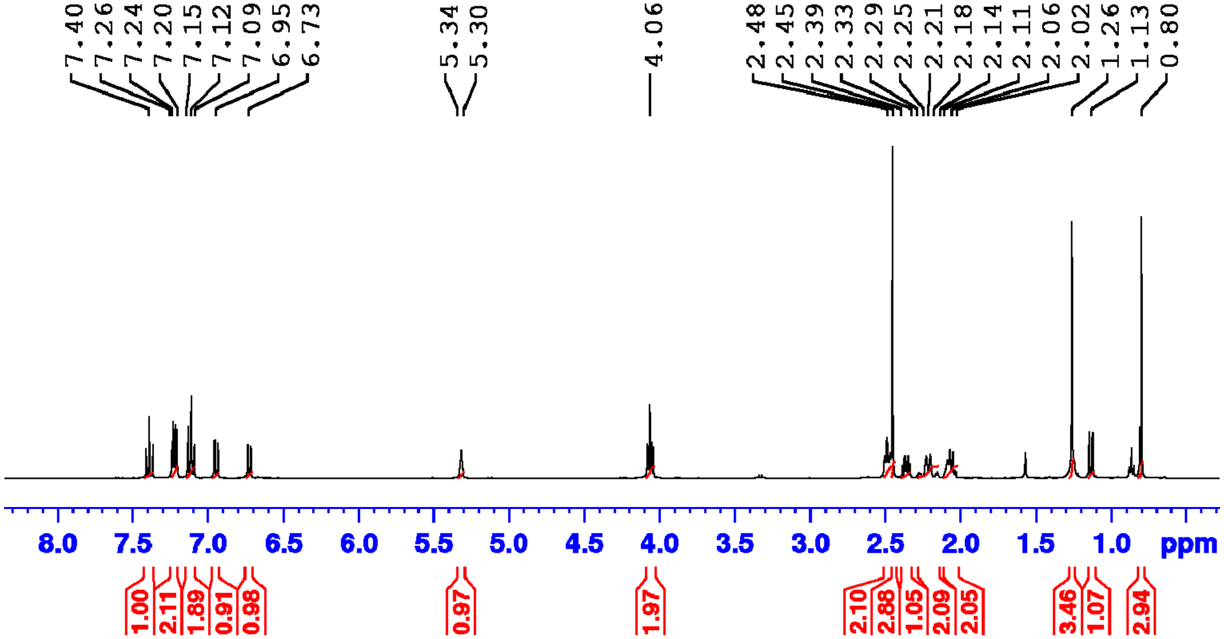


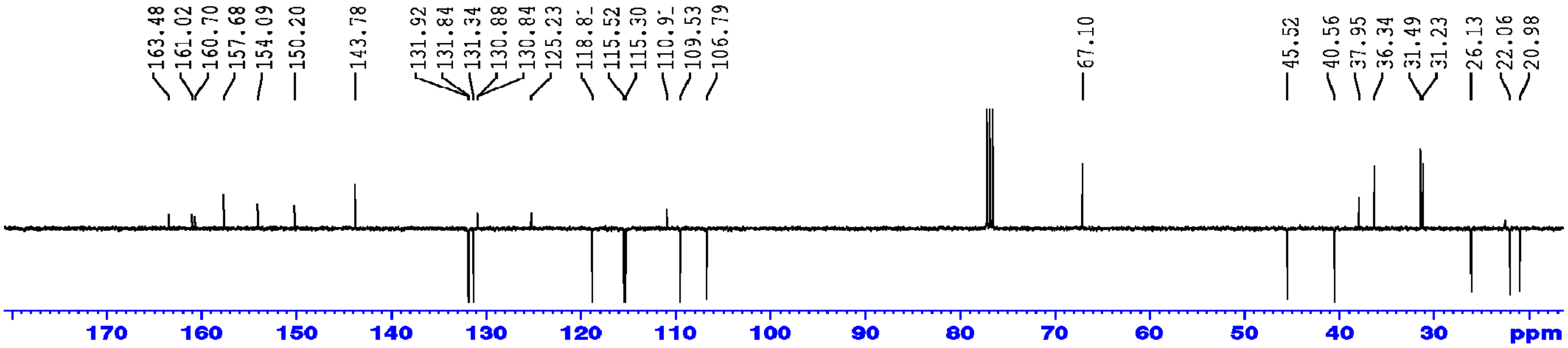

Compound **34a**

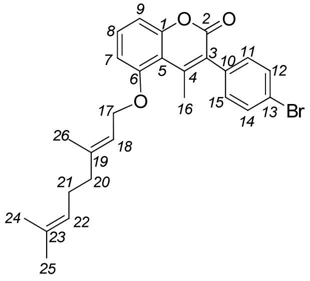


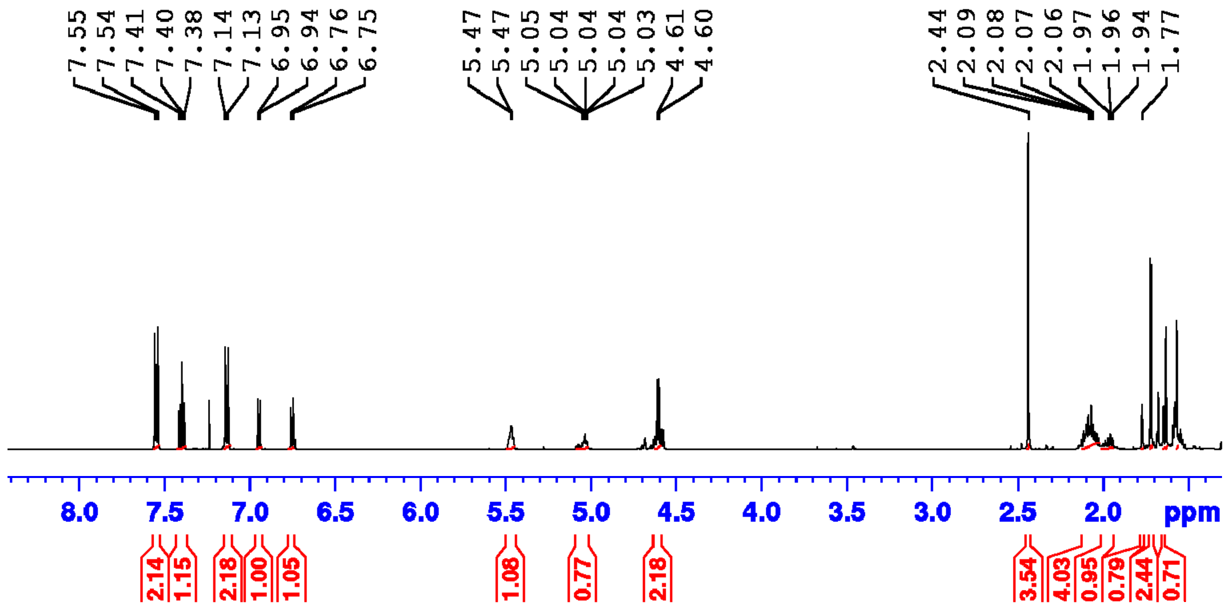


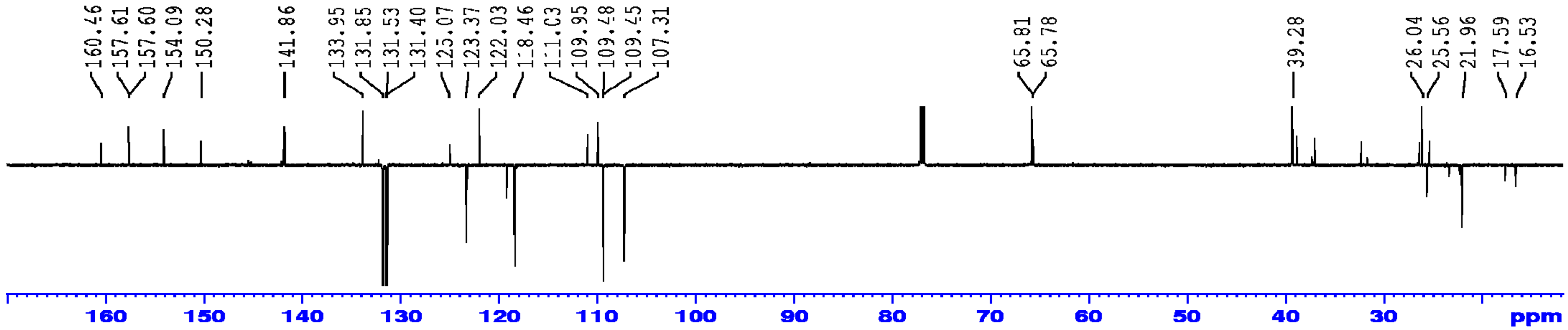

Compound **34b**

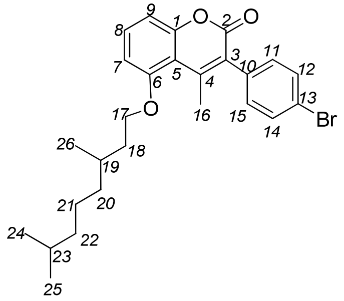


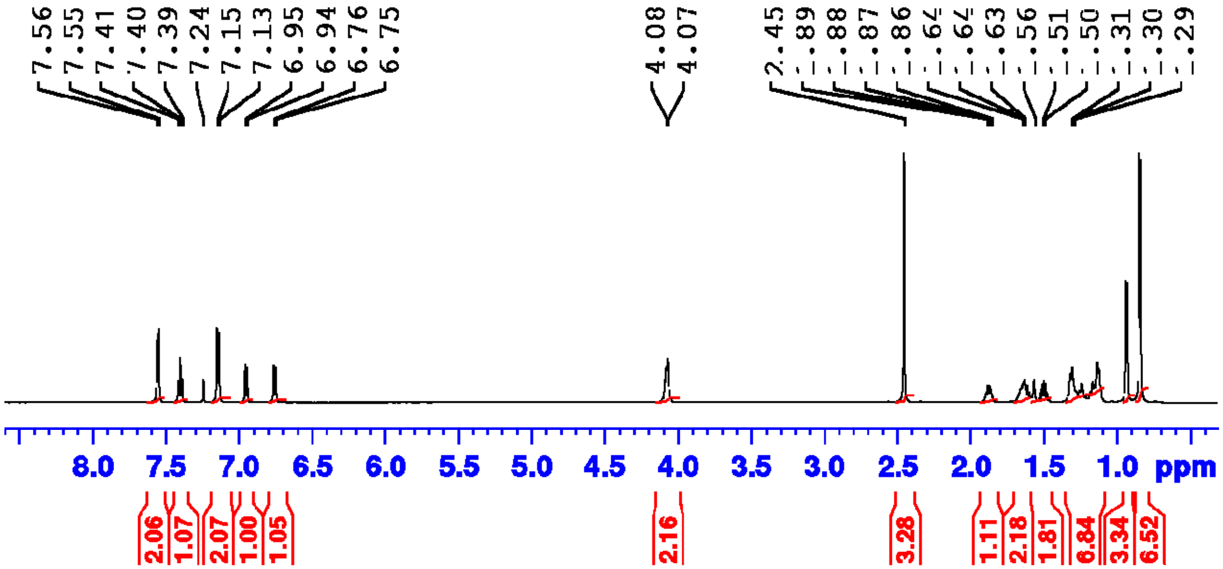


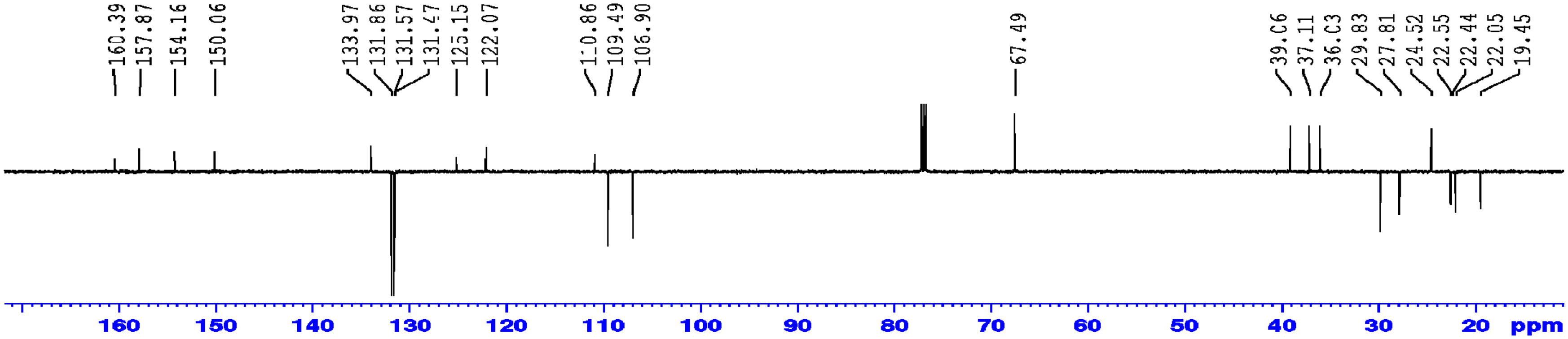

Compound **34c**

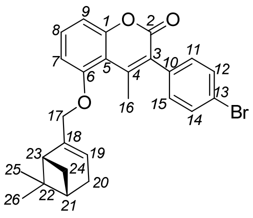


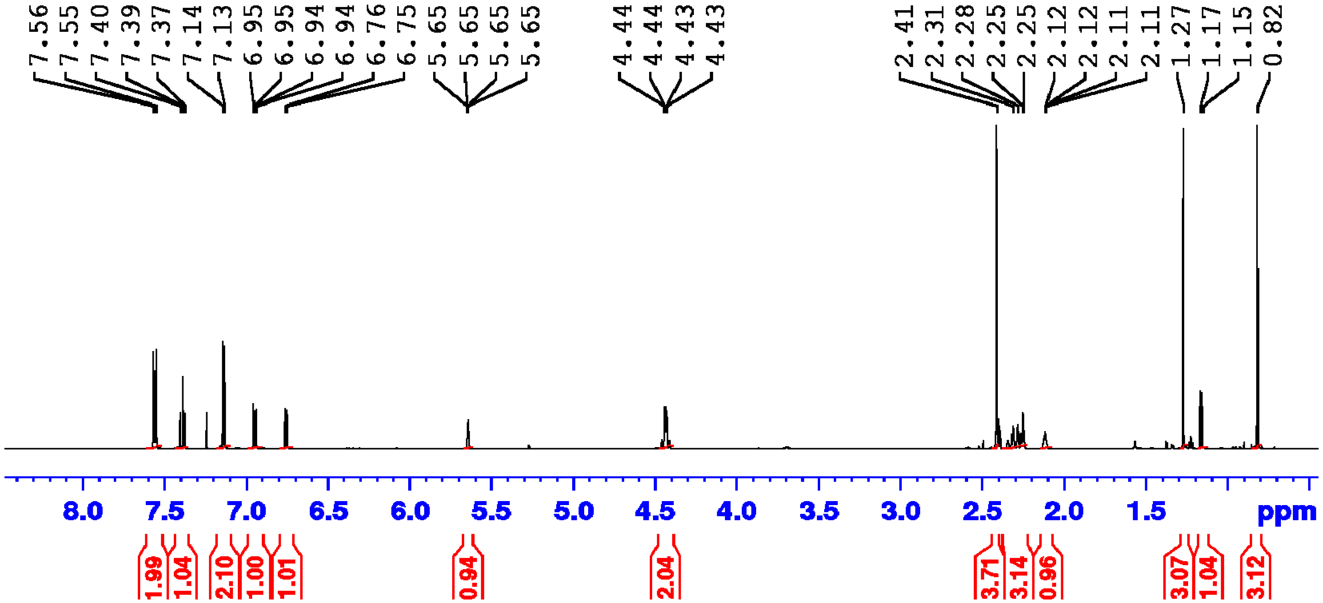


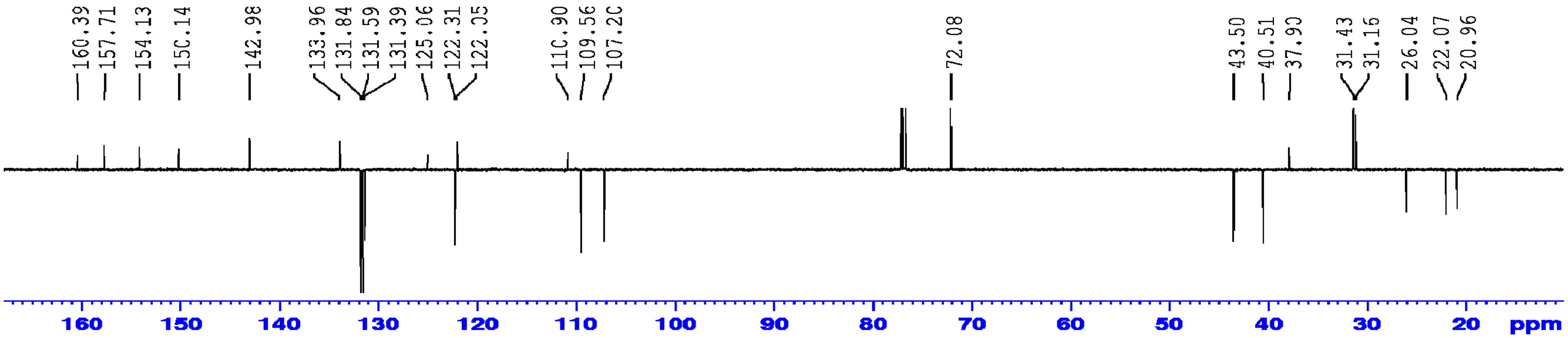

Compound **35a**

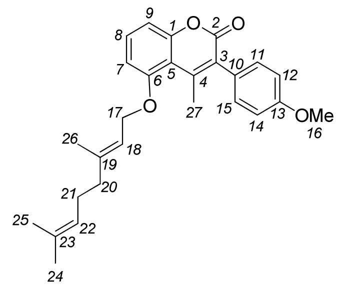


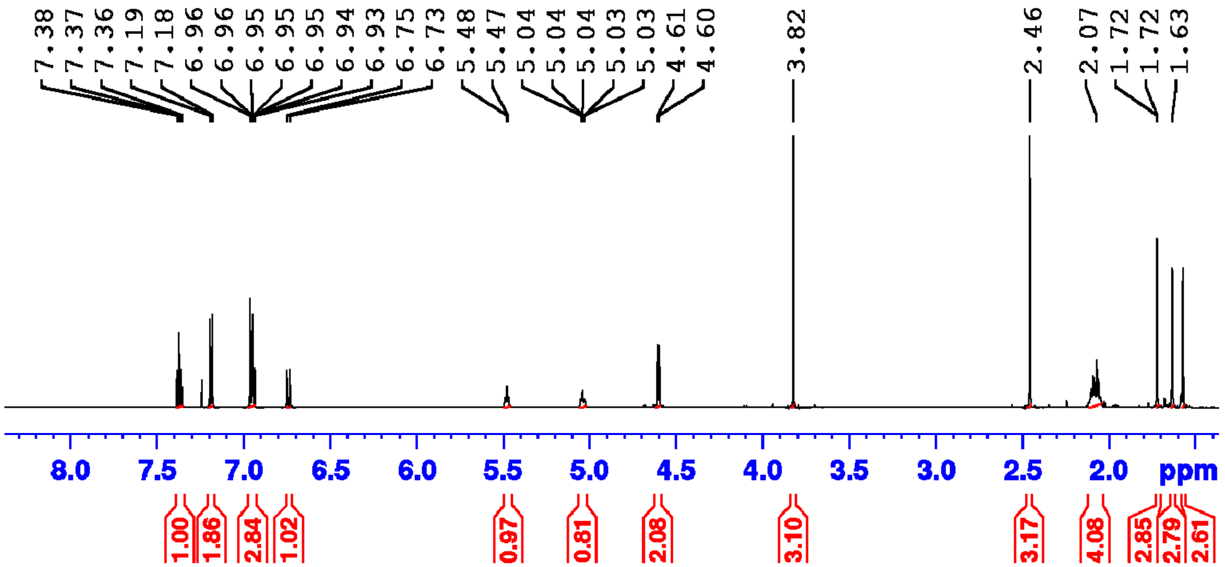


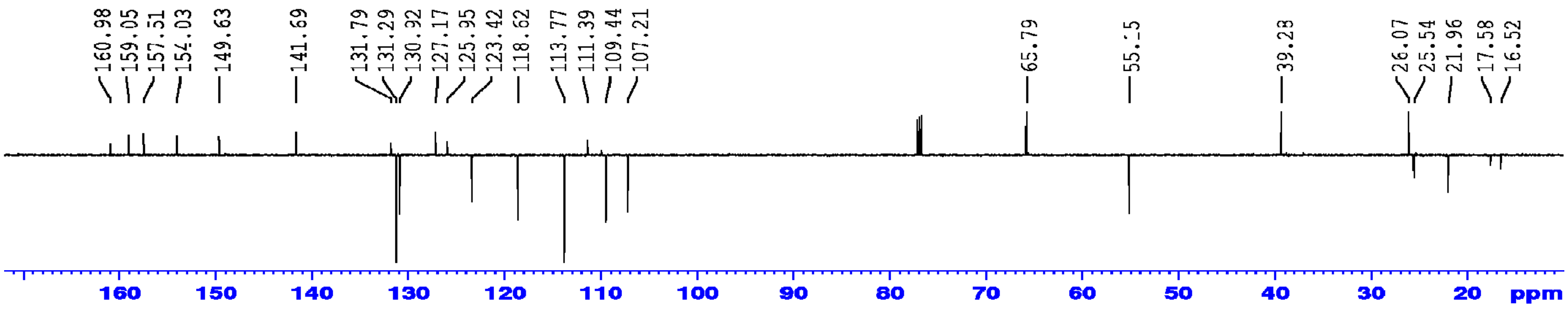

Compound **35b**

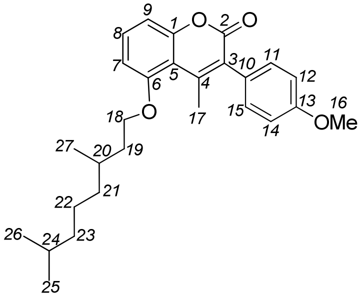


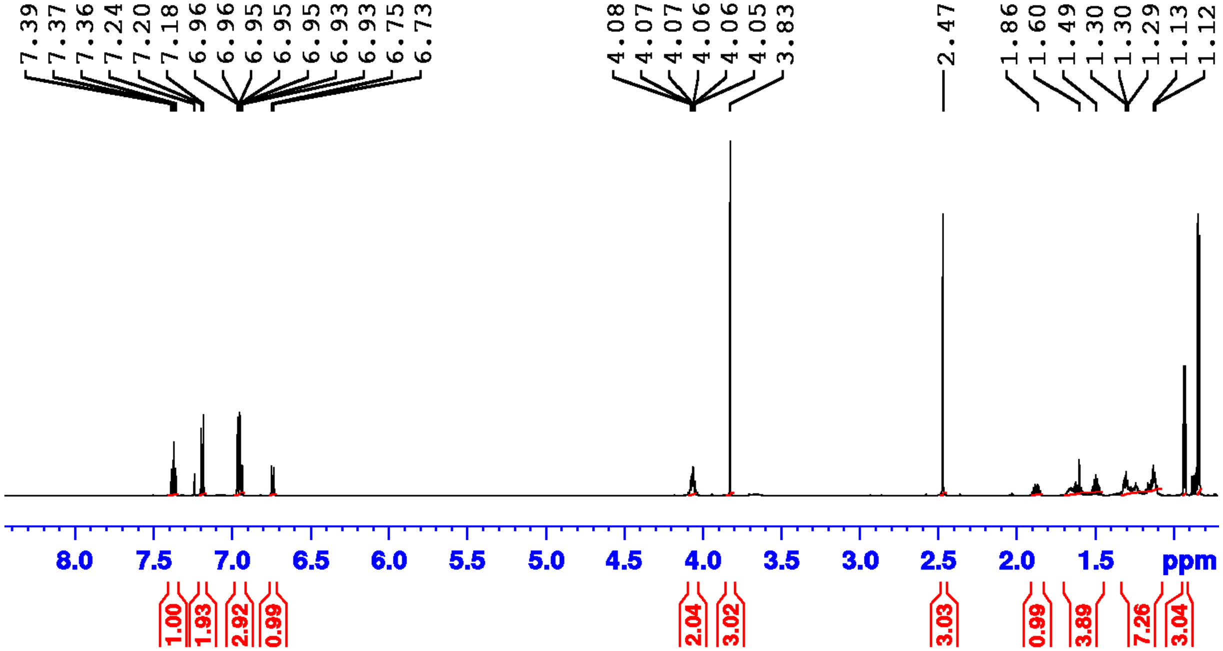


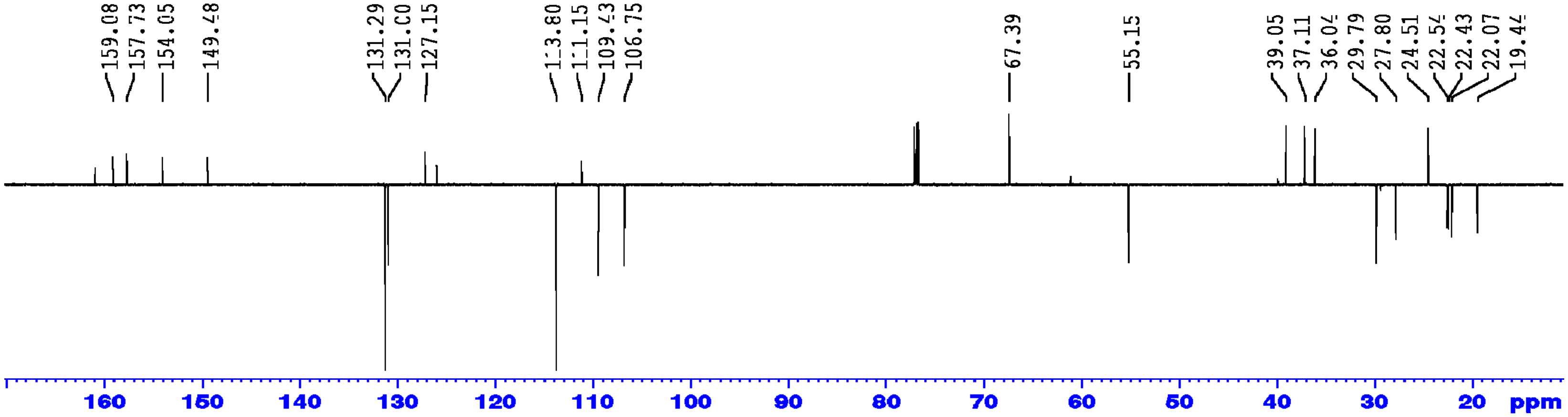

Compound **35c**

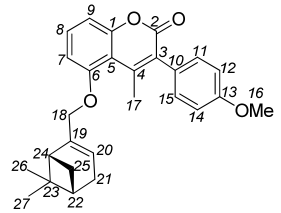


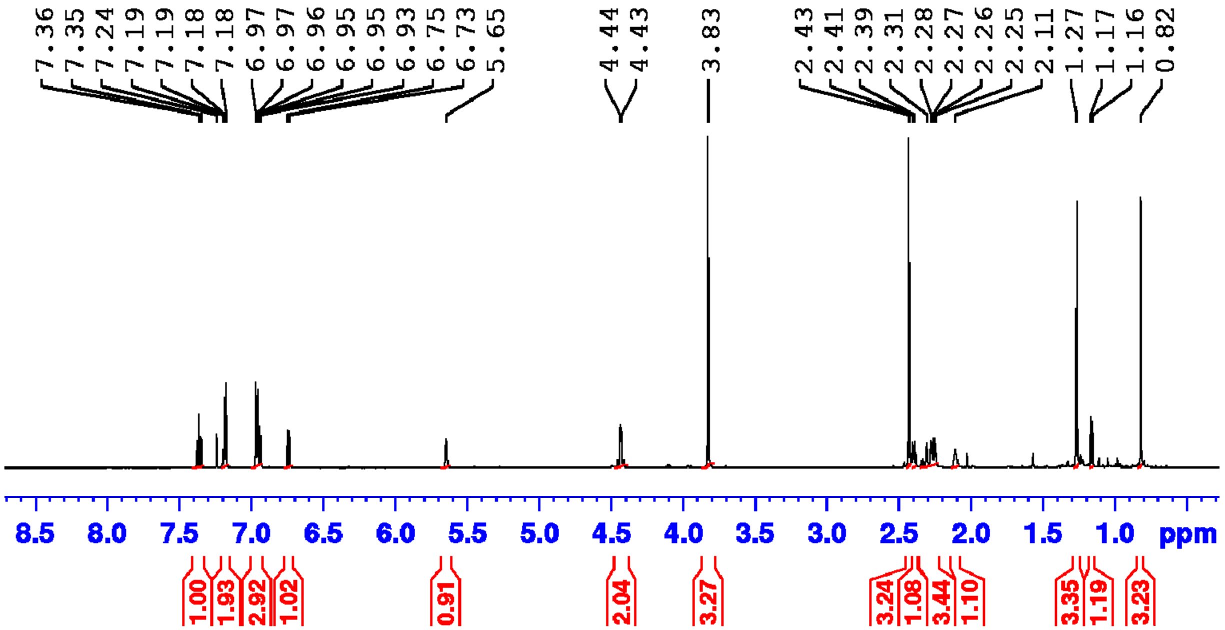


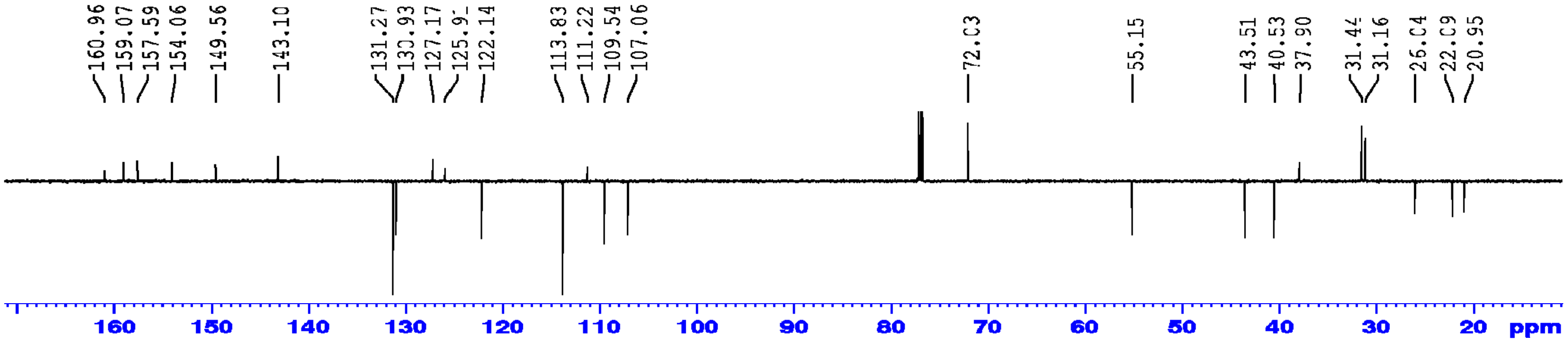

Compound **36a**

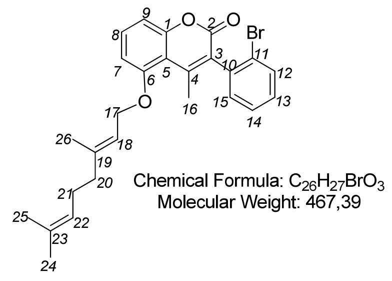


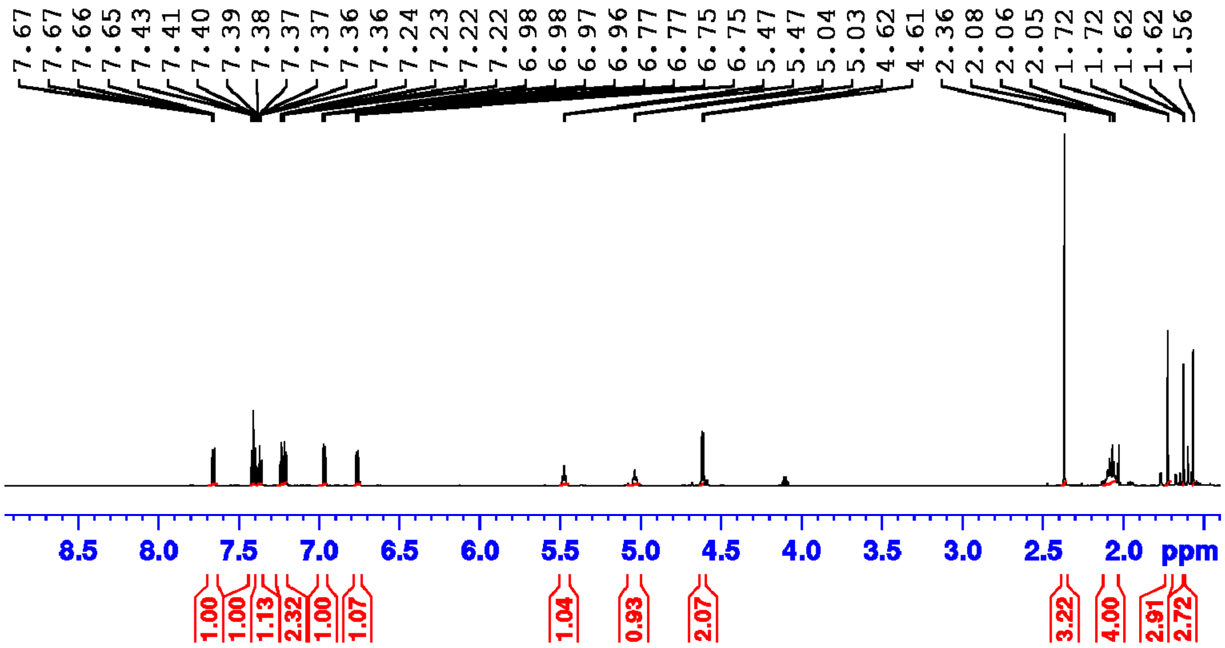

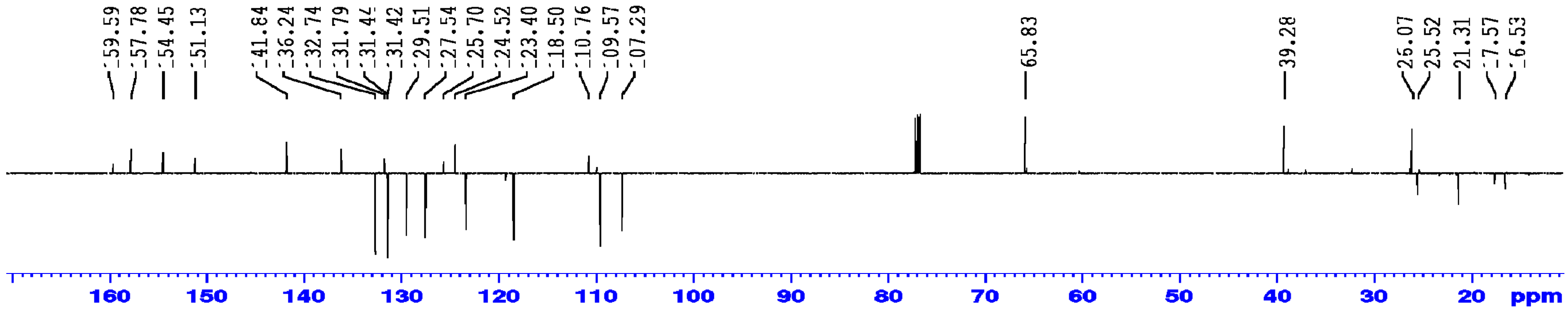

Compound **36b**

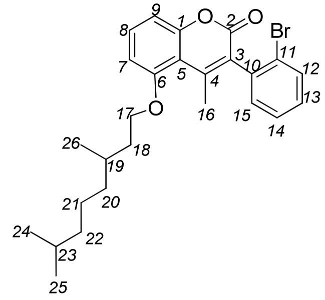

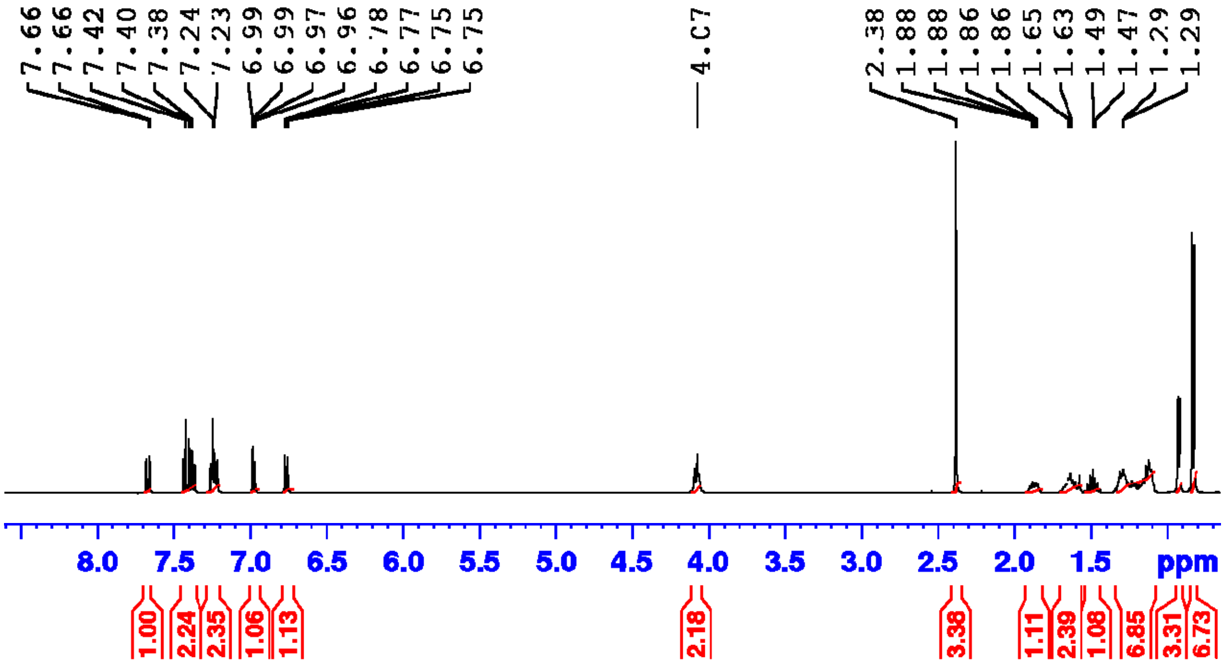


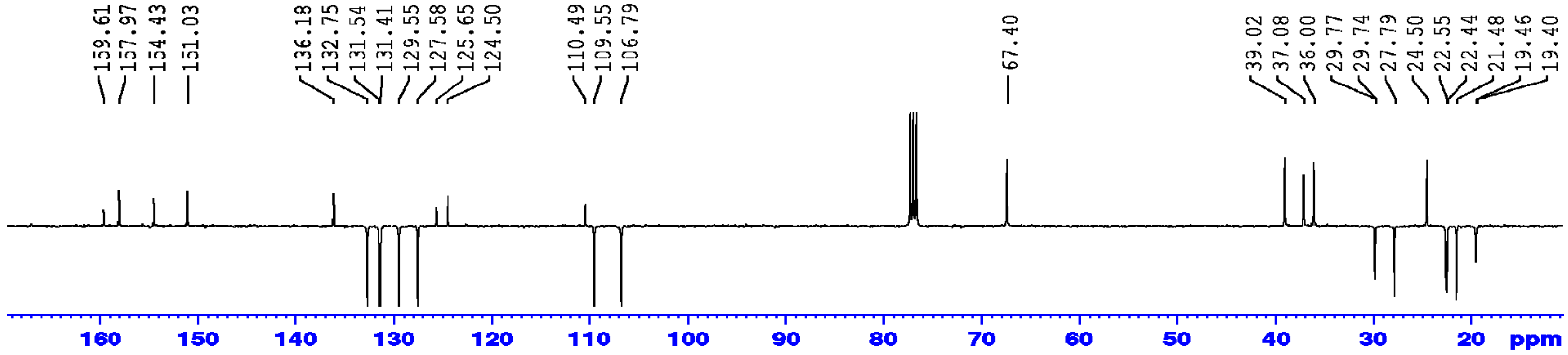

Compound **36c**

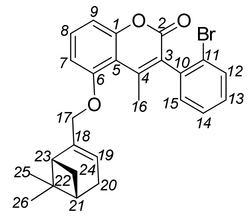


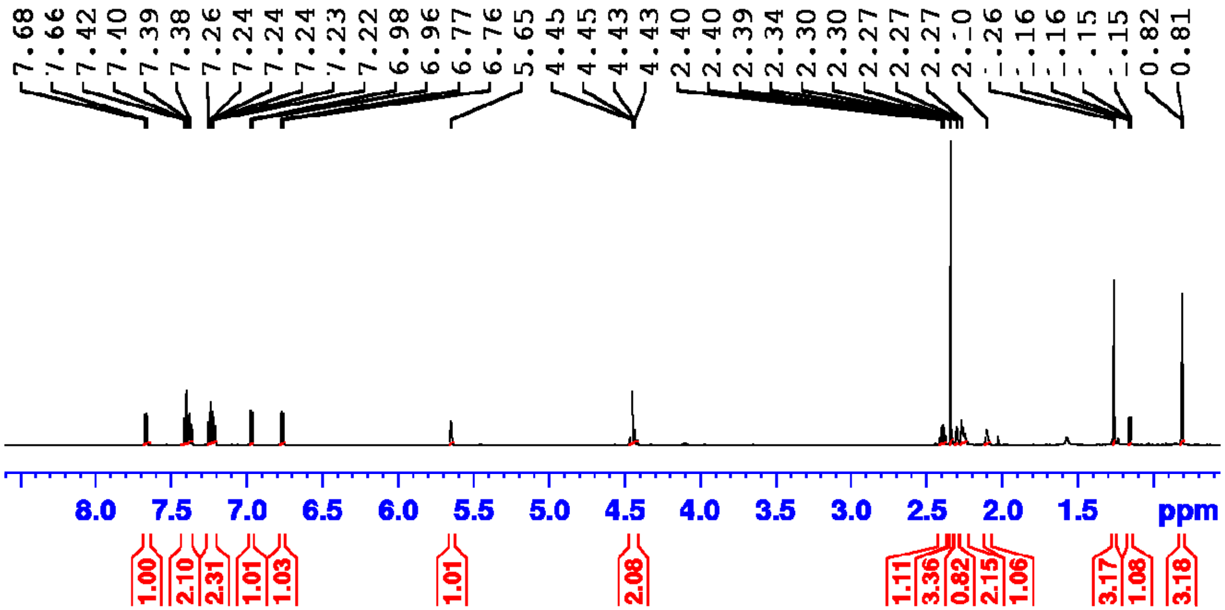


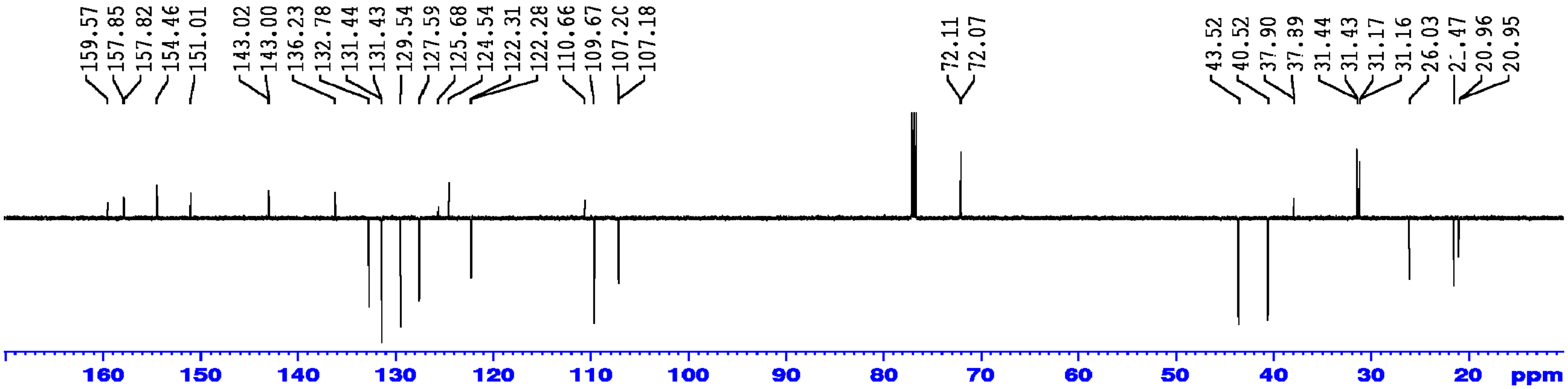

Compound **37a**

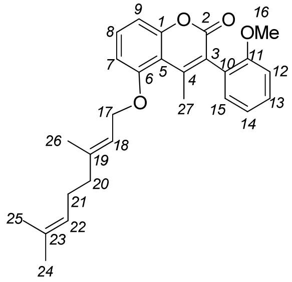


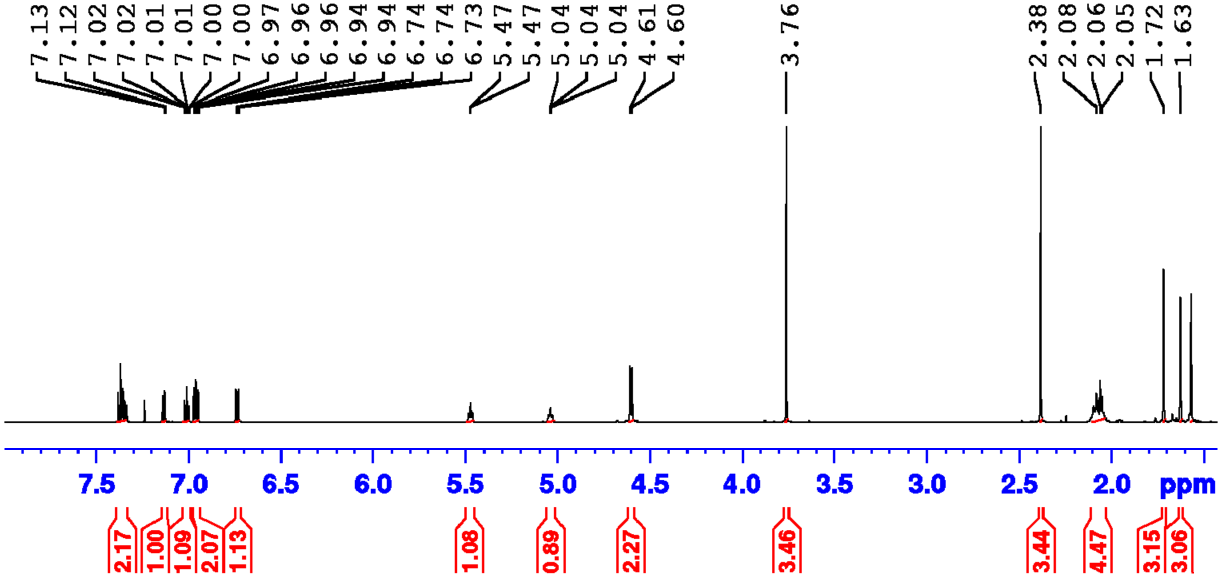


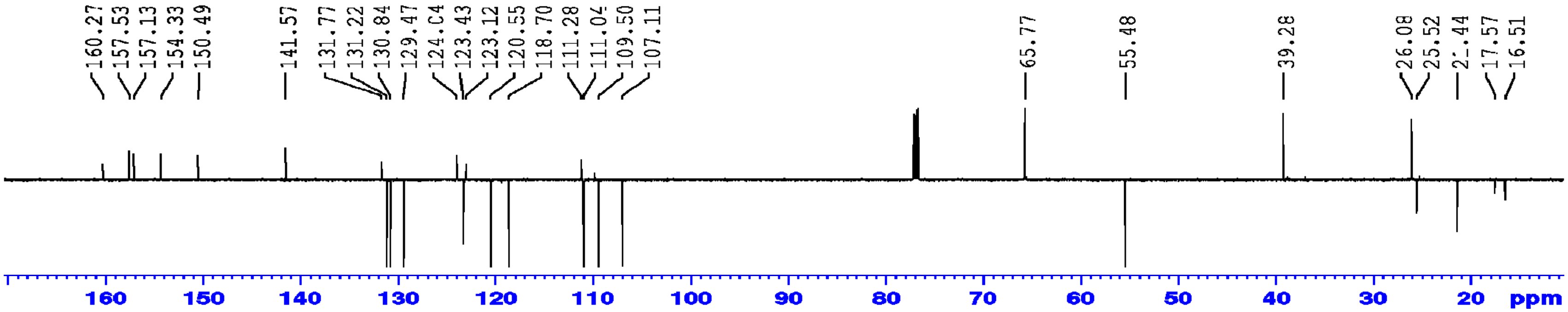

Compound **37b**

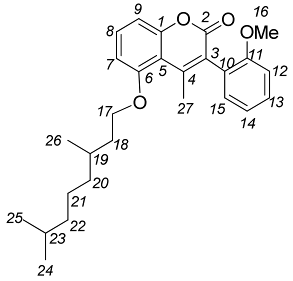


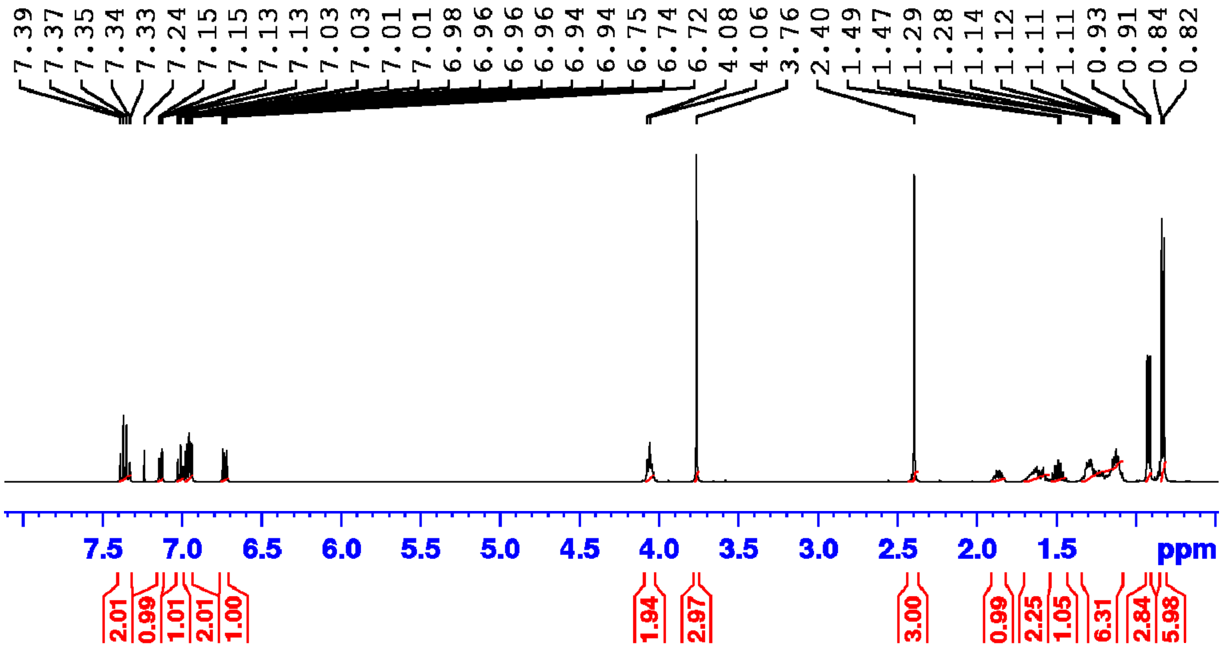


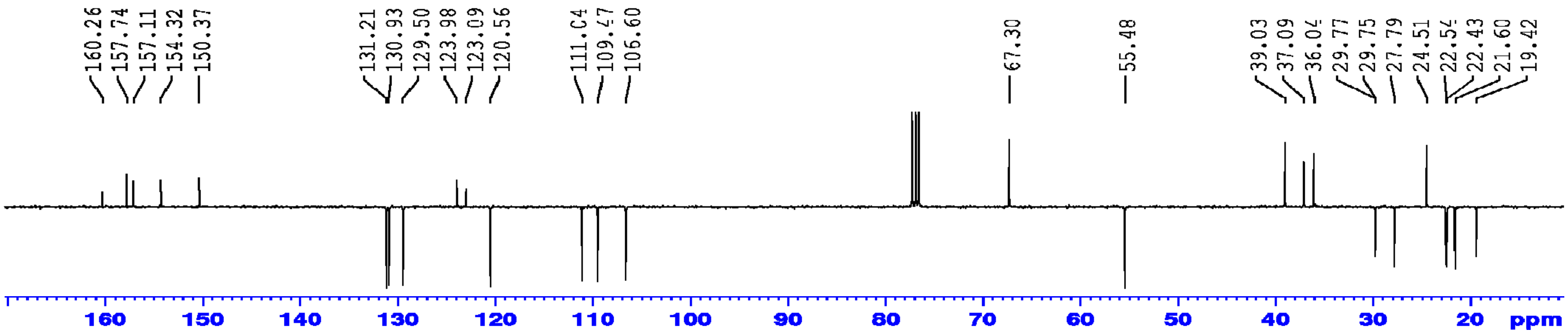

Compound **37c**

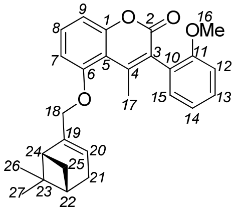


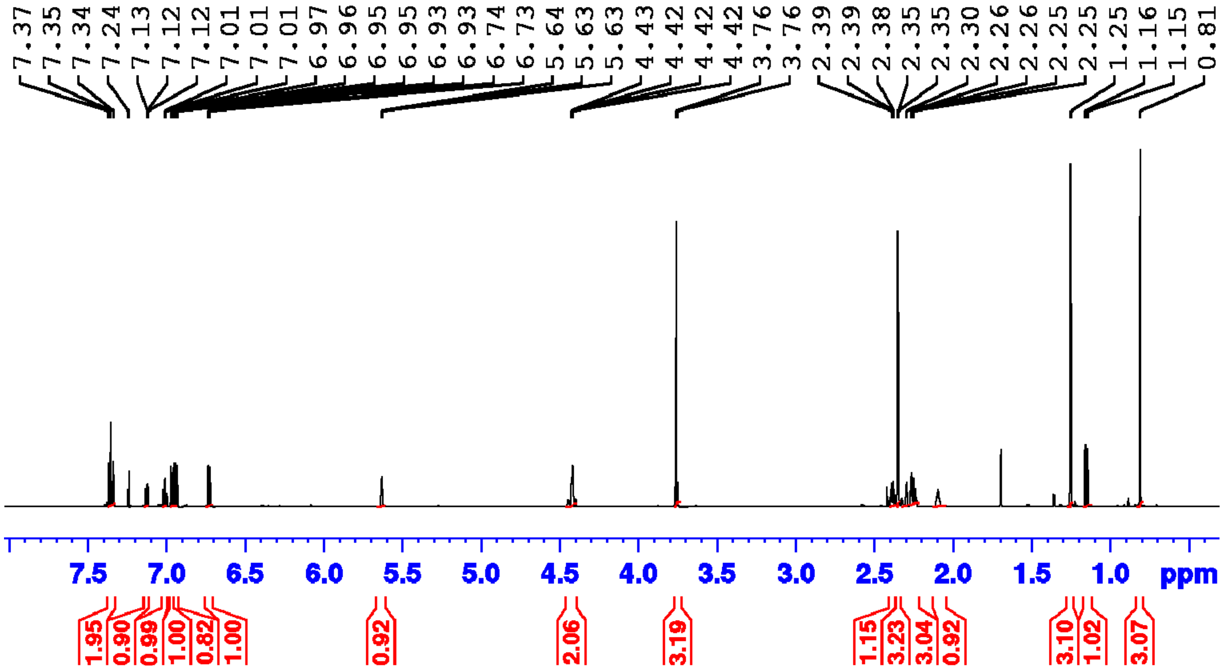


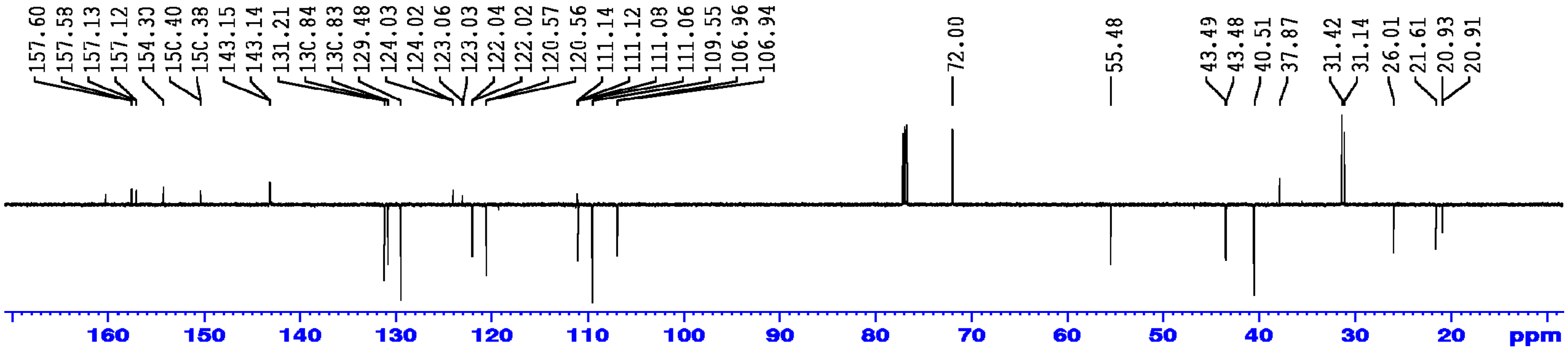

Compound **37d**

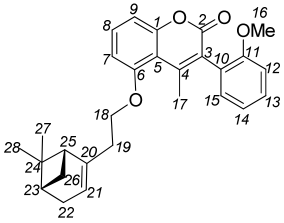


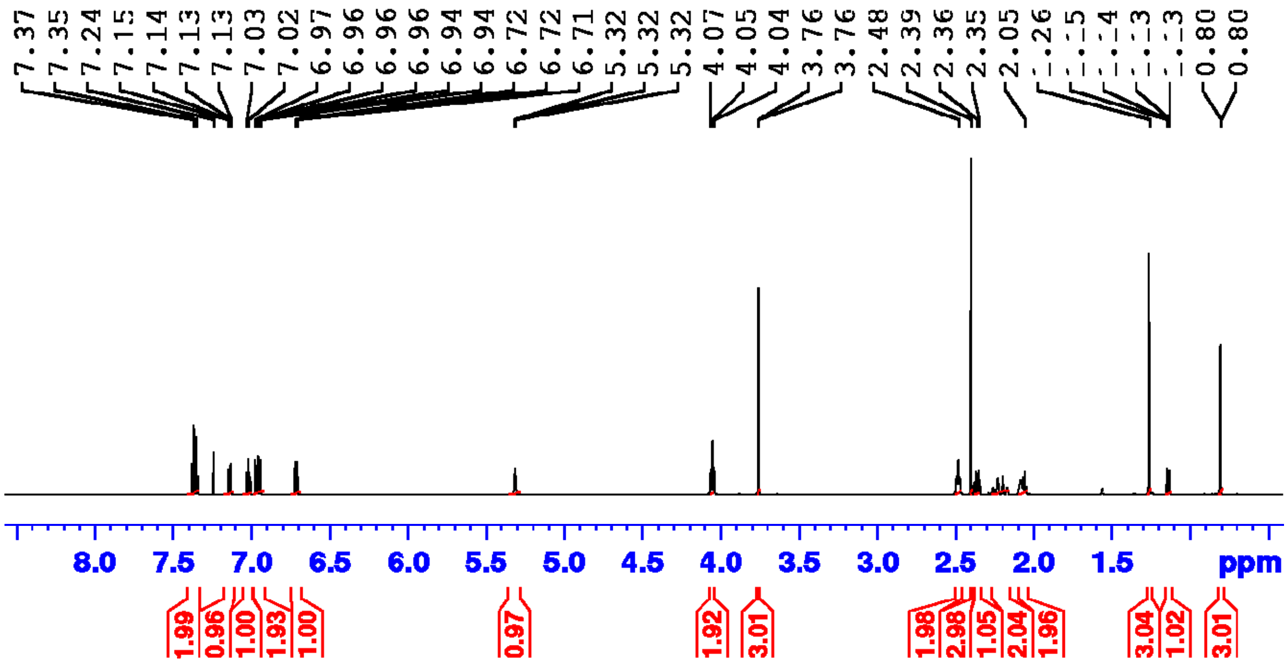


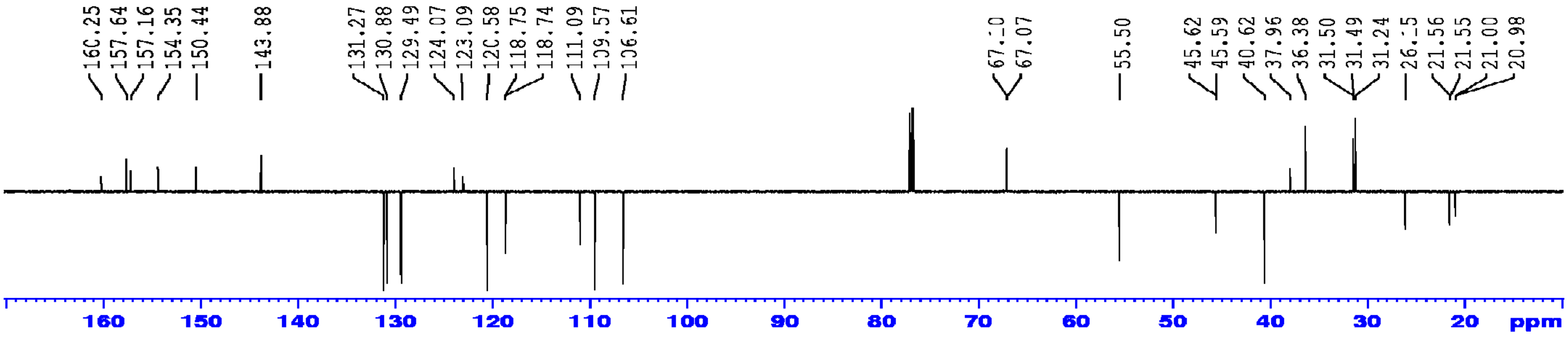

Compound **38a**

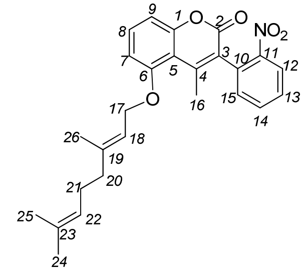


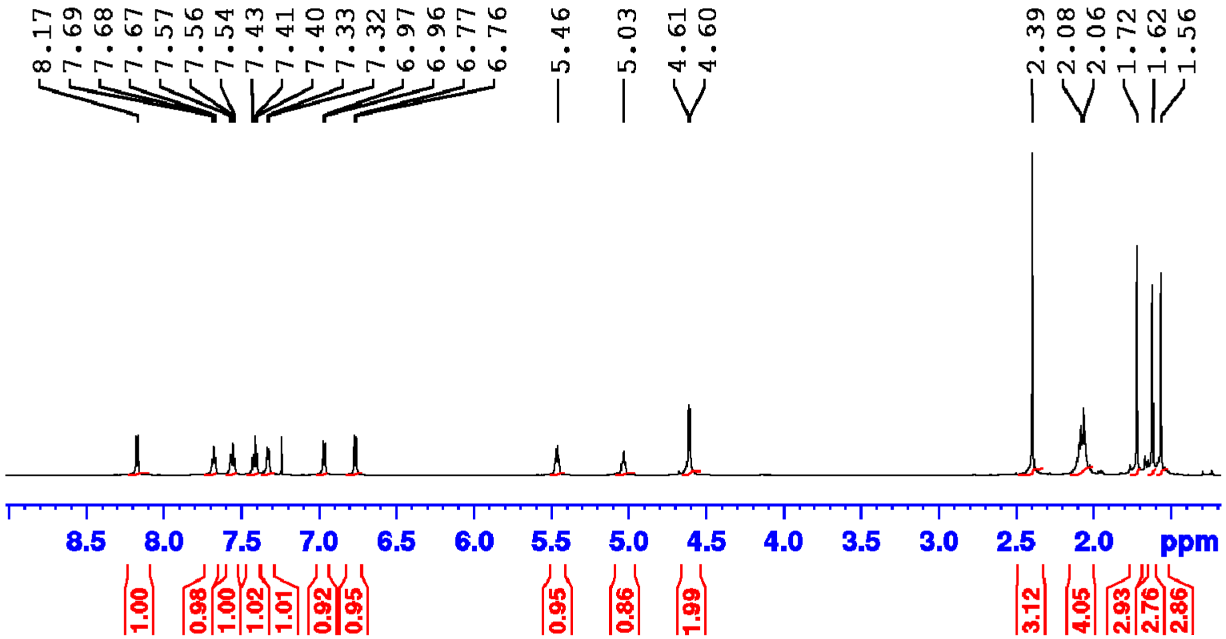


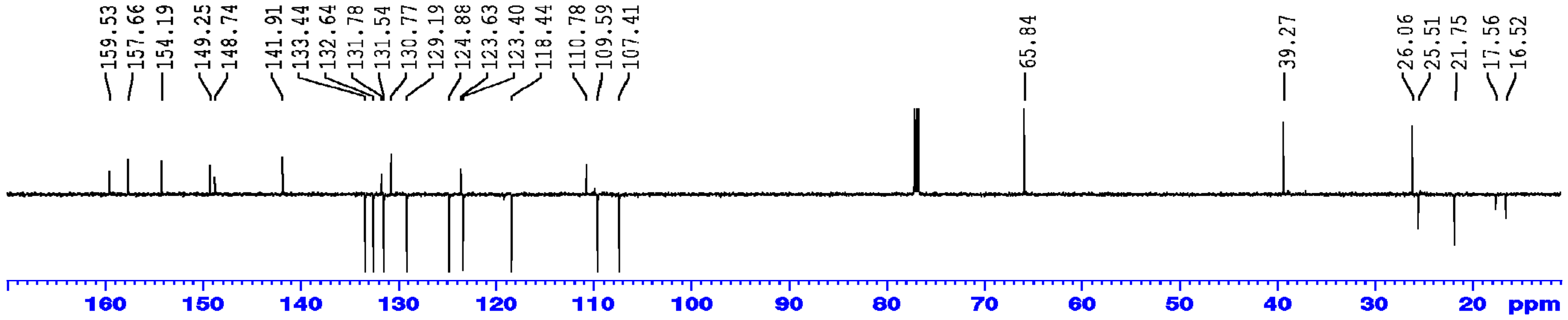

Compound **38b**

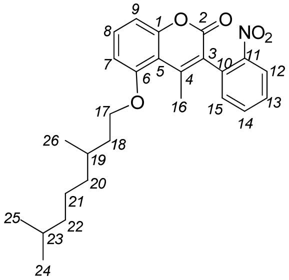


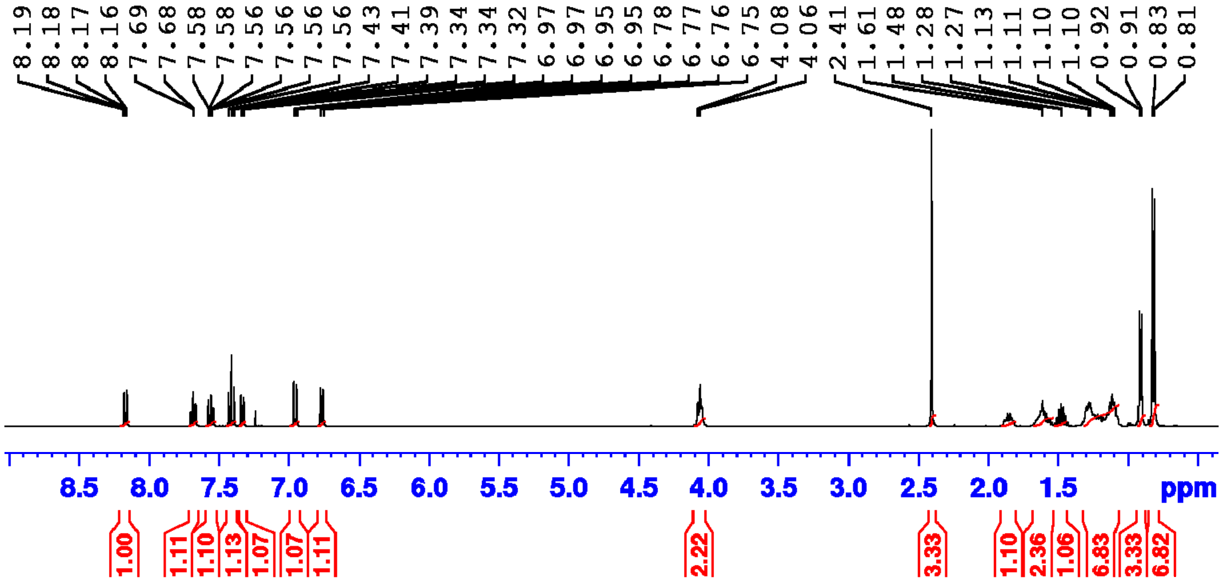


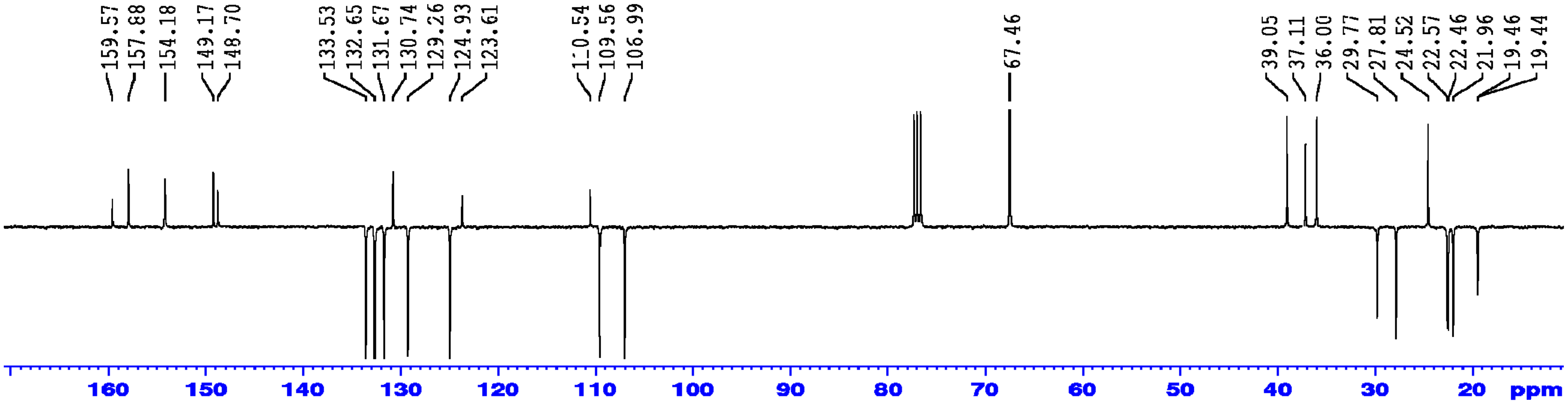

Compound **38c**

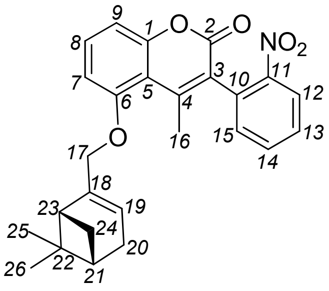

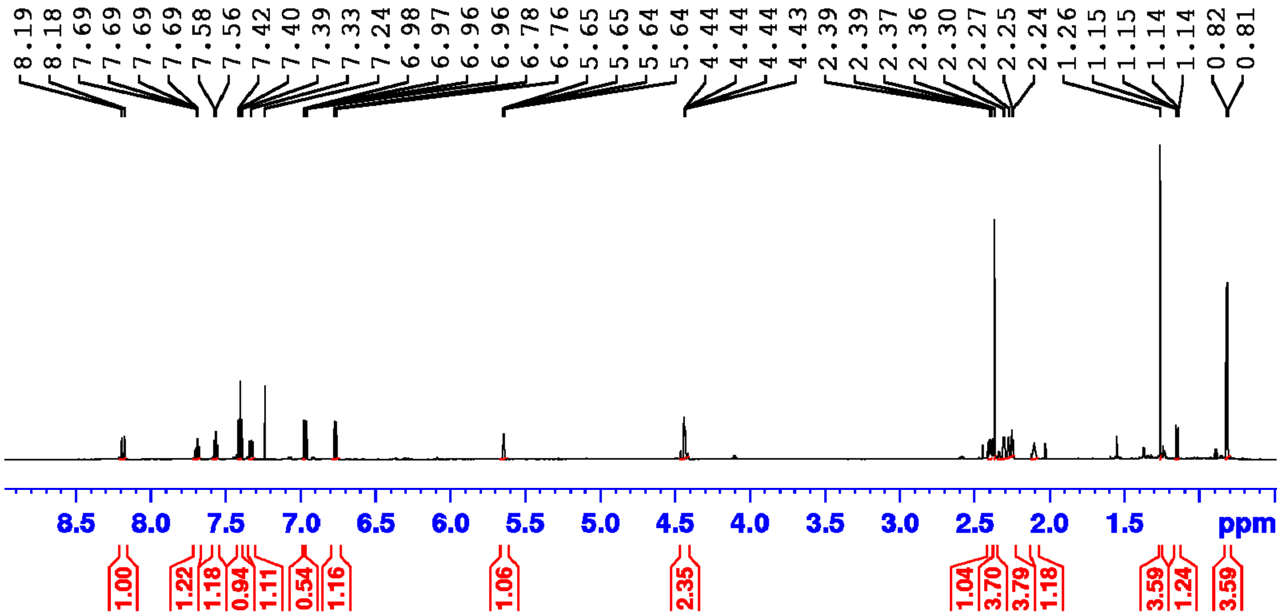

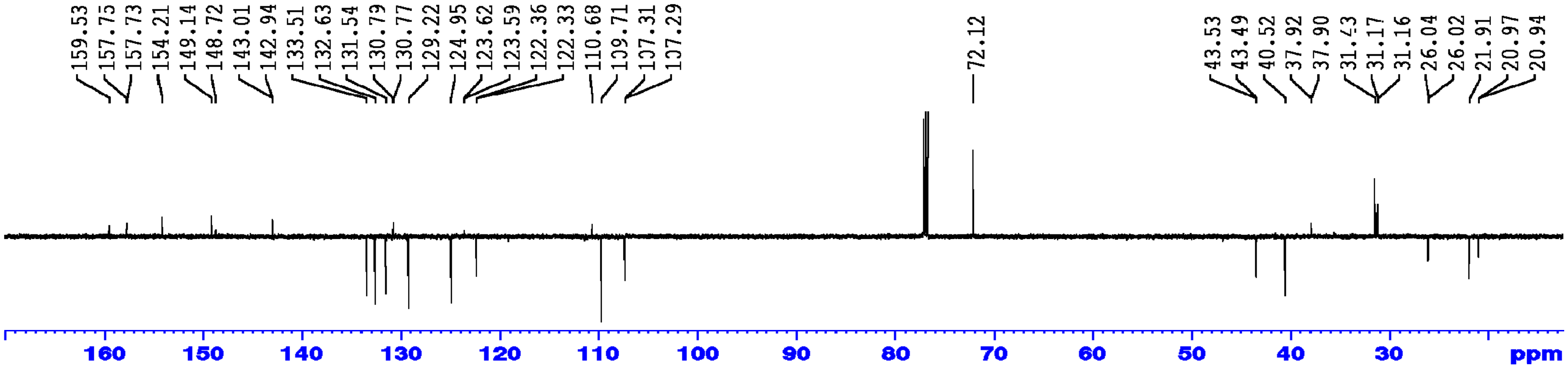

**Table S1.** The binding affinities as predicted by the scoring functions used to the catalytic Tdp1 binding pocket and their measured $IC_{50}$ values.

| Ligand | ASP | ChemPLP | CS | GS | $IC_{50}$, μM |
|---|---|---|---|---|---|
| **34a** | 39 | 62 | 27.1 | 58.4 | 1.06±0.06 |
| **35a** | 35.9 | 60.8 | 26.1 | 57.2 | 6.63±1.12 |
| **37a** | 40.7 | 66.2 | 28.6 | 55.2 | 1.22±0.04 |
| **38a** | 40.9 | 61 | 25.5 | 52.1 | 1.03±0.08 |
| **33a** | 38.9 | 65.1 | 26.1 | 55.4 | 0.13±0.03 |
| **36a** | 35.9 | 63 | 28 | 61.6 | 0.85±0.15 |
| **35c** | 37.6 | 54.7 | 27 | 47.3 | 1.14±0.15 |
| **34c** | 34.8 | 54.3 | 26.8 | 47.6 | 0.9±0.07 |
| **37c** | 37.7 | 55.5 | 26.9 | 53.6 | 0.79±0.06 |
| **38c** | 37 | 54.4 | 27.2 | 53.5 | 3.83±0.29 |
| **33c** | 37 | 54.6 | 26.5 | 49.8 | 5.25±0.04 |
| **36c** | 35.9 | 54.1 | 29 | 53.3 | 4.36±1.14 |
| **37d** | 42.8 | 60 | 31.1 | 52.4 | 3.93±0.77 |
| **33b** | 39.2 | 62.3 | 25.3 | 52.9 | 0.29±0.07 |
| **35b** | 38.3 | 63.2 | 25.3 | 58.2 | 0.37±0.16 |
| **36b** | 36.5 | 58.1 | 28.4 | 56.3 | ≥50 |
| **37b** | 39.8 | 61.2 | 28.2 | 56.4 | 2.60±0.35 |
| **38b** | 41.2 | 62.7 | 28.0 | 58.3 | 2.18±0.13 |
| **33d** | 36.8 | 57.6 | 27.7 | 52.6 | 2.51±0.14 |

**Table S2.** The molecular descriptors and their corresponding Known Drug Indexes 2a and 2b ($KDI_{2a/2b}$).

| | RB | MW | HD | HA | Log P | PSA | $KDI_{2A}$ | $KDI_{2B}$ | $IC_{50}$, μM |
|---|---|---|---|---|---|---|---|---|---|
| **34a** | 8 | 418.5 | 0 | 4 | 6.8 | 44.8 | 3.92 | 0.04 | 1.06±0.06 |
| **35a** | 8 | 433.5 | 0 | 4.25 | 6.0 | 79.0 | 4.25 | 0.09 | 6.63±1.12 |
| **37a** | 7 | 406.5 | 0 | 3.25 | 6.7 | 36.1 | 3.90 | 0.04 | 1.22±0.04 |
| **38a** | 7 | 467.4 | 0 | 3.25 | 7.1 | 40.2 | 3.63 | 0.02 | 1.03±0.08 |
| **33a** | 5 | 416.5 | 0 | 4 | 6.0 | 46.9 | 4.48 | 0.12 | 0.13±0.03 |
| **36a** | 4 | 465.4 | 0 | 3.25 | 6.5 | 39.4 | 3.95 | 0.05 | 0.85±0.15 |
| **35c** | 5 | 416.5 | 0 | 4 | 6.1 | 44.5 | 4.44 | 0.11 | 1.14±0.15 |
| **34c** | 5 | 431.5 | 0 | 4.25 | 5.3 | 78.6 | 4.81 | 0.22 | 0.9±0.07 |
| **37c** | 4 | 404.5 | 0 | 3.25 | 6.2 | 39.4 | 4.26 | 0.09 | 0.79±0.06 |
| **38c** | 4 | 465.4 | 0 | 3.25 | 6.4 | 39.4 | 3.96 | 0.05 | 3.83±0.29 |
| **33c** | 6 | 430.5 | 0 | 4 | 6.4 | 44.6 | 4.24 | 0.08 | 5.25±0.04 |
| **36c** | 9 | 410.5 | 0 | 3.25 | 7.0 | 39.6 | 3.55 | 0.02 | 4.36±1.14 |
| **37d** | 10 | 422.6 | 0 | 4 | 6.8 | 47.0 | 3.60 | 0.02 | 3.93±0.77 |
| **33b** | 7 | 467.4 | 0 | 3.25 | 7.0 | 36.1 | 3.59 | 0.02 | 0.29±0.07 |
| **35b** | 8 | 418.5 | 0 | 4 | 6.7 | 47.0 | 3.95 | 0.05 | 0.37±0.16 |
| **36b** | 9 | 457.4 | 0 | 3.25 | 6.9 | 44.4 | 3.41 | 0.02 | ≥50 |
| **37b** | 10 | 408.5 | 0 | 4 | 6.6 | 48.8 | 3.71 | 0.03 | 2.60±0.35 |
| **38b** | 10 | 423.5 | 0 | 4.25 | 5.8 | 83.2 | 4.01 | 0.05 | 2.18±0.13 |
| **33d** | 5 | 404.5 | 0 | 3.25 | 6.2 | 43.2 | 4.28 | 0.09 | 2.51±0.14 |

**Table S3**. Definition of lead-like, drug-like and Known Drug Space (KDS) in terms of molecular descriptors. The values given are the maxima for each descriptor for the volumes of chemical space used.

| | **Lead-like Space** | **Drug-like Space** | **Known Drug Space** |
|---|---|---|---|
| Molecular weight (g $mol^{-1}$) | 300 | 500 | 800 |
| Lipophilicity (Log P) | 3 | 5 | 6.5 |
| Hydrogen bond donors (HD) | 3 | 5 | 7 |
| Hydrogen bond acceptors (HA) | 3 | 10 | 15 |
| Polar surface area ($Å^2$) (PSA) | 60 | 140 | 180 |
| Rotatable bonds (RB) | 3 | 10 | 17 |
